# Deep-learning based detection of COVID-19 using lung ultrasound imagery

**DOI:** 10.1371/journal.pone.0255886

**Published:** 2021-08-13

**Authors:** Julia Diaz-Escobar, Nelson E. Ordóñez-Guillén, Salvador Villarreal-Reyes, Alejandro Galaviz-Mosqueda, Vitaly Kober, Raúl Rivera-Rodriguez, Jose E. Lozano Rizk

**Affiliations:** 1 CICESE Research Center, Ensenada, Baja California, México; 2 Faculty of Science, UABC, Ensenada, Baja California, México; 3 Department of Mathematics, Chelyabinsk State University, Chelyabinsk, Russia; Politechnika Slaska, POLAND

## Abstract

**Background:**

The COVID-19 pandemic has exposed the vulnerability of healthcare services worldwide, especially in underdeveloped countries. There is a clear need to develop novel computer-assisted diagnosis tools to provide rapid and cost-effective screening in places where massive traditional testing is not feasible. Lung ultrasound is a portable, easy to disinfect, low cost and non-invasive tool that can be used to identify lung diseases. Computer-assisted analysis of lung ultrasound imagery is a relatively recent approach that has shown great potential for diagnosing pulmonary conditions, being a viable alternative for screening and diagnosing COVID-19.

**Objective:**

To evaluate and compare the performance of deep-learning techniques for detecting COVID-19 infections from lung ultrasound imagery.

**Methods:**

We adapted different pre-trained deep learning architectures, including VGG19, InceptionV3, Xception, and ResNet50. We used the publicly available POCUS dataset comprising 3326 lung ultrasound frames of healthy, COVID-19, and pneumonia patients for training and fine-tuning. We conducted two experiments considering three classes (COVID-19, pneumonia, and healthy) and two classes (COVID-19 versus pneumonia and COVID-19 versus non-COVID-19) of predictive models. The obtained results were also compared with the POCOVID-net model. For performance evaluation, we calculated per-class classification metrics (Precision, Recall, and F1-score) and overall metrics (Accuracy, Balanced Accuracy, and Area Under the Receiver Operating Characteristic Curve). Lastly, we performed a statistical analysis of performance results using ANOVA and Friedman tests followed by post-hoc analysis using the Wilcoxon signed-rank test with the Holm’s step-down correction.

**Results:**

InceptionV3 network achieved the best average accuracy (89.1%), balanced accuracy (89.3%), and area under the receiver operating curve (97.1%) for COVID-19 detection from bacterial pneumonia and healthy lung ultrasound data. The ANOVA and Friedman tests found statistically significant performance differences between models for accuracy, balanced accuracy and area under the receiver operating curve. Post-hoc analysis showed statistically significant differences between the performance obtained with the InceptionV3-based model and POCOVID-net, VGG19-, and ResNet50-based models. No statistically significant differences were found in the performance obtained with InceptionV3- and Xception-based models.

**Conclusions:**

Deep learning techniques for computer-assisted analysis of lung ultrasound imagery provide a promising avenue for COVID-19 screening and diagnosis. Particularly, we found that the InceptionV3 network provides the most promising predictive results from all AI-based techniques evaluated in this work. InceptionV3- and Xception-based models can be used to further develop a viable computer-assisted screening tool for COVID-19 based on ultrasound imagery.

## Introduction

In December 2019, a novel coronavirus, named SARS-CoV-2, emerged in Wuhan, China, which caused the COVID-19 disease when infecting humans. COVID-19 is a serious illness that can lead to the death of the infected host [1]. The threat posed by COVID-19 led the World Health Organization (WHO) to declare the COVID-19 pandemic by March 2020 [2].

Coronaviruses are a group of highly diverse, enveloped, positive-sense, single-stranded RNA viruses and are widely spread in birds and mammals. Sometimes these viruses infect humans, causing mild to moderate respiratory diseases [3]. Before SARS-CoV-2, two coronaviruses were known to cause severe human disease: SARS-CoV, which causes Severe Acute Respiratory Syndrome (SARS); and MERS-CoV, which causes Middle East Respiratory Syndrome (MERS) [4, 5]. However, in contrast to SARS and MERS, the symptom onset for COVID-19 is significantly larger, or it may appear in a mild form, allowing infection spread by asymptomatic patients, which in turn has led to the current pandemic [6]. Although the WHO has emphasized the need for massive testing and contact tracing to better tackle the pandemic, not all countries have the required laboratory infrastructure and reagents to effectively address this task. Additionally, getting results from some of these tests may take a couple of days, leading to non-confirmed COVID-19 patients with mild or no symptoms to further spread the disease while waiting for the test results.

With the rise of deep learning techniques, medical imagery has increasingly claimed attention for the computed assisted analysis of pulmonary conditions. Automated analysis of Computed Tomography (CT) scans, has enabled the identification of malignant nodules [7]. Radiographic analysis, in turn, has also obtained fair results in the detection of tuberculosis signs [8], as well as other multiple cardiothoracic abnormalities [9, 10].

From the beginning of the COVID-19 pandemics, the research community considered CT and X-ray imagery as alternatives for the burdened RT-PCR testing. Indeed, early works on COVID-19 imagery identified the existence of pulmonary lesions in non-severe and even in recovered patients [11]. In this manner, Akram *et al*. [12] preprocessed CT data by proposing extraction and selection schemes of relevant features to classify COVID-19 and normal scans. They tested several classifiers obtaining an accuracy of 92.6% with a Naive Bayes classifier. Albahli [13] distinguished COVID-19 from other seven diseases by using X-ray images augmented with Generative Adversarial Networks to solve the class imbalance problem. They used some deep neural networks to achieve an accuracy of 87%. Narin *et al*. [14] implemented three binary classification schemes to discriminate COVID-19 from normal, bacterial pneumonia, and viral pneumonia X-ray datasets. Five different pre-trained convolutional neural networks were tested to produce the best accuracies of 96.1%, 99.5%, and 99.7%, respectively, for each classification scheme. The most prominent work based on X-ray images was developed by Wang *et al*. [15]. They devised COVID-net, a convolutional architecture trained with a dataset comprising 13,975 X-ray images. COVID-net attained an accuracy of 93.3% and sensitivities of 95%, 94%, and 91%, for normal, non-COVID, and COVID-19 infection, respectively. For other results regarding computer-assisted analysis of CT and X-ray for detection of COVID-19, the reader may refer to [16–18].

Although research on the use of computer-assisted analysis of CT and X-ray imaging for lung disease screening has yielded promising results, the practical application of these imaging technologies for massive COVID-19 screening has some drawbacks. In the context of the current pandemic, issues such as the cost of CT and X-ray equipment, portability, accessibility, and sterilization procedures, may hinder the applicability and impact of AI-based analysis of CT and X-ray imaging for COVID-19 screening.

Lung ultrasound (LUS) is a portable, easy to disinfect, low cost and non-invasive medical imaging tool that can be used to identify lung diseases as an alternative to CT and X-ray [19–23]. Computer-assisted analysis of lung ultrasound imagery is a relatively recent approach that has shown great potential for diagnosing pulmonary conditions (e.g., [24–31]). In the context of the current COVID-19 pandemic, by the time of writing this paper, three works have proposed the use of Deep Learning (DL) models for computer-assisted analysis of COVID-19 LUS imaging [32–34]. In this sense, in this work we evaluate and compare the performance of several deep-learning techniques for the identification of COVID-19 infections from lung ultrasound imagery. Our main contributions can be summarized as follows:

- We propose, implement, and evaluate the use of InceptionV3-, ResNet50-, VGG19-, and Xception-based deep learning (DL) models for COVID-19 screening in LUS imaging.
- We show that these DL-based models improve the COVID-19, pneumonia, and healthy classification performance on LUS imaging compared with the state-of-the-art classifiers.
- The proposed models are capable of providing the basis for further development of a LUS imaging-based COVID-19 computer-assisted screening tool.
- The provided results show that LUS is a viable alternative for development of computer-assisted COVID-19 screening tools based on medical imaging in scenarios where screening based on CT or X-ray is not readily available.
- Our work contributes in bringing the focus on DL techniques for computer-assisted analysis of lung pathologies based on LUS imaging, a research area that has not been widely explored and that has different advantages over CT or X-ray imaging for deploying e-Health applications.

Our motivation is based on the previously mentioned advantages offered by LUS like costs, availability, portability, etc., which are particularly relevant for developing countries and rural settings. In the next subsections we further discuss the use of LUS for COVID-19 screening and related literature on computer-assisted analysis of LUS relevant to this work.

### Motivation of LUS utilization for COVID-19 screening

In the last few years, Lung Ultrasound (LUS) imaging has been proposed as an alternative to the use of CT or X-ray for screening and follow-up of lung diseases [19–23]. For instance, it has been suggested that lung visualization through ultrasound imaging effectively replaces physical auscultation with stethoscopes [35]. Moreover, when used correctly, LUS imaging could even help to reduce infections between patients and medical staff [35].

Recent medical correspondence has pointed out the advantages of using LUS imaging as a tool for early diagnosis and follow-up of COVID-19 patients [36–41]. Some works highlight the benefits of using LUS in the context of the COVID-19 pandemic, especially considering its portability, accessibility, no radiation, ease of disinfection (e.g., using disposable caps of the ultrasound probes), and low cost [37, 38].

A short review of LUS findings in COVID-19 patients is presented in [36]. Some of these findings are consistent with CT results, including multiple fused bilateral B lines, subpleural pulmonary consolidations, irregular pleural line, and poor blood flow. An important finding reported by Fiala [36] is that subpleural lesions in COVID-19 patients differ signficantly from pulmonary diseases, including bacterial pneumonia, tuberculosis, and cardiogenic pulmonary edema, among others. Based on these observations, the author suggests adopting lung ultrasound for early detection of pulmonary alterations as a triage tool, particularly in environments with limited resources [36]. Additionally, other authors [38–40] reported that LUS imaging findings of COVID-19 progression in diagnosed patients are related to the observed patterns on CT images. Particularly, ground-glass opacity, consolidation shadow, and thickened pleura observed in CT images had special manifestations in LUS images as B-lines, consolidations, irregular or fragmented pleural line, pleural effusion, and absence of lung sliding. Furthermore, Soldati *et al*. [41] suggested that LUS findings in superficial pulmonary tissue are correlated with histopathological findings revealed in CT scanning.

Based on the previous discussion, it can be asserted that LUS imaging is a promising option for the screening, diagnosis, and follow-up of pulmonary diseases. Thus, over the past decade, interest in developing computational tools for computer-assisted analysis of LUS imaging has increased [24–30]. A brief description of the results reported in these works will be provided in the next section. We will first review works related to the identification of general lung conditions by means of computer-assisted analysis of LUS imaging. We will then describe previous work on computer-assisted screening of COVID-19.

### Related work on computer-assisted analysis of LUS

Earliest studies involving computer-assisted LUS imaging analysis were based on image processing techniques [42–44]. These works were aimed at segmentation (identification) of artifacts in LUS images. Most of the identified artifacts are the so-called B-lines, which are associated with the disease presence. Subsequent works applied the typical training-testing approach of classical Machine Learning algorithms (ML) and Deep Learning (DL) networks [24–31]. The study presented in [24–26] focuses on identifying pneumonia in children by analyzing brightness patterns in LUS frames. Authors [27–29] proposed pre-trained Convolutional Neural Networks (CNNs) to detect pulmonary conditions such as pleural effusion, consolidation, and pneumothorax utilizing images obtained from swine models. Their work is based on detecting the presence of lung sliding and quantification of A- and B-lines. In addition [31], proposes an automated quantification of B-lines in LUS frames using CNN. Meanwhile, a weakly-supervised strategy (i.e., needs only frame-level annotation) for localization of B-lines by means of Class Activation Maps was introduced in [30]. The previously mentioned studies are aimed at detecting artifacts in LUS frames to aid in the identification of different lung pathologies.

Taking into account the strong correlation between LUS and CT imaging findings in COVID-19 patients and the advantages of LUS equipment [38–40], it makes sense to develop tools for COVID-19 screening and diagnosis by means of computer-assisted analysis of LUS imaging. To the best of our knowledge, at the time of writing this paper, only three works have proposed the use of DL models for computer-assisted analysis of COVID-19 LUS imaging [32–34]. In [32], the authors addressed COVID-19 disease severity prediction considering the disease scoring scheme proposed in [45]. The deep learning architecture introduced in [34] produces a disease severity score from LUS imaging with the aim of assisting practitioners in the diagnosis of lung pathologies related to COVID-19. The work, first presented in [33] and extended in [34], aims to classify LUS frames obtained from healthy, bacterial pneumonia, and COVID-19 patients. These works introduce and use the Point-Of-Care Ultrasound (POCUS) dataset of LUS imaging. The POCUS dataset was collected by the authors of [33, 34] from different sources and was made publicly available in their GitHub repository [46]. The proposed model consists of a modified convolutional VGG16 network, called POCOVID-net, which provided an accuracy of 89% and balanced accuracy of 82% [33]. In the extended work [34], the POCUS database was enlarged, and four new models were evaluated: a NasNetMobile architecture; a VGG network combined with class activation maps (compute class-specific heatmaps); and two VGG-segment models using a version of segmentation ensemble introduced in [32]. In this work, the POCOVID-net model achieved the best 5-fold cross-validation results (accuracy and balanced accuracy of 87%) compared to other models.

Considering the results reported in [33, 34], there is still room for improvement in COVID-19 screening by means of computer-assisted analysis of LUS imaging based on artificial intelligence techniques.

## Methods

### Artifacts and related diseases in LUS

**Fig 1:**
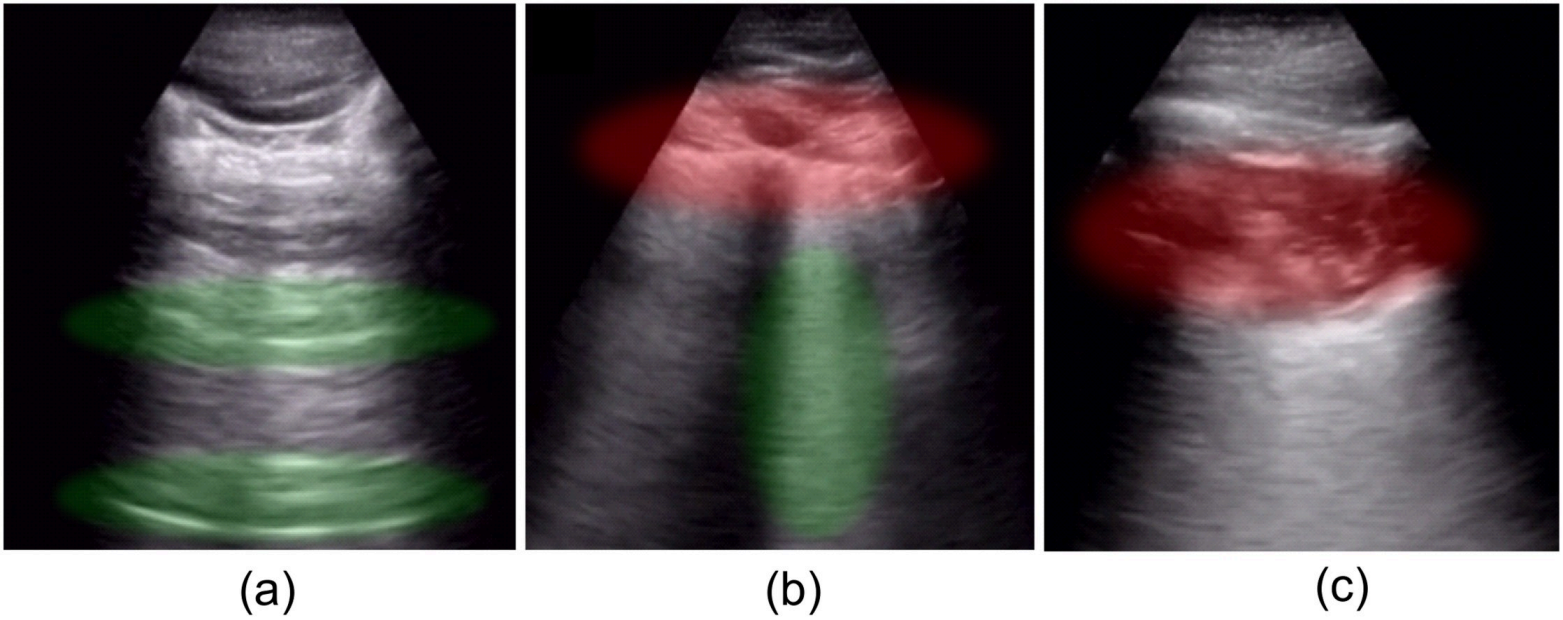
Artifacts. (a) normal lung with a regular pleural line, A-lines (green), and lung sliding; (b) infected lung with pleural line broken (red) and fused B-lines (green), (c) subpleural consolidation. Images were taken from the POCUS database [46].

Lung ultrasound imagery is generated from the relative amounts of air and fluid in lungs according to the physical phenomenon of acoustic impedance: a measure of particle resistance to mechanical vibrations in a medium [21]. The resistance increases proportionally to the density of the medium and propagation velocity of ultrasound in that medium. At a basic level, an ultrasound probe transmits high-frequency sound signals and listens to the echo caused by the reflection of such signals when they reach a boundary between tissues, known as interface. Ultrasound waves propagate across different tissues (mediums) in the organism. When ultrasound waves cross different interfaces, they experience different physical phenomena like attenuation, reflection, and refraction. In the examination of patients, these effects produce the so-called artifacts. These are shapes visualized in the echographic image generated from intensity and trajectory alterations of the beam when crossing structures with different acoustic properties (i.e., echogenicity). Among these artifacts, the main ones are [47]:

- **Pleural line**. A thin and echogenic line is seen just at the bottom of the intercostal musculature between ribs. A normal pleural line measures 0.2 to 0.3 mm.
- **A-lines**. These are a form of reverberation artifact produced by the reflection of the pleura. A-lines can be spotted as parallel lines to the pleural sliding (see Fig 1a), appearing uniformly spaced and descending to the bottom of the image. These lines allow us to recognize normally ventilated lungs, although they may also be present in pneumothorax.
- **B-lines**. Another form of reverberation artifact is seen as bright lines (also called comet-tails or comets) extending from the pleural line to the bottom of the image (see Fig 1b), obliterating A-lines. They move synchronously with respiration, and healthy lungs may show up to three lines per window/intercostal space.
- **Lung sliding**. Is described as a shimmering appearance of the pleura, that seems to slide back and forth as the patient breathes.

**Fig 2:**
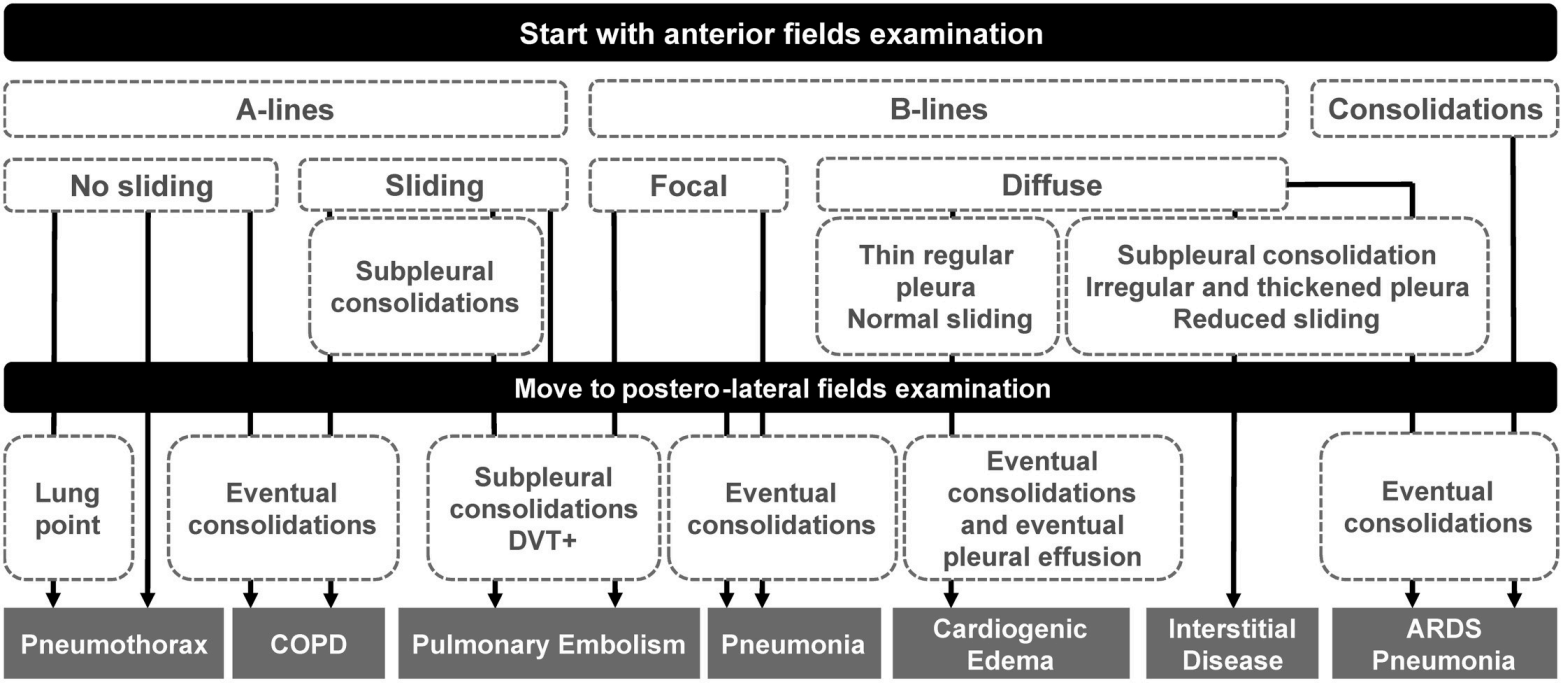
Flowchart for systematic diagnosis of lung diseases by means of LUS. (DVT—Deep Venous Thrombosis).

The appearance of different artifacts on LUS is related to particular diseases or lung conditions. Fig 2 shows a flowchart for systematic diagnosis of lung diseases by means of LUS proposed by [23]. The combination of the pleural line, A-lines, and bilateral lung sliding indicates regular lung aeration (see Fig 1a). However, the presence of these artifacts in LUS images is not exclusive to healthy lung conditions. For example, some of these artifacts, in addition to other patterns, can be observed in patients with Chronic Obstructive Pulmonary Disease (COPD) [22, 23]. Therefore, it is necessary to carefully analyze which artifacts appear in the LUS image altogether with its frequency (e.g., the number of B-lines), length, separation, morphology, etc., for the diagnosis of different lung conditions.

The appearance of B-lines (see Fig 1b) in LUS often indicate the presence of fibrous tissue, inflammatory cells, or extravascular lung water. These features are typical of Acute Respiratory Distress Syndrome (ARDS), interstitial lung diseases, pneumonia, pulmonary edema, among others. It is important to mention that isolated B-lines could have no pathological significance (less than three). However, depending on the number and distance between them, its appearance may be related to a particular lung condition. In general, the shorter the distance between B-lines (or even coalesced B lines), the less lung aeration [19]. Pleural effusion refers to an abnormal accumulation of fluid and no air in the pleural cavity. Ultrasound not only detects the effusion but also provides information on its nature and indicates the area for thoracentesis (i.e., removing fluid or air from the pleural space) [20]. Pleural effusion is related to conditions such as pneumonia or pulmonary edema. In contrast to pleural effusion, pneumothorax echo sonograms contain only air and no fluid. Besides, lung points (the alternation of normal and abolished lung sliding) with exclusive A-lines are a strong indicator of pneumothorax diagnosis [23]. Lung consolidation (see Fig 1c) appears as a dense tissue structure and the presence of white points characterized by an inspiratory reinforcement [19]. This indicates a complete loss of lung aeration because air spaces are filled with neoplastic tissue or pleural effusion. Lung consolidations are detected in pneumonia, lung atelectasis, lung contusion, ARDS, among others [22].

Regarding COVID-19 LUS images, multiple B-lines patterns (focal, multifocal, confluent), pleural thickening with line irregularity, pleural effusion, and different consolidation patterns have been detected in patients with COVID-19 diagnosis [40]. The previously mentioned artifacts and patterns appearing in LUS images are identifiable by trained clinicians and can be used to diagnose different lung conditions. Thus, as mentioned in the Introduction, this has led to the proposal of several computer-assisted approaches based on different artificial intelligence techniques [24–31, 42–44] to aid in the screening, diagnosis and follow-up of patients with different lung conditions.

### Deep learning architectures considered in the evaluation

This work evaluates different DL-based architectures to classify COVID-19 in LUS frames. As mentioned in the Introduction, three works have proposed the use of DL models for computer-assisted analysis of COVID-19 LUS imaging [32–34]. In particular, [33, 34] propose a DL-based model called POCOVID-net, which is based on a convolutional VGG16 network. CNNs are capable of extracting more complex features with less effort, but at the cost of large volumes of data [ref]. Besides the VGG16 network, different CNNs have been used successfully for computer-assisted analysis of medical images [48–50], including: VGG16 and VGG19 [51], InceptionV3 [52], Xception [53], and ResNet50 [54].

### Deep-learning based models for LUS COVID-19 classification

This work evaluates various DL-based models to classify COVID-19 in LUS frames. Considering that lung ultrasound is a portable, easy to disinfect, low cost and non-invasive tool, our goal is to lay the foundations needed to develop a tool for computer-assisted analysis of LUS imaging that can be effectively used for screening of COVID-19 patients in places where other testing alternatives are not readily available. With this in mind, in the following discussion, the classification performance achievable with different CNN architectures and classical machine learning techniques is provided. To perform the evaluation we used the dataset collected by Born *et al*. [34], which is publicly available at the GitHub repository provided by the authors [46].

### Baseline model: POCOVID-net

In this work, we adopted the POCOVID-net architecture [33, 34] as our baseline model for performance comparison of COVID-19 identificacion from LUS imagery. The goal of POCOVID-net is to correctly classify LUS frames obtained from healthy, bacterial pneumonia, and COVID-19 patients. In this regard, an accuracy of 89% and balanced accuracy of 82% for POCOVID-net was reported in [33], whereas an accuracy and balanced accuracy of 87% was reported in the extended work [34].

The POCOVID-net architecture consists of the convolutional part of the VGG16 architecture, pre-trained on ImageNet [55]. A hidden layer of 64 neurons replaced the VGG16 fully connected layer with ReLU activation, batch normalization, and dropout of 0.5, followed by an output layer of three nodes with a softmax activation function. For the training stage, fine-tuning was carried out only on the last three layers of the model (freezing the above layers) using cross-entropy loss function and the Adam optimization model [56] with a learning rate of 0.0001. To prevent overfitting, data augmentation was realized, allowing transformations such as rotations (10 degrees), horizontal and vertical flips, and shifts (10%). POCOVID-net implementation is provided by their authors in the GitHub repository [46].

### Deep-learning based models

We compare different CNN architectures and the baseline POCOVID-net model. In particular, we used the following CNN architectures: VGG19 [51], InceptionV3 [52], Xception [53], and ResNet50 [54]. We replaced the VGG16 base model of POCOVID-net with one of these CNN networks, preserving the last fully connected layers (hidden layer, batch normalization, dropout, and output layer). The number of trainable layers varied according to each model. Table 1 shows CNN configurations, where “Cropped layer” refers to the last layer used from the original architecture, and “First trainable layer” refers to the first unfrozen layer of the network for fine-tuning. All architectures were implemented in Python using Keras library [57].

**Table 1:** CNN configurations considered for COVID-19 identification in LUS imagery.

CNN	Cropped layer	First trainable layer	Trainable weights	Total weights
**VGG16**	block5_pool	block5_conv3	2,39,2963	14,747,971
**VGG19**	block5_pool	block5_conv4	2,392,963	20,057,667
**InceptionV3**	mixed7	conv2d_62	1,771,011	9,418,147
**Xception**	add_8	add_7	2,033,691	11,243,531
**ResNet50**	conv5_block3_out	conv5_block3_2_conv	3,547,011	23,719,299

Through numerous experiments, we found that the configurations shown in Table 1 provide very good network performance. To further improve the network performance, more extensive and time-consuming research should be done by varying the number of hidden network layers, the first trainable layer, batch size, epoch number, dropout, optimizer, learning rate, etc. To optimize the model hyper-parameters, it is additionally necessary to perform an exhaustive search such as gridSearchCV [58] or Hyperas [59]. Note that the number of hyper-parameter combinations can be quite large. A possible solution to reduce the number of combinations is to use estimates of hyper-parameters that have already been used to successfully solve similar problems. This will be performed in a future contribution.

### Dataset

**Fig 3:**
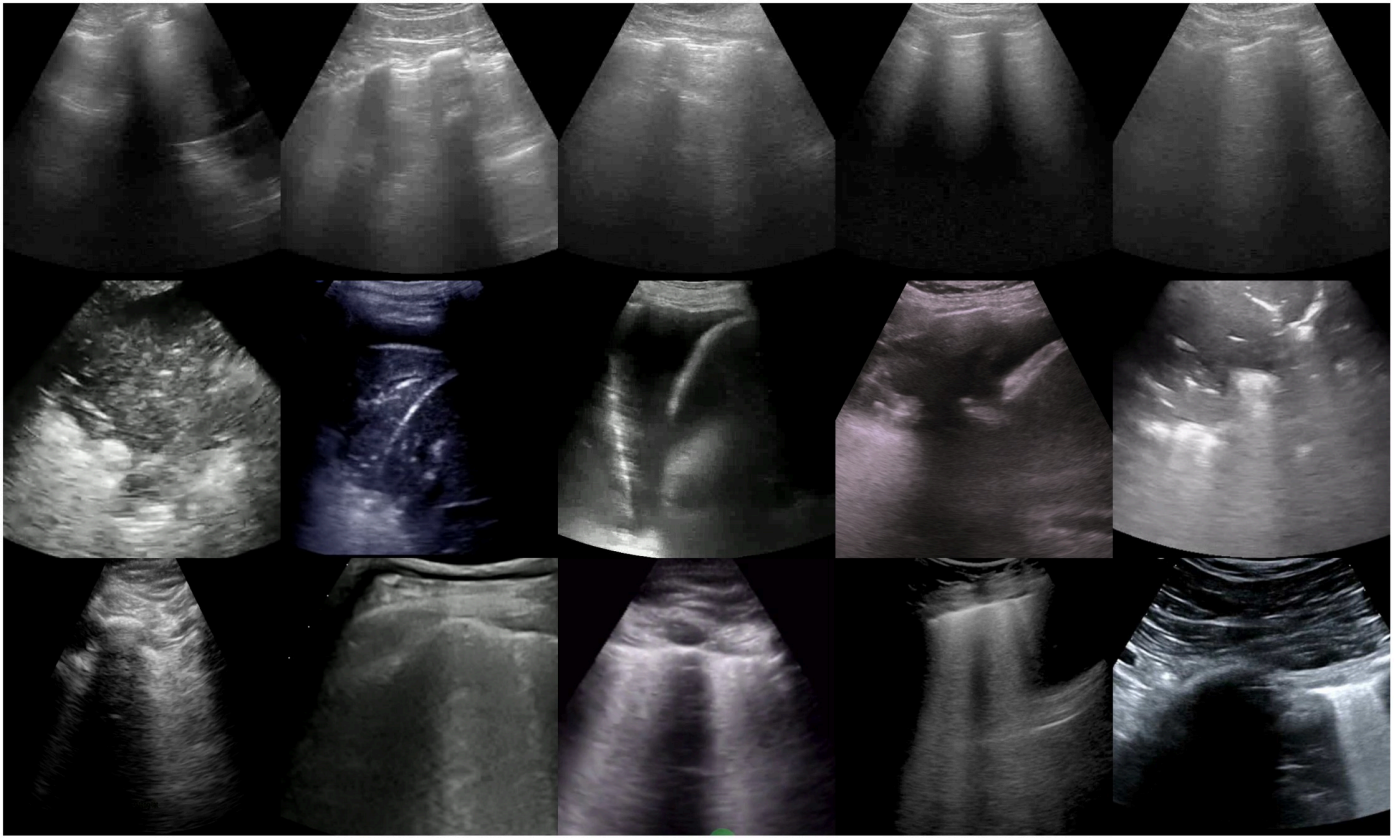
Examples of images obtained from LUS videos after pre-processing. Healthy (first row), pneumonia (second row), and COVID-19 (third row) LUS image classes.

For all experiments reported in this paper, we used the POCUS dataset collected by Born *et al*. [34], which is publicly available on the GitHub repository provided by the authors [46]. Fig 3 shows a few examples including healthy, pneumonia, and COVID-19 images. The POCUS dataset includes 202 videos and 59 images recorded with either convex or linear probes comprising samples of 216 patients with COVID-19, bacterial and (non-COVID19) viral pneumonia, and healthy controls. The POCUS dataset was recollected from 41 different sources, including clinical data donated from hospitals or academic ultrasound course instructors, LUS recordings published in other scientific literature, community platforms, open medical repositories, and health-tech companies *et al*. [34]. Thus, the videos have different length and frame rate (160±144 frames, 2510Hz). Furthermore, due to the variety of data sources, not all videos include patient metadata. Only 42% of data include age (average age is 41.3 years) and gender (57% were male) information, and 30% include symptoms descriptions. Nevertheless, all samples of the POCUS database were reviewed and approved by two medical experts, and the COVID-19 diagnosis was usually made by RT-PCR.

For our experiments, we employed 185 convex videos and 58 single images. The videos and images were pre-processed as described in [34] by using the tools available at [54], hence:

- LUS videos were split into images at a frame rate of 3 Hz.
- Images were cropped to a quadratic window.
- Finally, images were resized to 224 × 224 pixels.

After video sampling and image pre-processing, our data set comprised 3326 images (including the single images) of which 1283 correspond to COVID-19, 731 correspond to bacterial pneumonia, and 1312 correspond to healthy labeled images. To prevent overfitting, data augmentation transformations such as rotations (10 degrees), horizontal and vertical flips, shifts (10%), and zoom (zoom range of 20%) were added.

In order to increase the robustness of the results, we carried out repeated 5-fold cross-validation [60]. For each 5-fold cross-validation, the dataset was randomly partitioned into five folds, with considerations that the folds are class balanced and disjoint (i.e., all frames sampled from the same video are assigned to only one fold). From the five folds, four of them were chosen and merged as the training set while the remaining fold was used as the validation set. Then, the 5-fold cross-validation was repeated 5 times modifying the folds division (5 × 5-fold cross-validation). Detailed per fold image information is included in S1 File. For each 5-fold cross-validation, all CNN architectures were fine-tuned using the corresponding training set under 30 epochs with a batch size of 32 (with early stopping enabled). Then, the resulting trained models were evaluated under different performance metrics utilizing the corresponding validation set. Detailed learning curves, confusion matrices, and ROC curves of models for each 5 × 5-fold cross-validation are included in supporting information (S2–S6 Files).

### Statistical analysis

Performance results obtained by the different architectures are presented by averaging a total of 25 independent runs of the repeated 5 × 5-fold cross-validation. These results are introduced using the most common utilized performance metrics for evaluating machine learning methods [61, 62]:

- Precision, Recall, and F1-score metrics for per class classification performance.
- Accuracy (ACC), Balanced Accuracy (BACC), and Area Under the Receiver Operating Characteristic Curve (AUC-ROC) metrics for overall method performance evaluation.

Both Precision and Recall metrics indicate how many model predictions are actually correct. The Precision metric considers false positives, whereas the Recall metric is associated with false negatives. Note that for COVID19 classification it is more important to reduce the number of false negatives (high Recall), since a false positive can be discarded in the second test. The F1-score represents the Harmonic mean between Precision and Recall metrics.

On the other hand, Accuracy and Balanced Accuracy (for imbalanced class data) indicate the number of correct predictions out of the total data samples, evaluating the overall method performance. Finally, the ROC curve represents a trade-off between true and false positive rates under different probability thresholds for the predictive model.

Additionally, the ANOVA test was applied to analyze the statistical difference in the overall performance of the evaluated models. However, normal distribution and variance homogeneity are assumed for the ANOVA test. This can not always be guaranteed for the performance analysis of machine learning algorithms [62, 63]. Thus, we also applied the non-parametric Friedman test [62, 63]. Then, post-hoc analysis was carried out as suggested in [64] using the Wilcoxon signed-rank test with Holm’s step down correction [63, 65], for pair-wise comparison between the evaluated classifiers. Detailed results are presented in suplemental information S7 File. This enabled us to further assess statistical significant differences in the performance results provided by the different CNNs evaluated in this work. All average results are presented with a 95% confidence interval (95% C.I.).

## Results

For each repeated 5 × 5-fold cross-validation experiment, CNN-based networks were trained and validated using different partitions of the dataset, producing a total of 25 learning curves (Loss/Accuracy vs Epoch number), matrices confusion, and ROC curves for each model. Detailed 5 × 5-fold cross-validation results are included in supporting information (S2–S6 Files).

**Fig 4:**
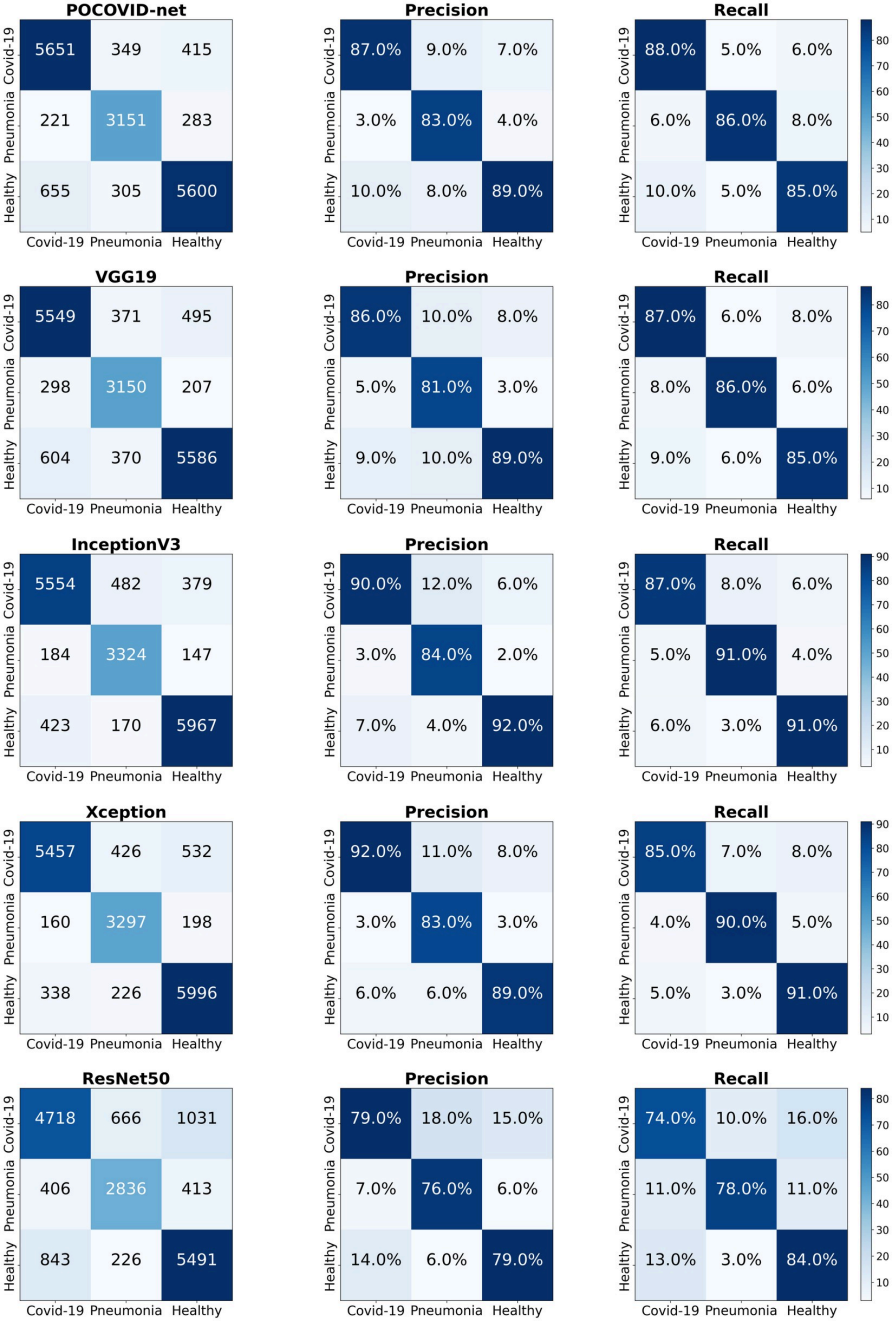
5 × 5-fold cross-validation results: Confusion matrices.

Table 2 presents the mean 5 × 5-fold cross-validation results obtained with the different CNN-based models (95% C.I.) considered in our experiment. We can observe that the InceptionV3-based model achieved the highest ACC, BACC, and AUC-ROC mean values of 89.1%, 89.3%, and 97% respectively. In contrast, the ResNet50-based model provided the worst ACC, BACC, and AUC-ROC mean metrics (78.3%, 78.1%, and 90%, respectively). In terms of Precision, Recall, and F1-score, the InceptionV3-based model again provided the best mean metric values (see also Fig 4). In contrast, the ResNet50-based model yielded the worst mean precision, recall and F1-score values (see Fig 4). Note as well that, in general, InceptionV3 and Xception-based models provide higher ACC, BACC, AUC-ROC, Precision, Recall, and F1-score mean values than the POCOVID-net architecture.

**Table 2:** Overall performance results of the evaluated models (95% C.I.).

Model	Classes	Precision	Recall	F1-score
**InceptionV3** ACC 89.1(±2.3) BACC 89.3(±2.2) AUC-ROC 97.1(±1.0)	COVID-19	90.1(±3.1)	86.4(±3.6)	88.0(±3.0)
	Pneumonia	84.2(±3.7)	90.8(±2.5)	87.1(±2.5)
	Healthy	91.8(±2.1)	90.7(±2.6)	91.1(±2.1)
**Xception** ACC 88.6(±2.3) BACC 88.7(±2.3) AUC-ROC 97.0(±0.9)	COVID-19	91.4(±2.7)	85.1(±3.5)	88.0(±2.8)
	Pneumonia	84.1(±3.8)	90.0(±3.3)	86.6(±3.0)
	Healthy	89.0(±2.3)	91.1(±2.5)	89.9(±2.1)
**POCOVID-net** ACC 86.5(±1.8) BACC 86.3(±1.8) AUC-ROC 95.4(±0.9)	COVID-19	86.9(±2.8)	87.9(±3.0)	87.2(±2.3)
	Pneumonia	83.7(±3.4)	85.9(±3.9)	84.3(±2.5)
	Healthy	88.9(±2.0)	85.1(±2.6)	86.8(±1.8)
**VGG19** ACC 85.8(±2.0) BACC 85.8(±2.0) AUC 95.2(±1.0)	COVID-19	86.2(±3.2)	86.6(±3.1)	86.2(±2.7)
	Pneumonia	82.4(±4.0)	85.9(±3.8)	83.5(±2.6)
	Healthy	88.8(±2.3)	85.0(±2.7)	86.6(±1.9)
**ResNet50** ACC 78.3(±2.0) BACC 78.1(±2.1) AUC-ROC 90.2(±1.3)	COVID-19	80.0(±4.1)	73.5(±5.5)	75.6(±3.2)
	Pneumonia	77.8(±4.9)	77.4(±4.9)	76.6(±3.3)
	Healthy	79.6(±2.8)	83.5(±2.9)	81.1(±1.7)

**Fig 5:**
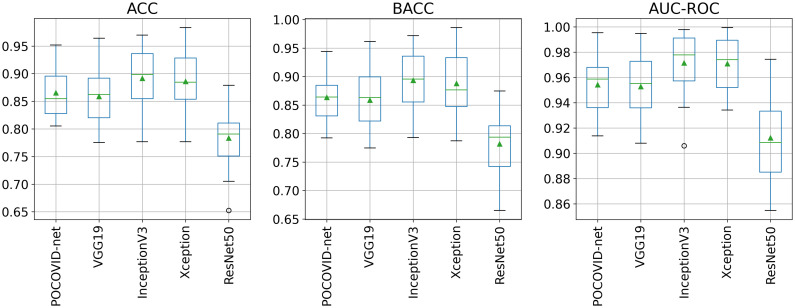
5 × 5-fold cross-validation results: ACC, BACC, and AUC-ROC ACC, BACC, and AUC-ROC scores boxplots. Box extends from the Q1 to Q3 quartile values of the data, with a line at the median and a triangle at the mean.

Fig 5 shows ACC, BACC, and AUC-ROC boxplots obtained from all the 5 × 5-fold cross-validation experiments. From Fig 5, we can readily see that the ResNet50-based model provided the worst performance. In contrast, InceptionV3 and Xception-based models seem to provide better ACC, BACC, and AUC-ROC performance compared to the other models.

**Fig 6:**
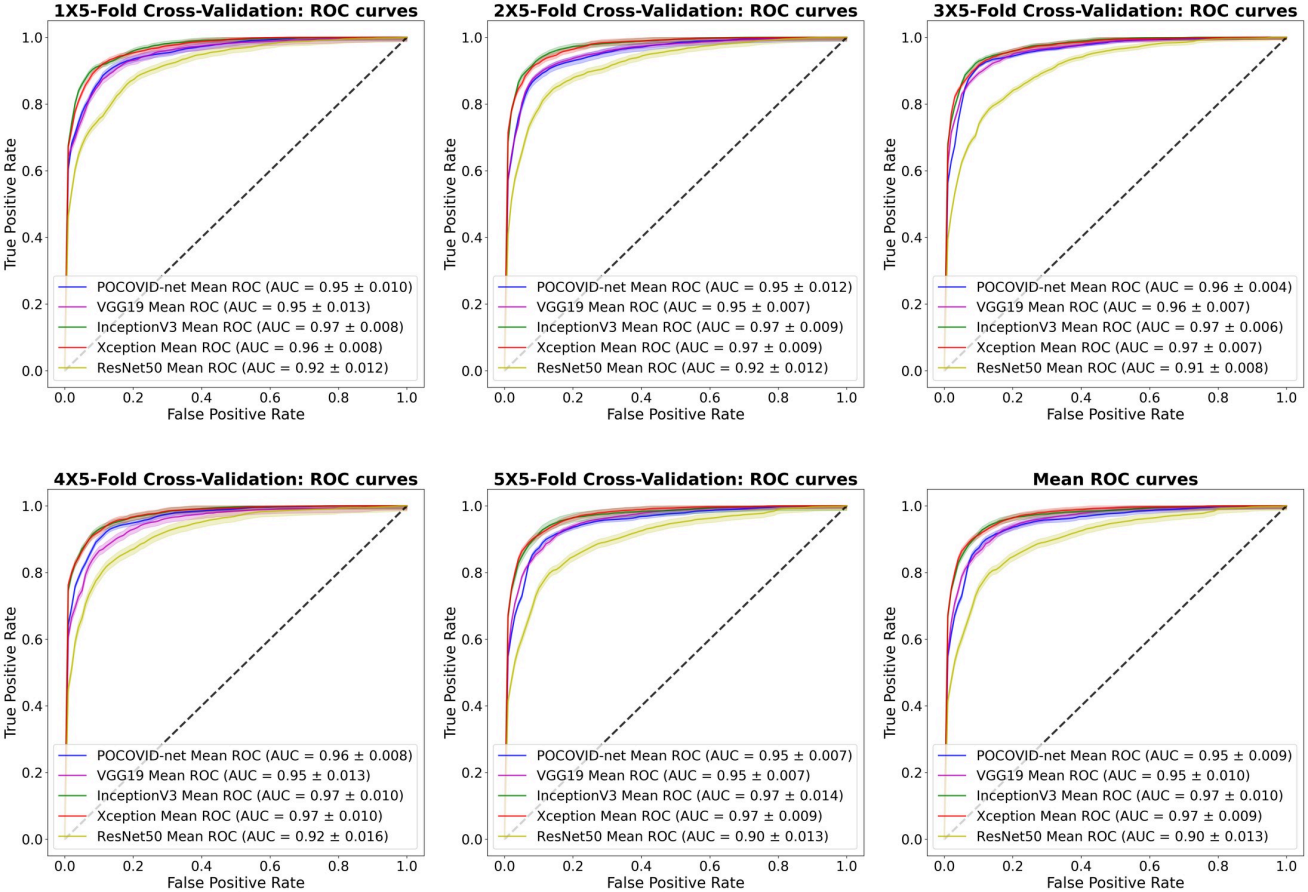
5 × 5-fold cross-validation results: Mean ROC curves and AUC scores (95% C.I.).

Finally, Fig 6 shows the ROC curves obtained from each repeated 5 × 5-fold cross-validation. The mean ROC curve is included as well in this figure. The InceptionV3 and Xception-based models provided the best performance, achieving a mean AUC-ROC of 97%. We can clearly observe that the ResNet50-based network provided the worst AUC-ROC for each experiment with a mean AUC-ROC of 90%.

To further analyze whether there is a statistically significant difference in the overall performance of the DL models, we applied the ANOVA and Friedman tests following [62, 63]. The ANOVA test was applied to the repeated 5 × 5-fold cross-validation results provided in S7 File, using the Python library scipy statsmodels [66]. The ANOVA test result showed statistically significant differences between the performances of the evaluated CNN-based models with ACC: *F* = 17.8, *P* < .001; BACC: *F* = 19.07, *P* < .001; and AUC: 23.02, *P* < .001.

Although the ANOVA test showed statistically significant differences, as explained in [62, 63], the Friedman omnibus test is better suited when comparing the performance provided by machine learning algorithms (of which DL is a subset). Thus, the Friedman test was also applied to the repeated 5 × 5-fold cross-validation results summarized in S7 File, using the Python library scipy statsmodels [66]. The Friedman test also shows statistically significant performance differences between models with *P* < .001 for all metrics.

**Fig 7:**
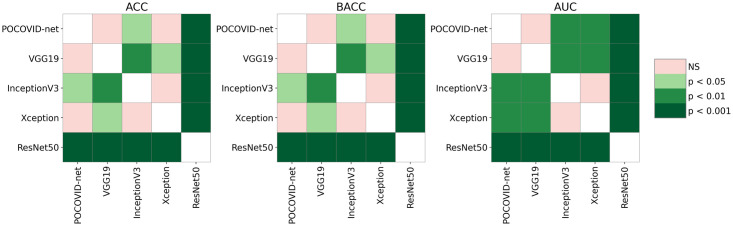
Post-hoc pairwise comparison using the Wilcoxon signed-rank test with Holm correction.

After applying the Friedman test, a post-hoc pairwise analysis was carried out using the Wilcoxon signed-rank test (as suggested in [64]) with Holm’s correction [58, 63, 65], using a significance level of *α* = 0.05. The results of the pairwise comparison are represented graphically in Fig 7. As we can observe in Fig 7, InceptionV3-based model had significantly higher mean ACC, BACC, and AUC than the baseline POCOVID-net (ACC: *P* = .03, BACC: *P* = .02, AUC: *P* = .007), VGG19 (ACC: *P* = .004, BACC: *P* = .002, AUC: *P* = .003), and Resnet50-based models(*P* < .001 for all metrics). No statistically significant difference was found between InceptionV3 and Xception-based models performances (*P* = 0.79).

To further study the performance of the DL models, two additional experiments were executed. The first one evaluates the CNN-based models considering only two classes: COVID-19 and pneumonia. The second one considers two classes as well, but this time one is COVID-19 and the other one is non-COVID-19 (which includes healthy and pneumonia images). The results obtained for the COVID-19 vs pneumonia experiment are presented in Table 3. For this experiment, the healthy images were excluded from the original partitions and then repeated 5 × 5-fold cross-validation was performed. Note from Table 3 that POCOVID-net achieved the best ACC and BACC performance closely followed by the InceptionV3-based model for this binary classification. Furthermore, both classifiers provided the best AUC-ROC performance of 97%.

**Table 3:** Overall COVID-19 vs pneumonia performance results (95% C.I.).

Model	Classes	Precision	Recall	F1-score
**POCOVID-net** ACC 94.1(±1.7) BACC 94.0(±1.7) AUC-ROC 98.0(±0.8)	COVID-19	96.3(±1.4)	94.5(±2.1)	95.3(±1.3)
	Pneumonia	90.1(±3.2)	93.5(±2.8)	91.9(±2.4)
**InceptionV3** ACC 93.4(±1.9) BACC 93.5(±1.7) AUC-ROC 98.0(±0.8)	COVID-19	96.5(±1.1)	93.1(±2.4)	94.6(±1.5)
	Pneumonia	88.9(±3.6)	94.0(±2.0)	91.2(±2.5)
**VGG19** ACC 93.0(±1.8) BACC 93.2(±1.8) AUC-ROC 97.3(±0.9)	COVID-19	96.2(±1.3)	92.7(±2.2)	94.3(±2.7)
	Pneumonia	88.5(±3.4)	93.6(±2.2)	90.8(±2.6)
**Xception** ACC 92.8(±2.3) BACC 93.4(±2.1) AUC-ROC 97.5(±1.0)	COVID-19	97.3(±1.1)	91.3(±2.9)	94.1(±2.0)
	Pneumonia	86.9(±4.0)	95.5(±1.8)	90.8(±2.9)
**ResNet50** ACC 86.3(±1.9) BACC 85.2(±2.5) AUC-ROC 93.4(±1.6)	COVID-19	89.9(±2.4)	88.8(±2.7)	89.1(±1.6)
	Pneumonia	82.3(±3.2)	81.5(±5.6)	80.8(±3.3)

The 5 × 5-fold cross-validation results obtained for the COVID-19 vs non-COVID-19 experiment are presented in Table 4. For this experiment, images from pneumonia and healthy classes were randomly selected to create the non-COVID-19 class. Note from Table 4 that the InceptionV3-based model achieved the best ACC and BACC performance closely followed by POCOVID-net and the Xception-based model for this binary classification. However both, POCOVID-net and the Xception-based model, provided the best AUC-ROC performance of 96% (closely followed by the AUC-ROC metric of 95% achieved by the InceptionV3-based model).

**Table 4:** Overall COVID-19 vs non-COVID-19 performance results (95% C.I.).

Model	Classes	Precision	Recall	F1-score
**InceptionV3** ACC 91.5(±2.1) BACC 91.3(±2.1) AUC-ROC 96.7(±1.3)	COVID-19	94.1(±2.1)	87.9(±3.3)	90.7(±2.4)
	non-COVID19	89.6(±2.5)	94.8(±1.8)	92.0(±1.9)
**POCOVID-net** ACC 90.5(±2.0) BACC 90.4(±2.0) AUC-ROC 95.6(±1.4)	COVID-19	92.5(±1.8)	87.4(±3.2)	89.8(±2.3)
	non-COVID19	89.2(±2.6)	93.4(±1.6)	91.1(±1.9)
**Xception** ACC 90.3(±2.3) BACC 90.1(±2.3) AUC-ROC 96.1(±1.3)	COVID-19	94.7(±1.8)	84.7(±4.0)	89.1(±2.7)
	non-COVID19	87.6(±3.0)	95.6(±1.5)	91.3(±2.0)
**VGG19** ACC 89.7(±2.3) BACC 89.7(±2.3) AUC-ROC 94.4(±1.7)	COVID-19	89.7(±3.1)	89.4(±3.1)	89.2(±2.4)
	non-COVID19	90.5(±2.5)	90.1(±3.3)	90.0(±2.3)
**ResNet50** ACC 82.8(±2.3) BACC 82.4(±2.2) AUC-ROC 88.9(±2.0)	COVID-19	84.7(±2.4)	78.2(±4.7)	80.8(±3.0)
	non-COVID19	82.1(±2.6)	86.7(±2.7)	84.0(±1.6)

Note how the performance of the CNN-models for the COVID-19 vs non-COVID-19 experiment dropped compared to the COVID-19 vs pneumonia experiment. Upon visual inspection, we conjecture that this may be caused by the similarity between COVID-19 and healthy LUS images. Deep analysis of this observation is out of the scope of the current paper, and therefore will be addressed in future work.

For both experiments, we also applied the Friedamn test followed by post-hoc analysis using the Wilcoxon signed-rank test with the Holm’s step-down correction. In both cases, the Friedman test showed statistically significant differences. However, when performing the pairwise post-hoc test, only statistically significant differences were found between the ResNet50-based network and the remain CNN-based models (*P* < 0.01).

## Discussion

### Principal results

In this work, we evaluated the use of different deep learning architectures to classify LUS frames acquired from COVID-19, bacterial pneumonia, and healthy patients. The modified VGG19, InceptionV3, Xception, and ResNet50 networks were trained, evaluated, and compared with the baseline POCOVID-net model on the POCUS dataset under a 5 × 5-fold cross-validation scheme. After carrying out the statistical analysis of the evaluated DL-based models (summarized in Table 2 and the corresponding discussion), we found out that the fine-tuned InceptionV3-based network achieved a statistically significantly higher mean ACC of 89.1%, BACC of 89.3% and AUC-ROC of 97% than the baseline POCOVID-net, VGG19-, and ResNet50-based models. No statistically significant difference was found between InceptionV3- and Xception-based models performances. Thus, it can be concluded that the InceptionV3-based network provides the most promising predictive results of all AI-based techniques evaluated in this work. Furthermore, it could be expected that with more data, the performance of this model will continue to increase. In this regard, we consider that the Xception-based model is also worthy of consideration for further evaluation as more data becomes available.

### Limitations

The experiments were carried out on an open access dataset of LUS images, including COVID-19, bacterial pneumonia, and examples from healthy people. A larger dataset would be required to develop a fine-tuned version of the evaluated techniques. To the best of our knowledge, this is a limitation of most modern medical imaging machine learning tools. In the future, a classification tool trained on a huge dataset of hundreds or even thousands of patients (rather than a few images taken from a few patients) will be needed to validate the generalization of any automated diagnostic assistant tool.

### Comparison with prior work

While previous work focused on the use of a VGG16 convolutional architecture for the classification of LUS frames acquired from COVID-19, bacterial pneumonia, and healthy patients, the present work took a step further. Particularly we studied the use of other CNN configurations to address this task.

### Conclusions

In the context of the current COVID-19 pandemic, where health services are often saturated, the use of automated image diagnosing tools could importantly help to alleviate the burden of health systems with a limited number of specialized clinicians. In this paper, we do not compete with AI-based solutions previously proposed for computer-assisted COVID-19 screening based on CT or X-ray imaging. Instead, we seek to lay the foundations to develop an AI-based COVID-19 screening tool that uses LUS imaging as an alternative when there is limited or no access to CT or X-ray equipment. In this sense, the advantages offered by LUS (e.g., portability, cost, ease of disinfection, etc.) combined with the feasibility of readily implementing and deploying already trained AI-based solutions in a wide variety of portable devices (e.g., laptops, smartphones, etc.), has the potential of providing an accessible and mobile COVID-19 screening tool for medical staff. This fact becomes crucial in rural areas or developing countries, where wide access to ultrasound devices might be more feasible because of its portability and low-cost features compared to medical imaging devices like CT and X-ray. In addition to the benefits for screening and diagnosis, trained algorithms applied to the recognition of LUS artifacts would allow to follow the progress of COVID-19 in diagnosed patients, assisting decisions in medical treatments. Thus, besides improving the performance of the classifiers as more LUS images become available, future research will address the use of CNN-models for computer-assisted analysis of LUS imaging with the aim of helping with the screening and follow-up of other lung related pathologies.

## Supporting information

S1 File Data partitioning. Number of images utilized in training and validation sets for each of the 5-fold cross-validation experiments.
(XLSX)

S2 File POCOVID-net experimental results. Detailed learning curves, confusion matrices, and AUC-ROC curves of each repeated 5 × 5-fold cross-validation experiments.
(PDF)

S3 File VGG19-net experimental results. Detailed learning curves, confusion matrices, and AUC-ROC curves of each repeated 5 × 5-fold cross-validation experiments.
(PDF)

S4 File InceptionV3 experimental results. Detailed learning curves, confusion matrices, and AUC-ROC curves of each repeated 5 × 5-fold cross-validation experiments.
(PDF)

S5 File Xception experimental results. Detailed learning curves, confusion matrices, and AUC-ROC curves of each repeated 5 × 5-fold cross-validation experiments.
(PDF)

S6 File ResNet50 experimental results. Detailed learning curves, confusion matrices, and AUC-ROC curves of each repeated 5 × 5-fold cross-validation experiments.
(PDF)

S7 File Statistical analysis. Detailed performance results, ANOVA and Friedman tests, and Wilcoxon test with Holm’s correction for three- and two-class experiments.
(XLSX)

## Abbreviations

ACC: Accuracy
AI: Artificial Intelligence
ARDS: Acute Respiratory Distress Syndrome
AUC-ROC: Area Under the Receiver Operating Characteristic Curve
BACC: Balanced Accuracy
CI: Confidence Interval
CNN: Convolutional Neural Network
CT: Computed Tomography
DL: Deep Learning
LUS: Lung Ultrasound
ML: Machine Learning
POCUS: Point-Of-Care Ultrasound
ROI: Region Of Interest
WHO: World Health Organization
