# Supplementary material for: Deep-learning based detection of COVID-19 using lung ultrasound imagery
Source: PLoS One. 2021 Aug 13;16(8):e0255886. doi: 10.1371/journal.pone.0255886 (PMC8363024; doi:10.1371/journal.pone.0255886)
Supplement: S3 File — Detailed learning curves, confusion matrices, and AUC-ROC curves of each repeated 5 × 5-fold cross-validation experiments. (PDF) [file pone.0255886.s003.pdf]

VGG19 based model

1×5 −Fold Cross-Validation

Learning curves

Fold1

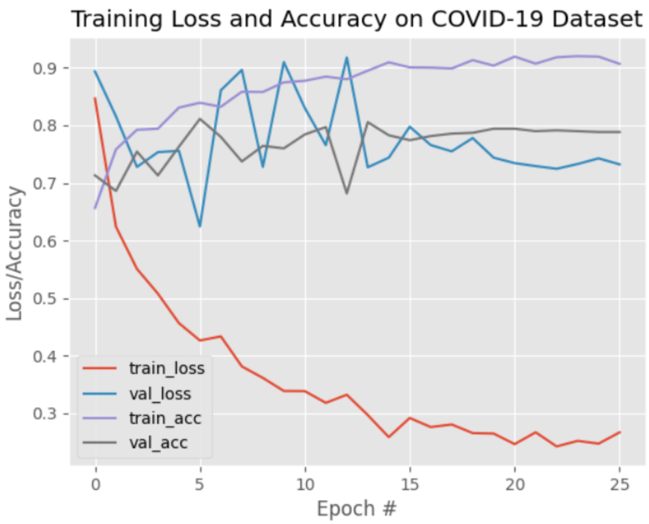

Fold2

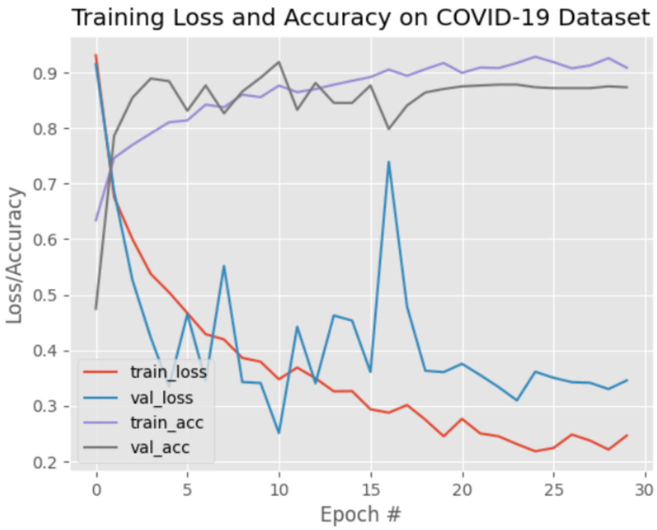

Fold3

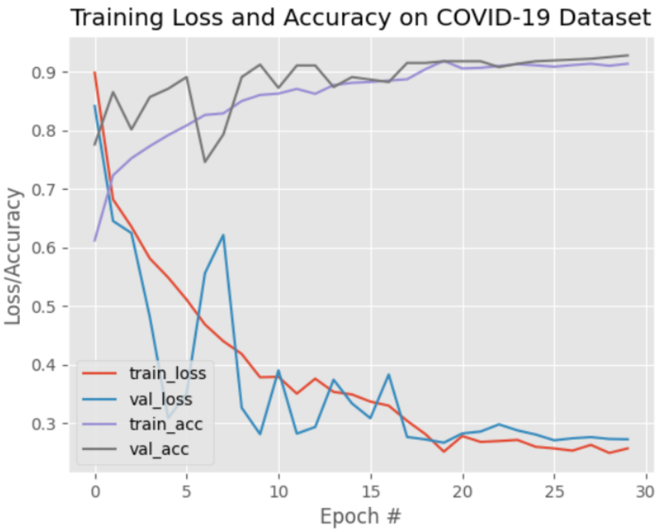

Fold4

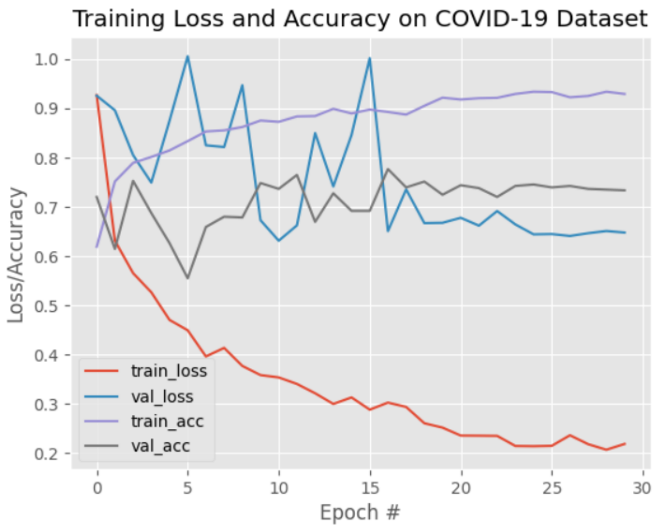

Fold5

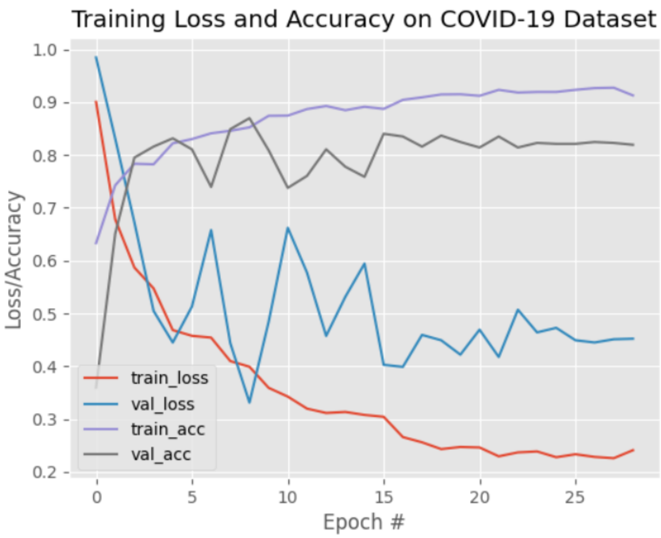

Confusion matrices

Fold1

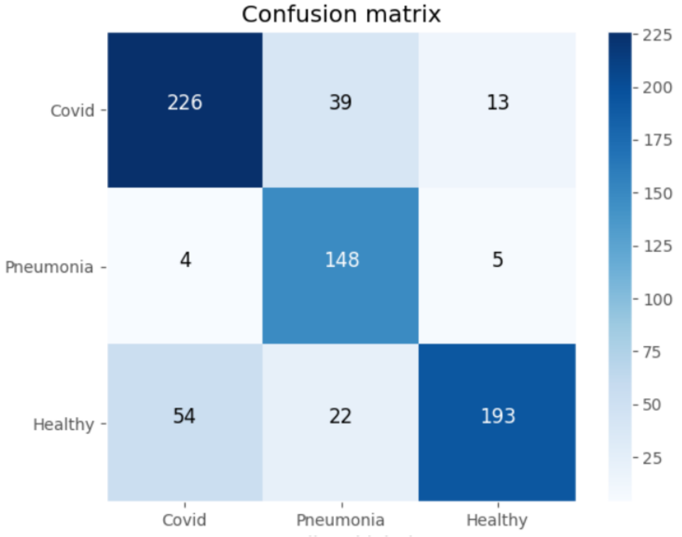

Fold2

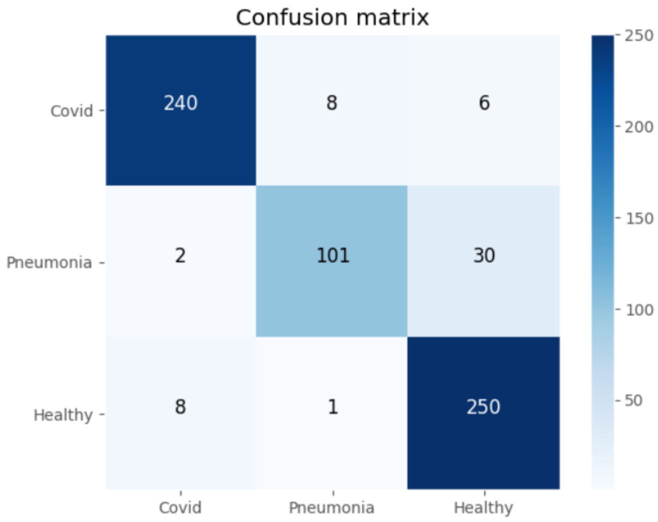

Fold3

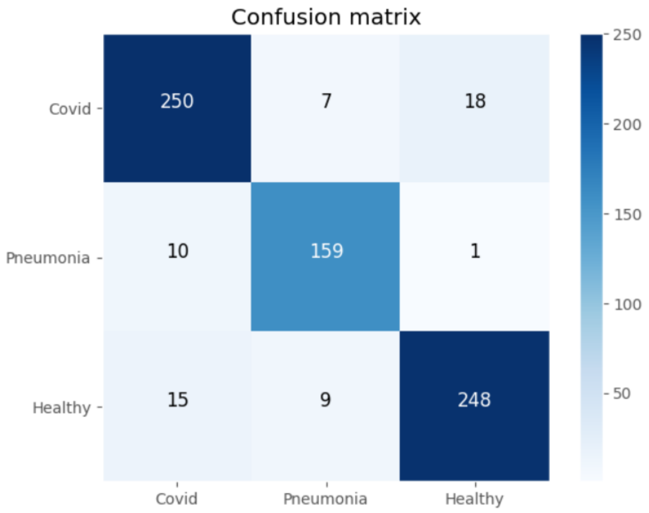

Fold4

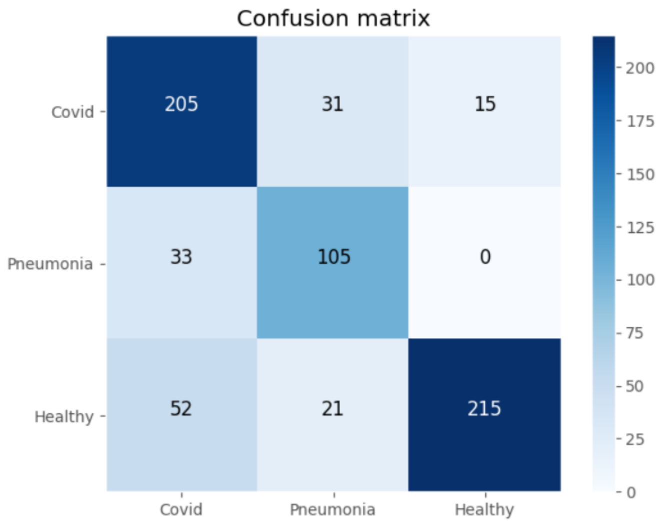

Fold5

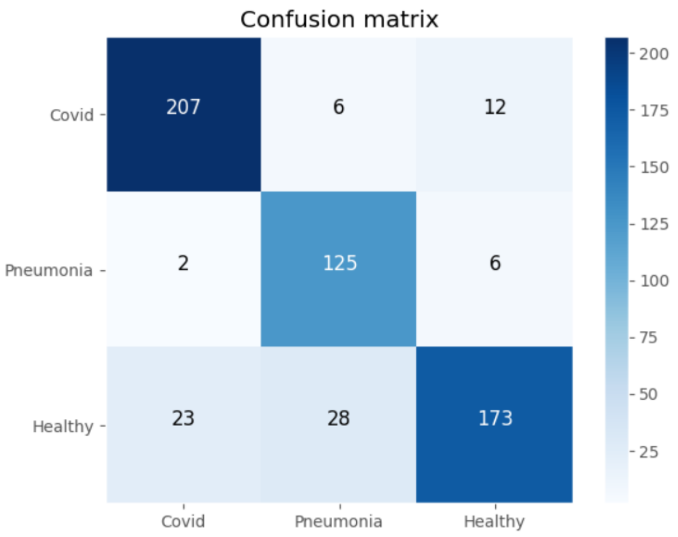

AUC-ROC curves

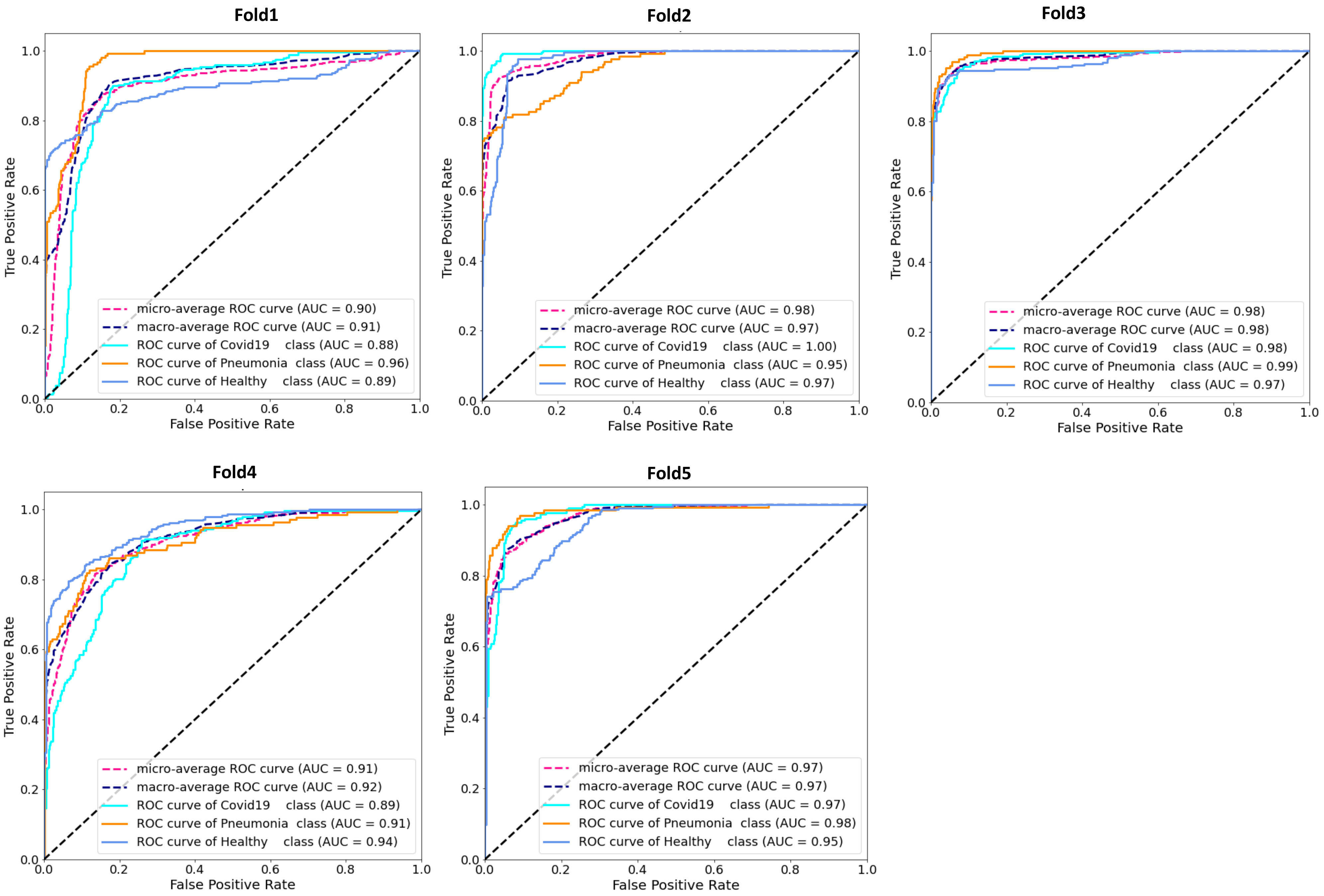

2×5 –Fold Cross-Validation

Learning curves

Fold1

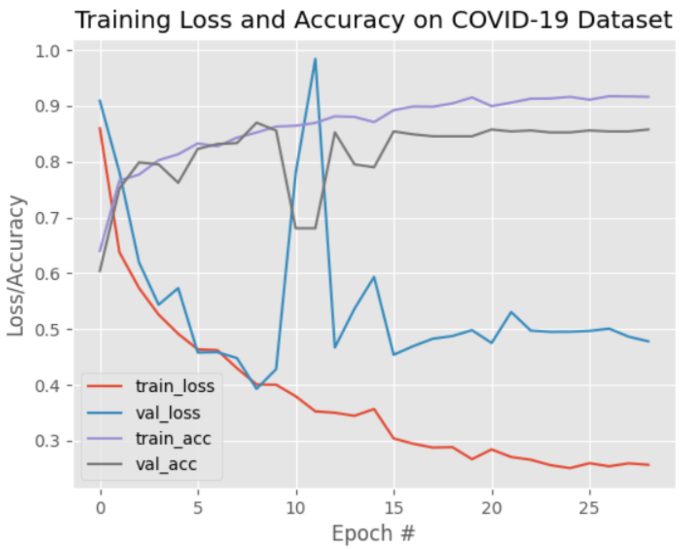

Fold2

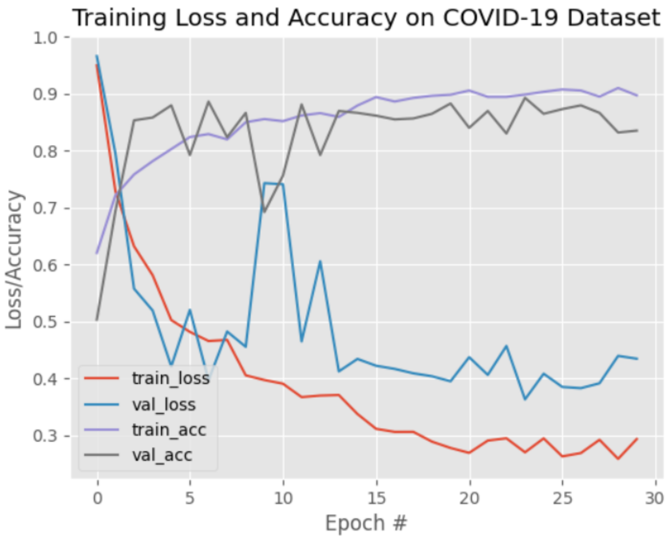

Fold3

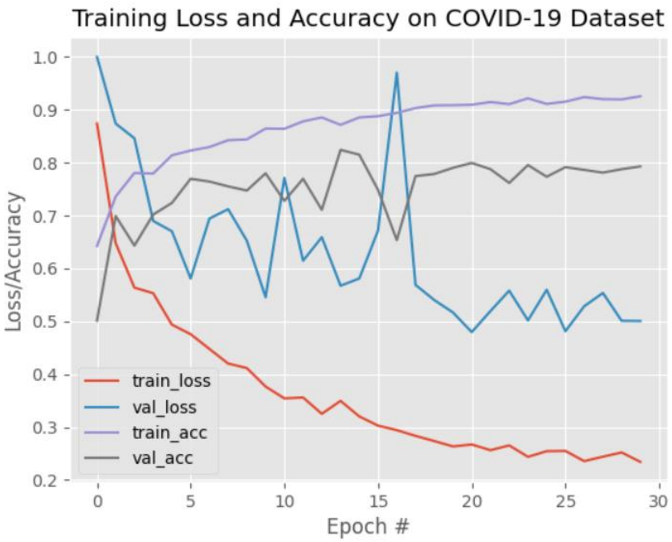

Fold4

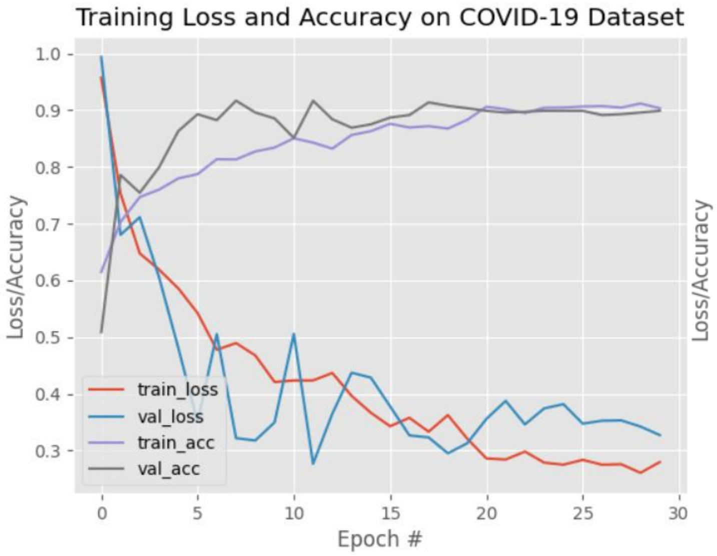

Fold5

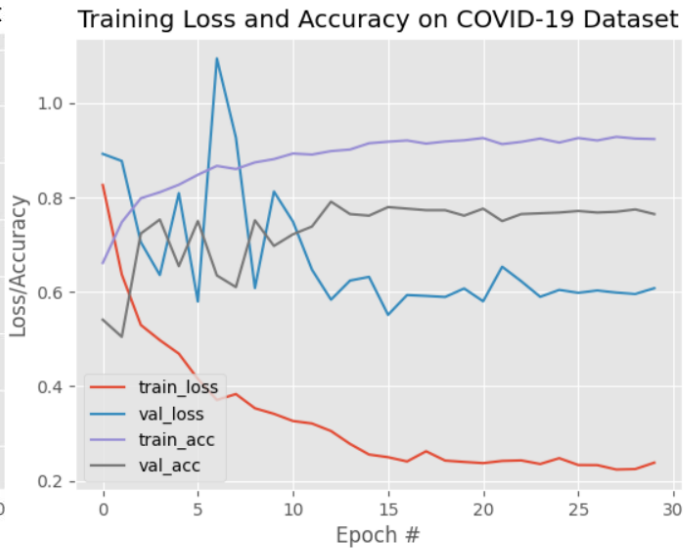

Confusion matrices

Fold1

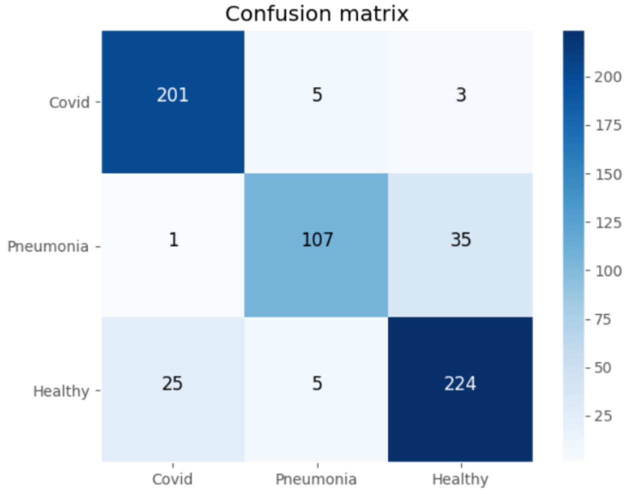

Fold2

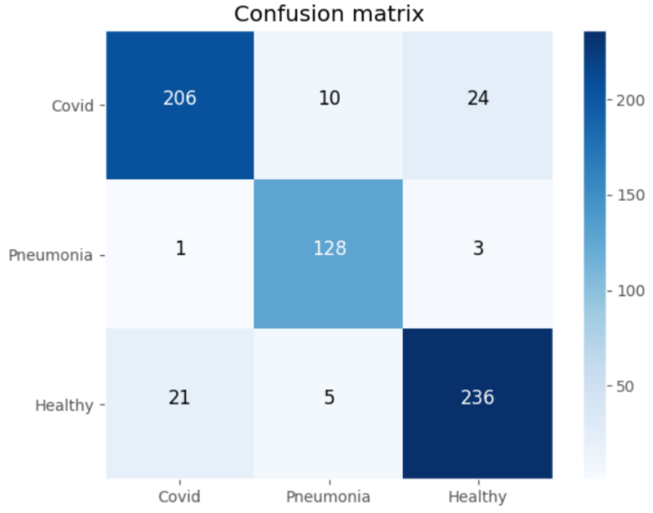

Fold3

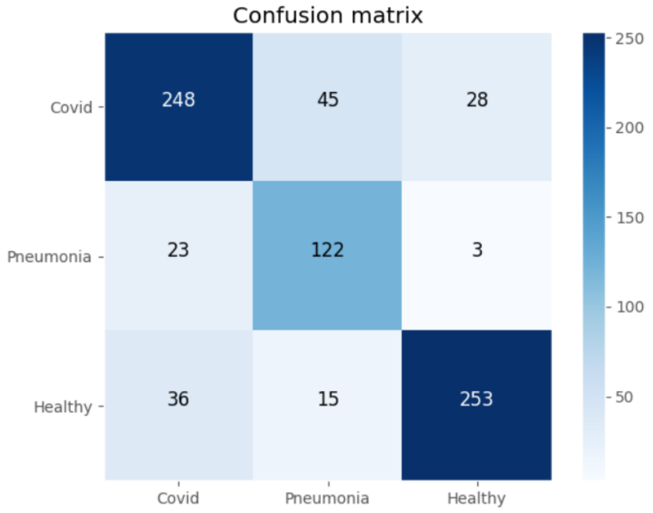

Fold4

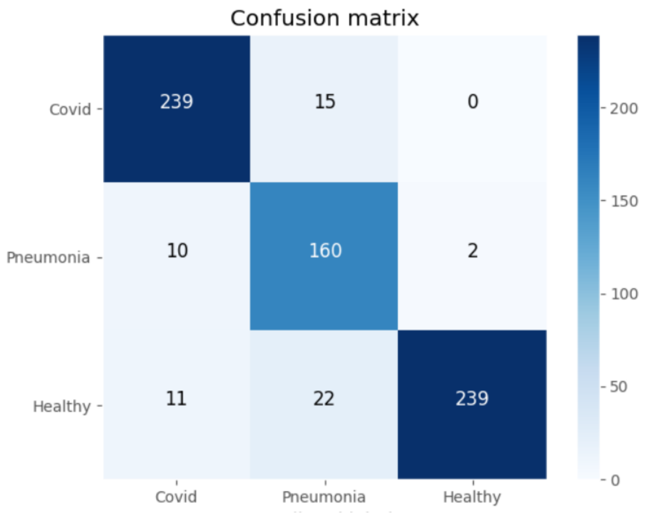

Fold5

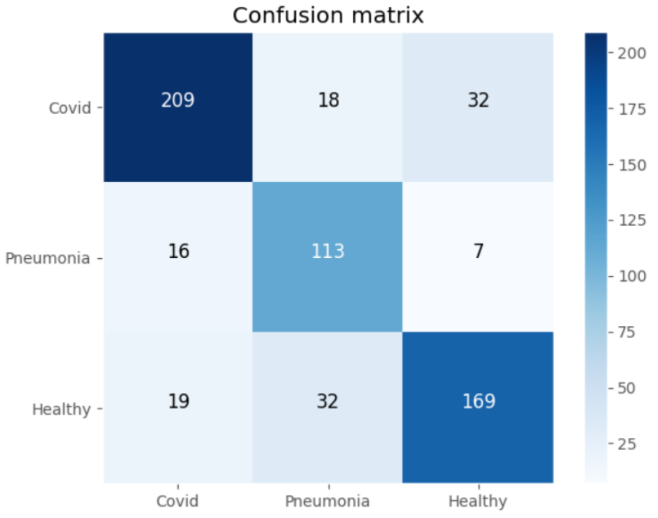

ROC curves

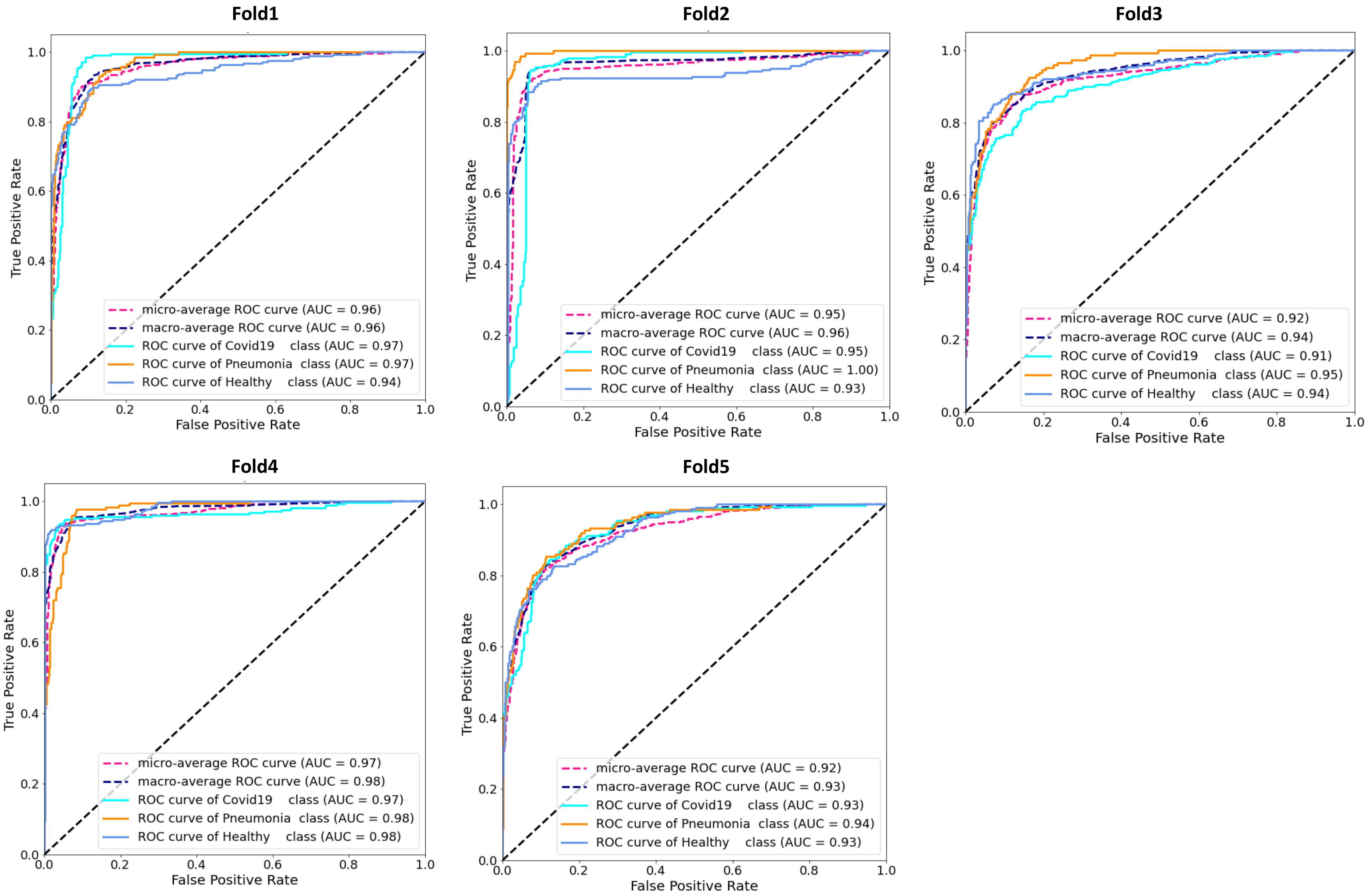
Fold1
True Positive Rate
False Positive Rate
micro-average ROC curve (AUC = 0.96)
macro-average ROC curve (AUC = 0.96)
ROC curve of Covid19 class (AUC = 0.97)
ROC curve of Pneumonia class (AUC = 0.97)
ROC curve of Healthy class (AUC = 0.94)
Fold2
True Positive Rate
False Positive Rate
micro-average ROC curve (AUC = 0.95)
macro-average ROC curve (AUC = 0.96)
ROC curve of Covid19 class (AUC = 0.95)
ROC curve of Pneumonia class (AUC = 1.00)
ROC curve of Healthy class (AUC = 0.93)
Fold3
True Positive Rate
False Positive Rate
micro-average ROC curve (AUC = 0.92)
macro-average ROC curve (AUC = 0.94)
ROC curve of Covid19 class (AUC = 0.91)
ROC curve of Pneumonia class (AUC = 0.95)
ROC curve of Healthy class (AUC = 0.94)
Fold4
True Positive Rate
False Positive Rate
micro-average ROC curve (AUC = 0.97)
macro-average ROC curve (AUC = 0.98)
ROC curve of Covid19 class (AUC = 0.97)
ROC curve of Pneumonia class (AUC = 0.98)
ROC curve of Healthy class (AUC = 0.98)
Fold5
True Positive Rate
False Positive Rate
micro-average ROC curve (AUC = 0.92)
macro-average ROC curve (AUC = 0.93)
ROC curve of Covid19 class (AUC = 0.93)
ROC curve of Pneumonia class (AUC = 0.94)
ROC curve of Healthy class (AUC = 0.93)

3×5 −Fold Cross-Validation

Learning curves

Fold1

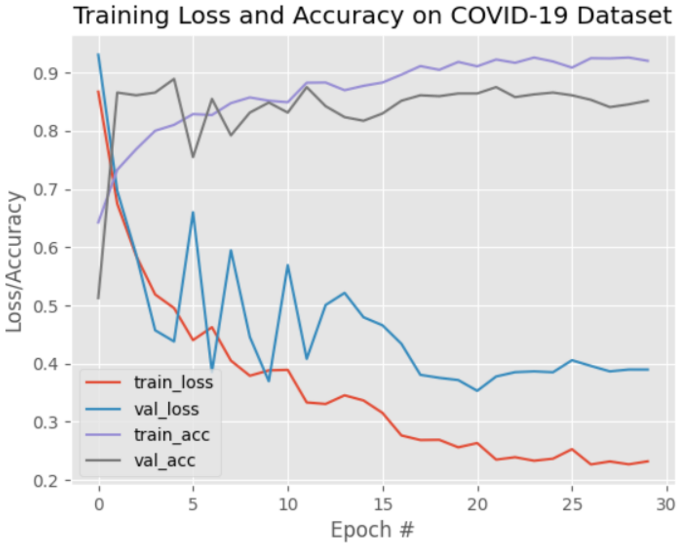

Fold2

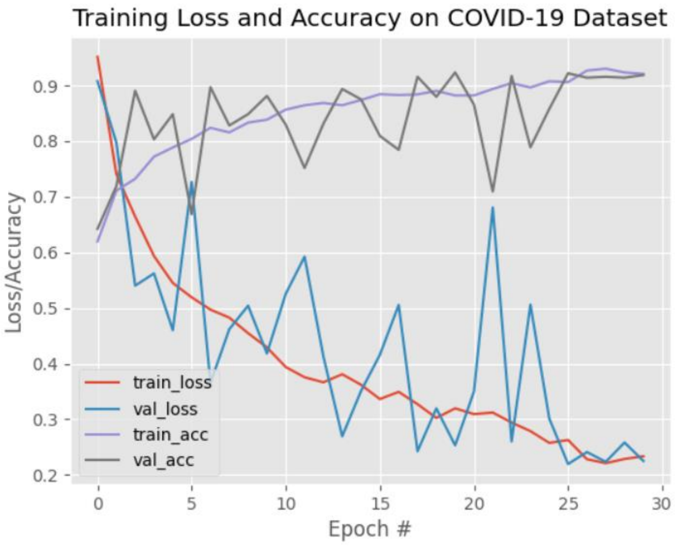

Fold3

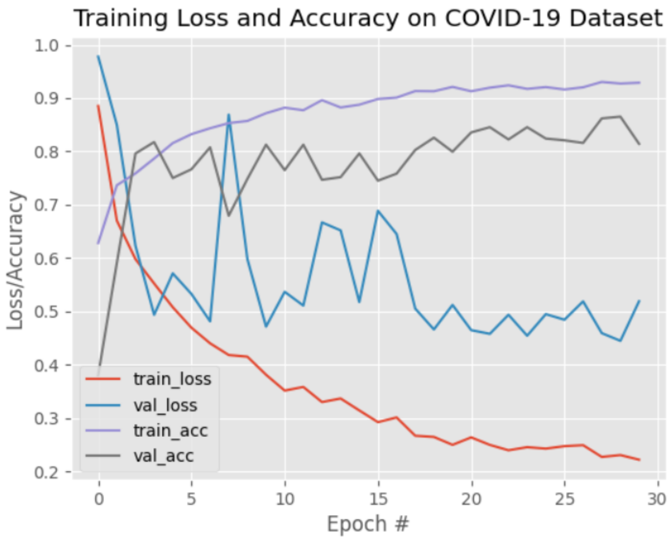

Fold4

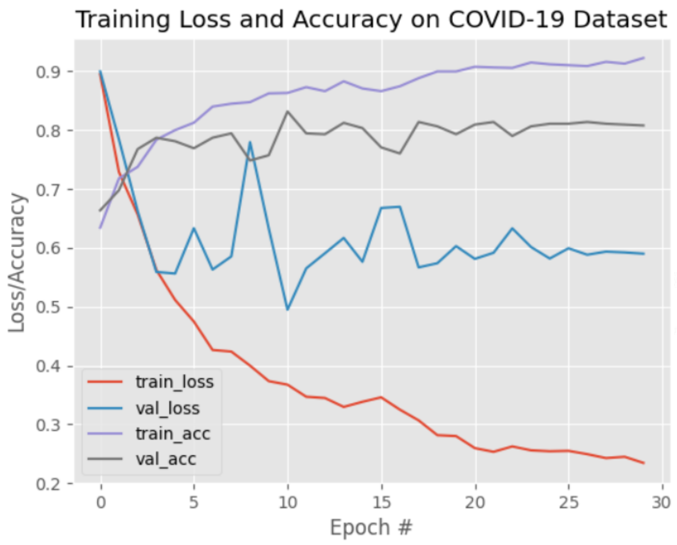

Fold5

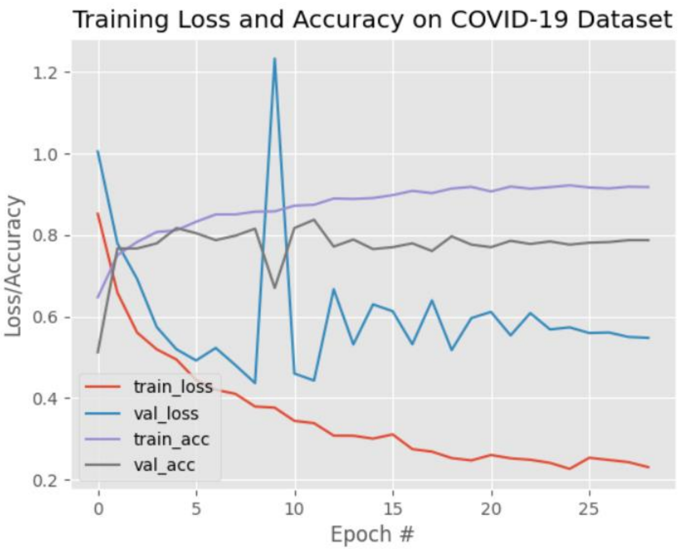

Confusion matrices

Fold1

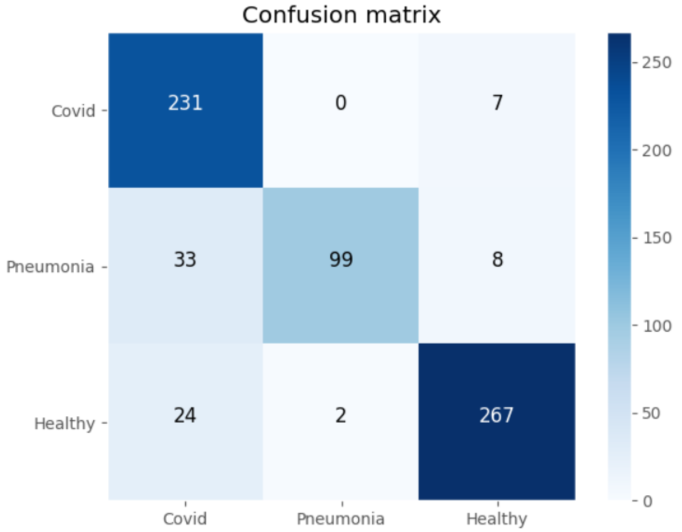

Fold2

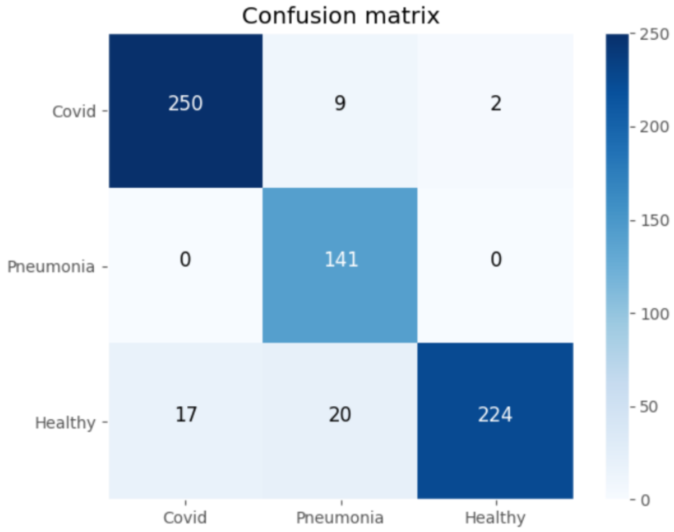

Fold3

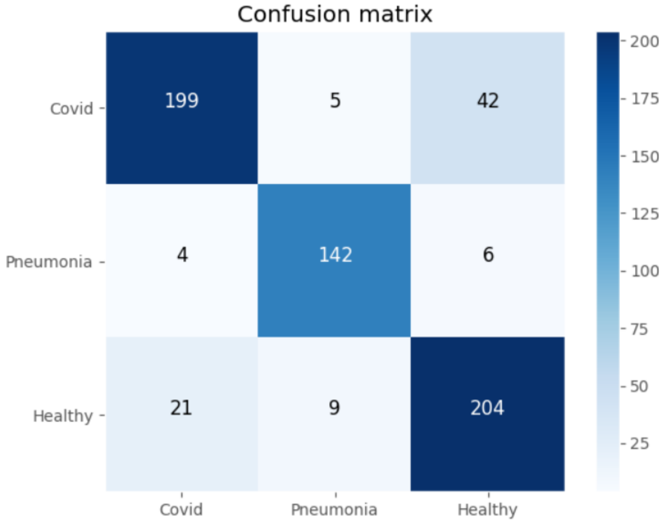

Fold4

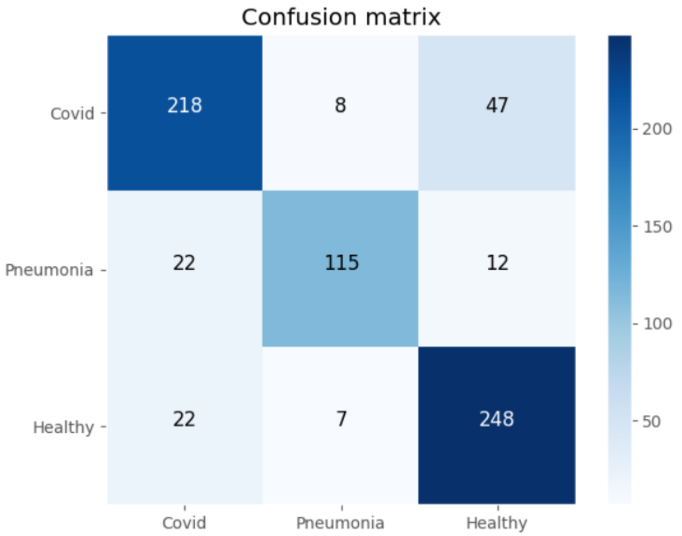

Fold5

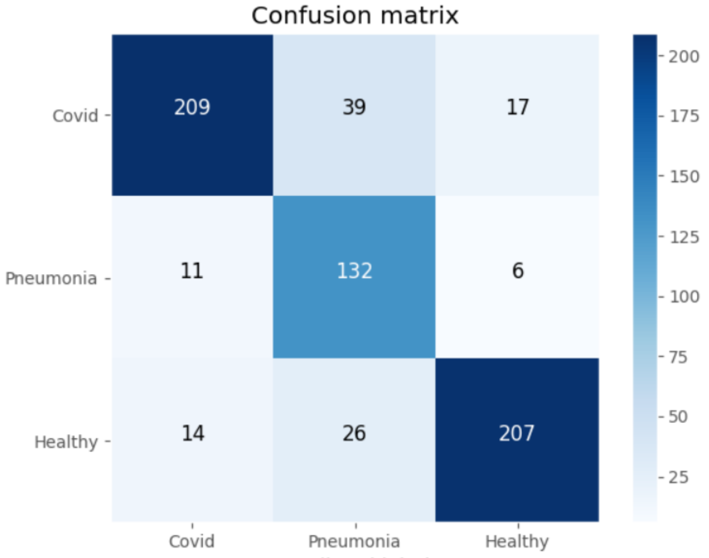

ROC curves

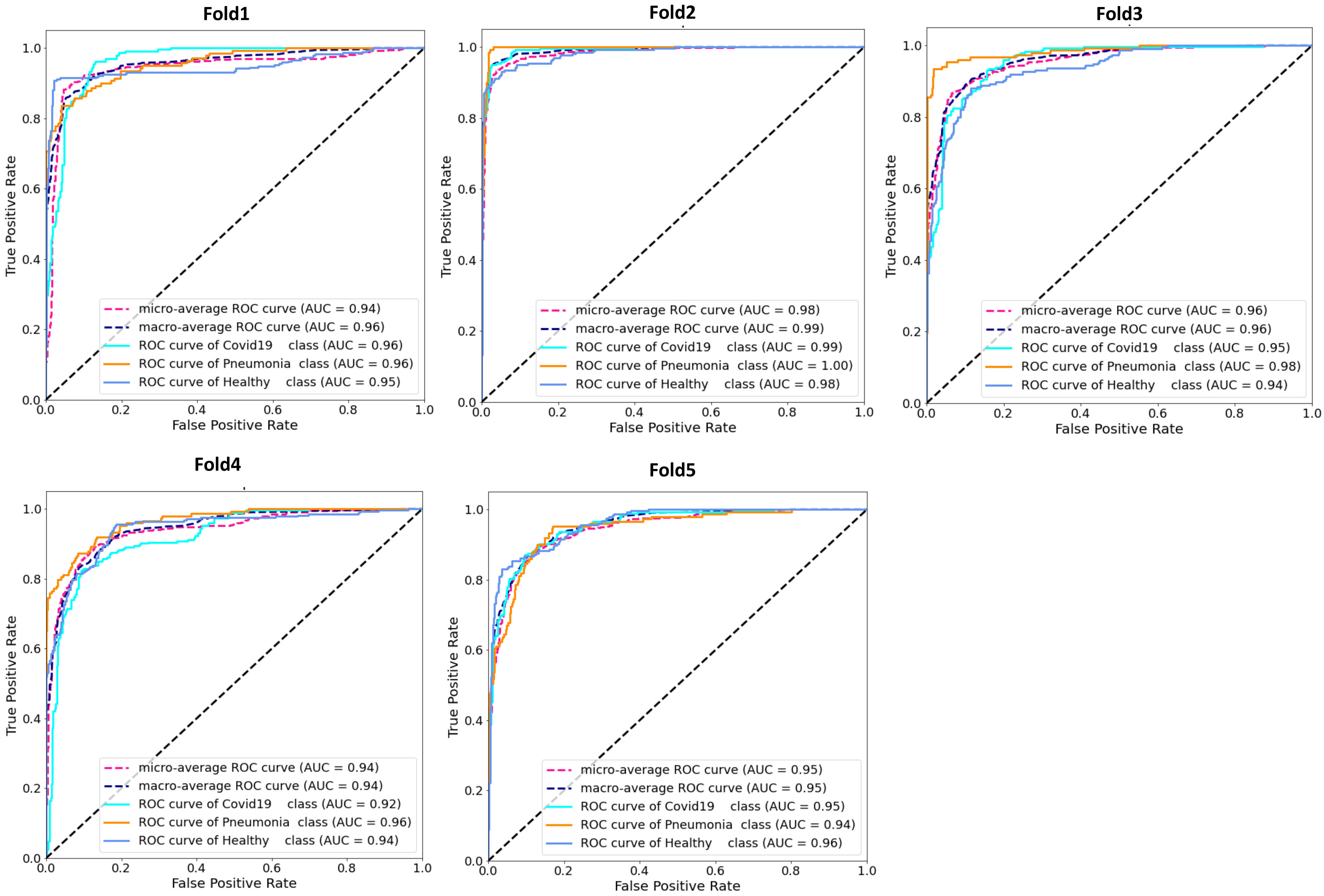
Fold1
Fold2
Fold3
Fold4
Fold5
True Positive Rate
False Positive Rate
0.0
0.2
0.4
0.6
0.8
1.0
micro-average ROC curve (AUC = 0.94)
macro-average ROC curve (AUC = 0.96)
ROC curve of Covid19 class (AUC = 0.96)
ROC curve of Pneumonia class (AUC = 0.96)
ROC curve of Healthy class (AUC = 0.95)
micro-average ROC curve (AUC = 0.98)
macro-average ROC curve (AUC = 0.99)
ROC curve of Covid19 class (AUC = 0.99)
ROC curve of Pneumonia class (AUC = 1.00)
ROC curve of Healthy class (AUC = 0.98)
micro-average ROC curve (AUC = 0.96)
macro-average ROC curve (AUC = 0.96)
ROC curve of Covid19 class (AUC = 0.95)
ROC curve of Pneumonia class (AUC = 0.98)
ROC curve of Healthy class (AUC = 0.94)
micro-average ROC curve (AUC = 0.94)
macro-average ROC curve (AUC = 0.94)
ROC curve of Covid19 class (AUC = 0.92)
ROC curve of Pneumonia class (AUC = 0.96)
ROC curve of Healthy class (AUC = 0.94)
micro-average ROC curve (AUC = 0.95)
macro-average ROC curve (AUC = 0.95)
ROC curve of Covid19 class (AUC = 0.95)
ROC curve of Pneumonia class (AUC = 0.94)
ROC curve of Healthy class (AUC = 0.96)

4×5 −Fold Cross-Validation

Learning curves

Fold1

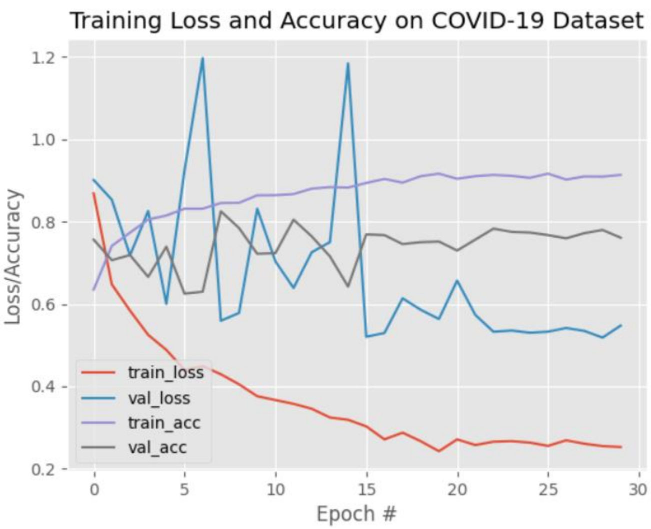

Fold2

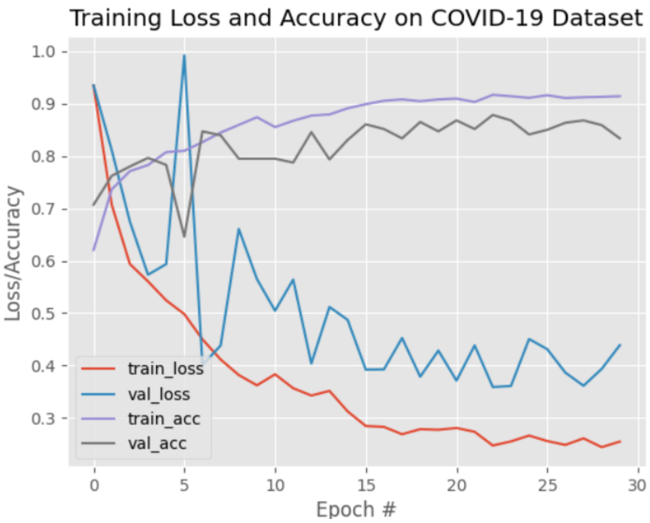

Fold3

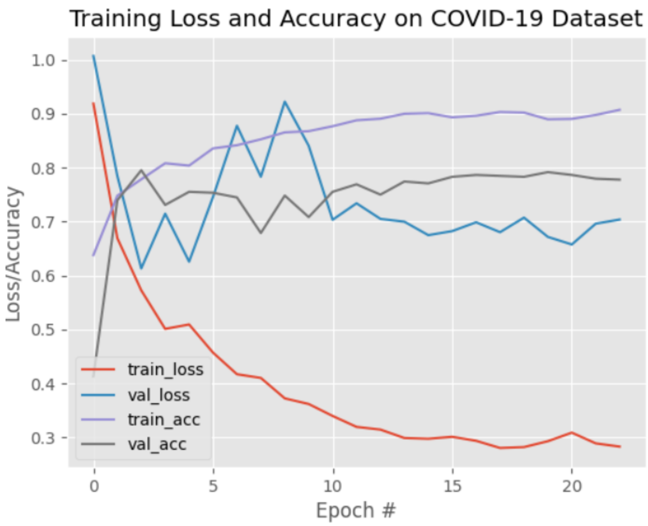

Fold4

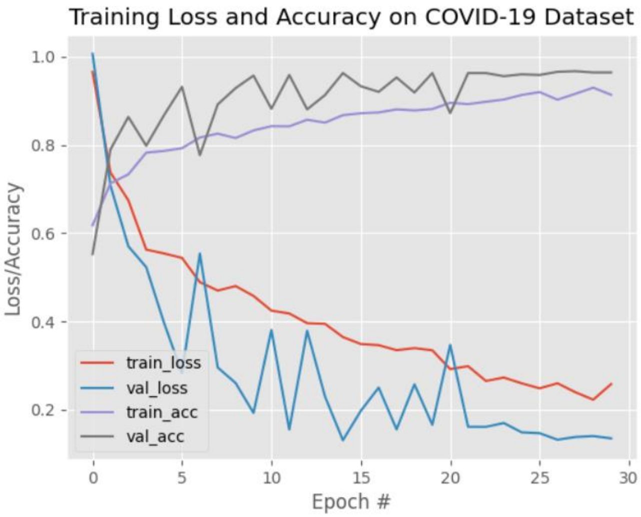

Fold5

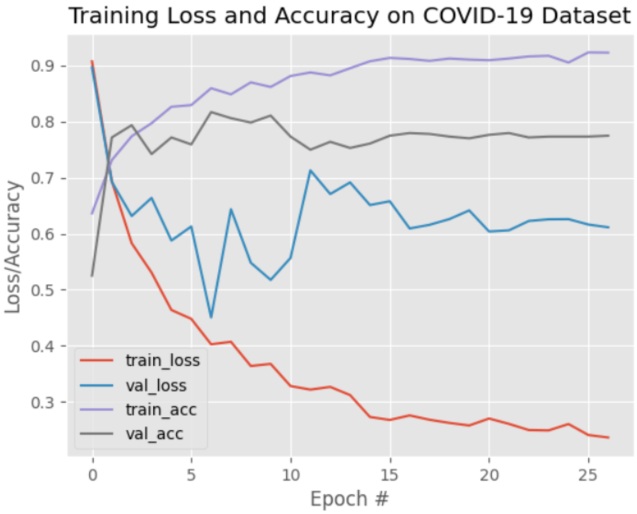

Confusion matrices

Fold1

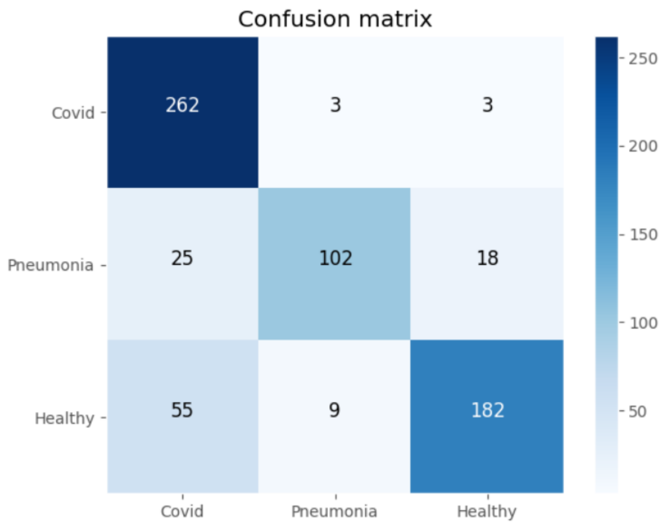

Fold2

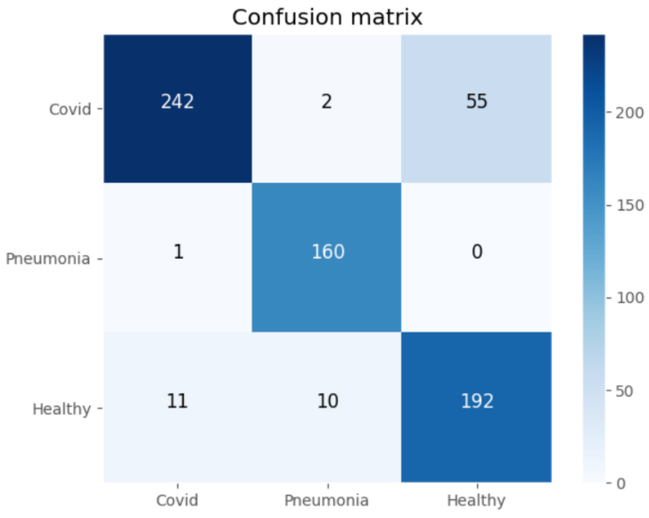

Fold3

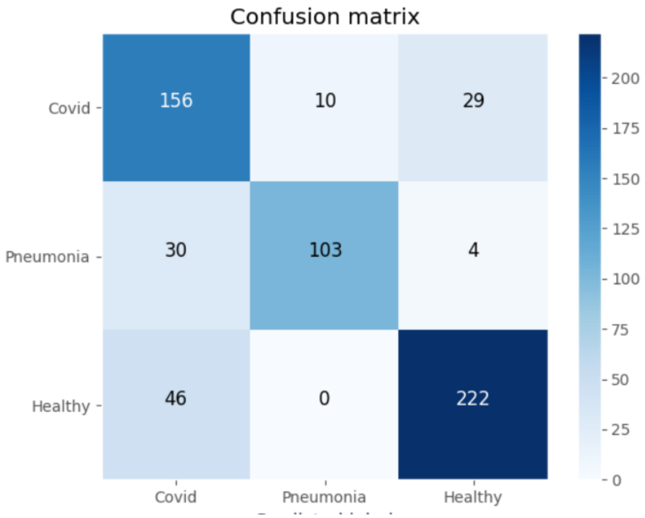

Fold4

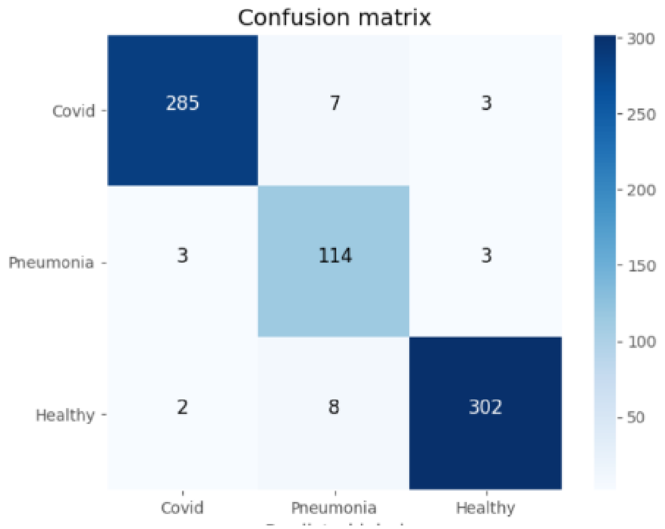

Fold5

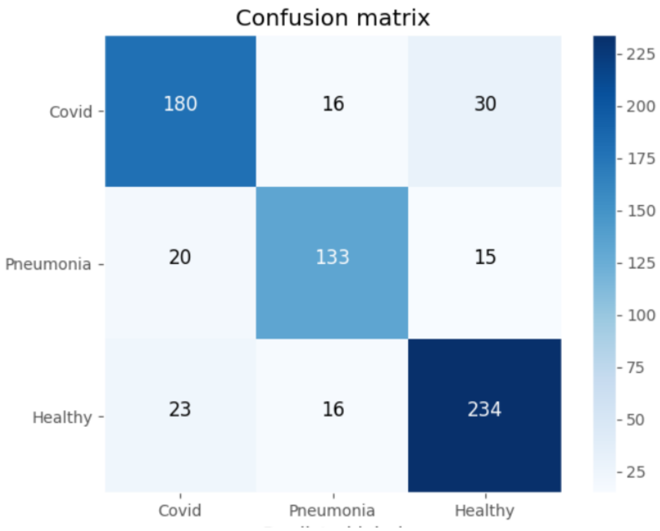

ROC curves

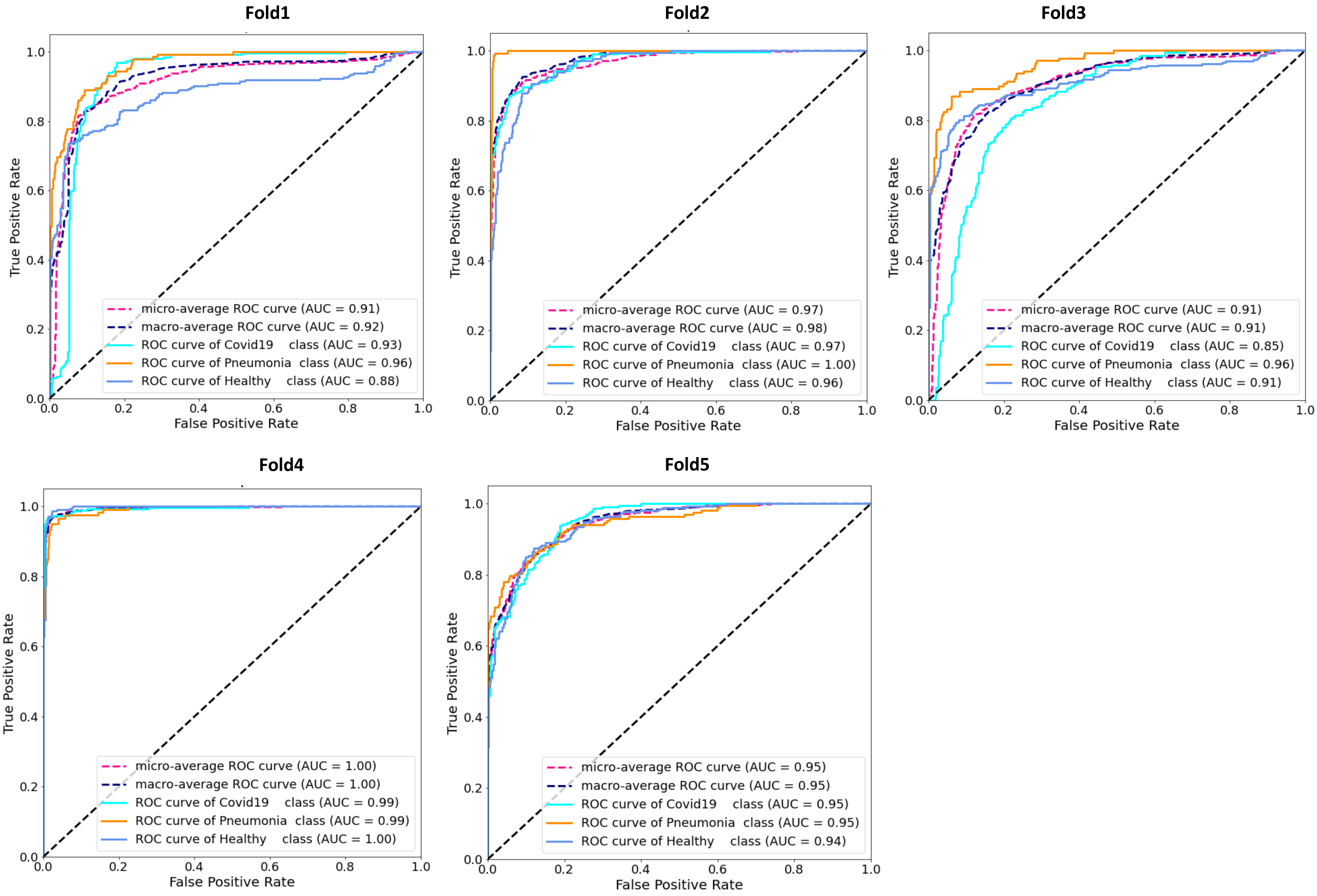
Fold1
True Positive Rate
False Positive Rate
micro-average ROC curve (AUC = 0.91)
macro-average ROC curve (AUC = 0.92)
ROC curve of Covid19 class (AUC = 0.93)
ROC curve of Pneumonia class (AUC = 0.96)
ROC curve of Healthy class (AUC = 0.88)
Fold2
True Positive Rate
False Positive Rate
micro-average ROC curve (AUC = 0.97)
macro-average ROC curve (AUC = 0.98)
ROC curve of Covid19 class (AUC = 0.97)
ROC curve of Pneumonia class (AUC = 1.00)
ROC curve of Healthy class (AUC = 0.96)
Fold3
True Positive Rate
False Positive Rate
micro-average ROC curve (AUC = 0.91)
macro-average ROC curve (AUC = 0.91)
ROC curve of Covid19 class (AUC = 0.85)
ROC curve of Pneumonia class (AUC = 0.96)
ROC curve of Healthy class (AUC = 0.91)
Fold4
True Positive Rate
False Positive Rate
micro-average ROC curve (AUC = 1.00)
macro-average ROC curve (AUC = 1.00)
ROC curve of Covid19 class (AUC = 0.99)
ROC curve of Pneumonia class (AUC = 0.99)
ROC curve of Healthy class (AUC = 1.00)
Fold5
True Positive Rate
False Positive Rate
micro-average ROC curve (AUC = 0.95)
macro-average ROC curve (AUC = 0.95)
ROC curve of Covid19 class (AUC = 0.95)
ROC curve of Pneumonia class (AUC = 0.95)
ROC curve of Healthy class (AUC = 0.94)

5×5 −Fold Cross-Validation

Learning curves

Fold1

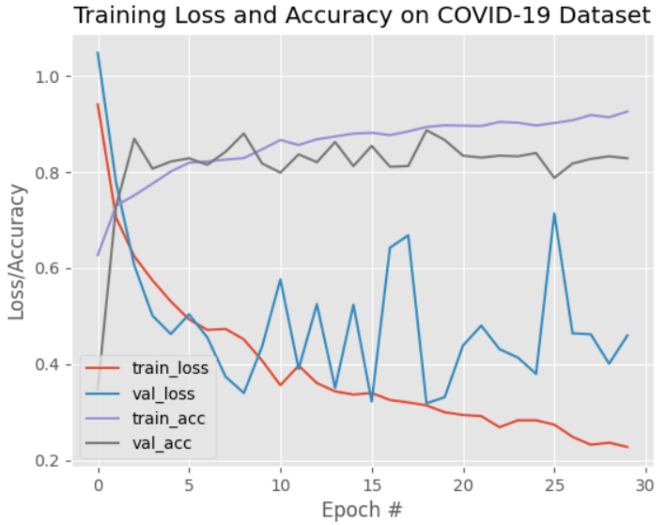

Fold2

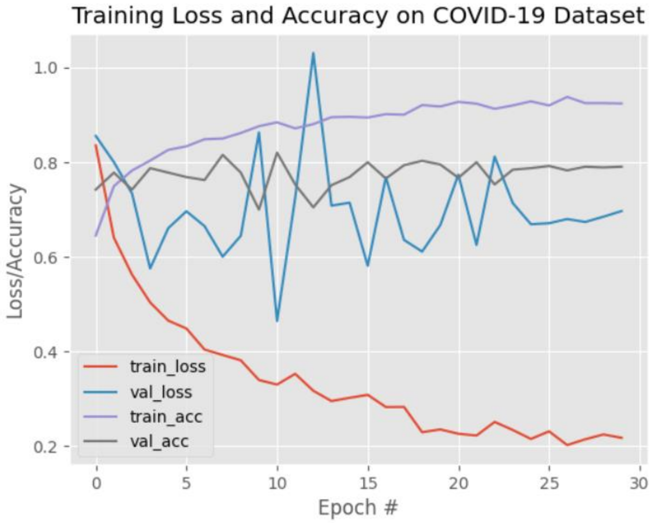

Fold3

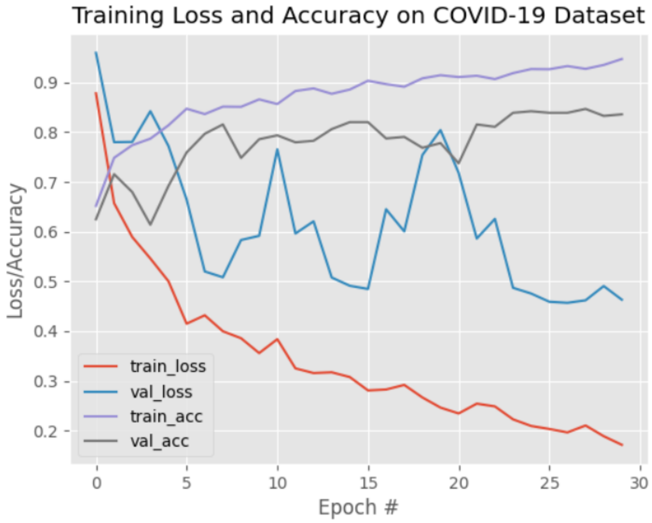

Fold4

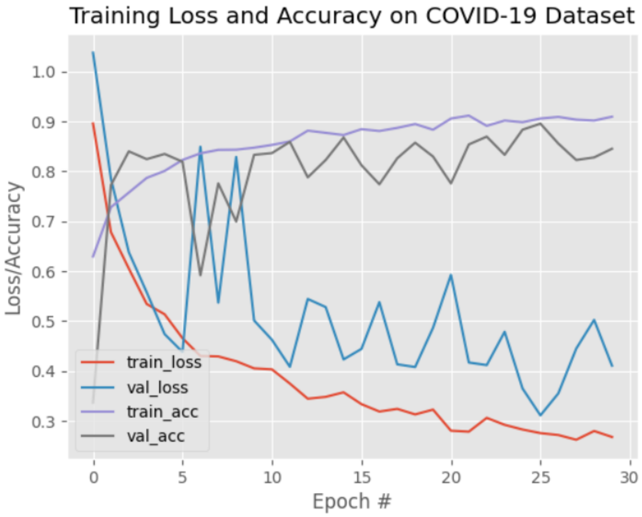

Fold5

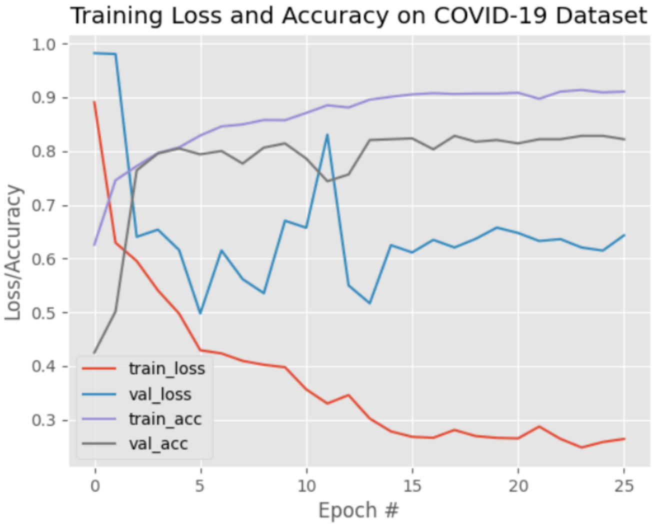

Confusion matrices

Fold1

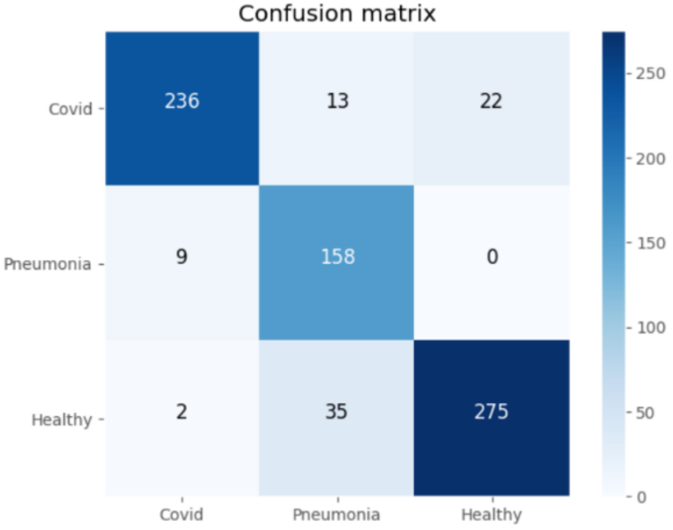

Fold2

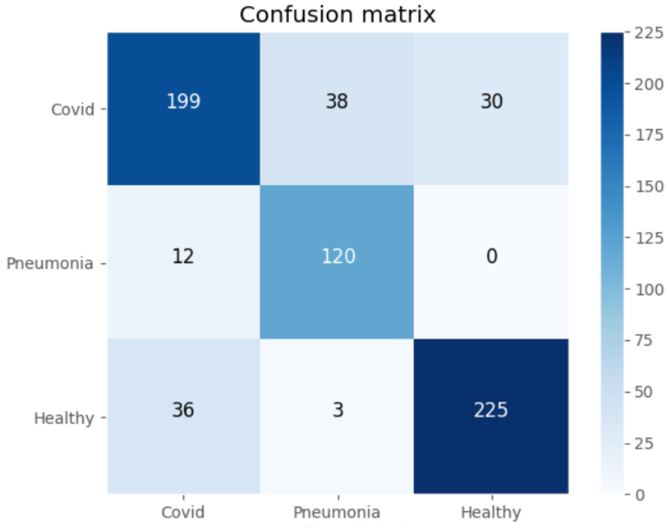

Fold3

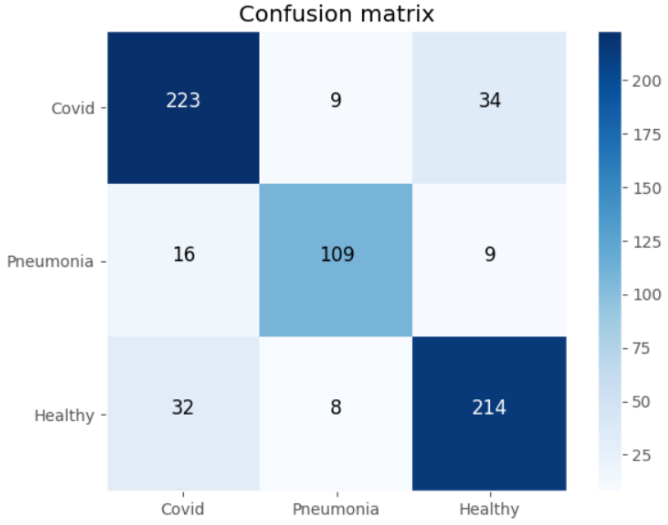

Fold4

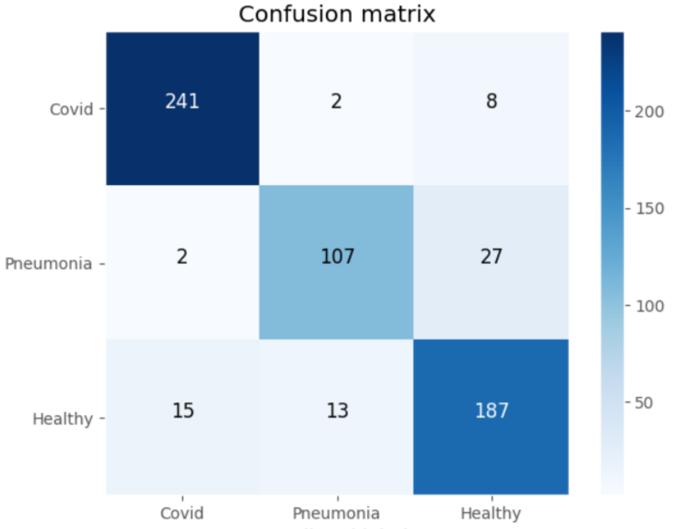

Fold5

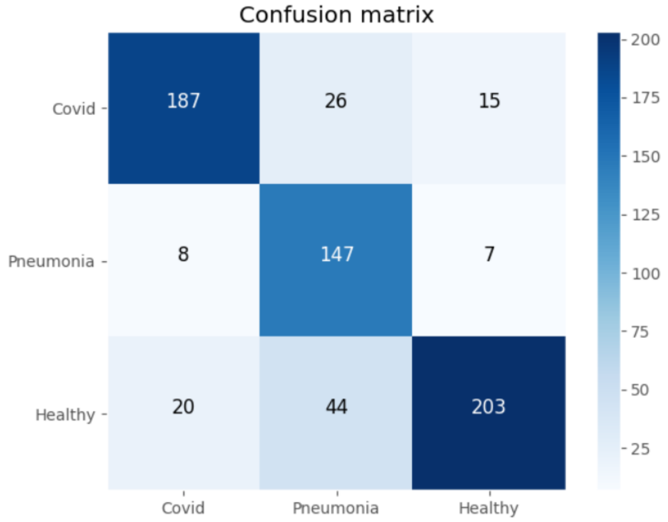

ROC curves

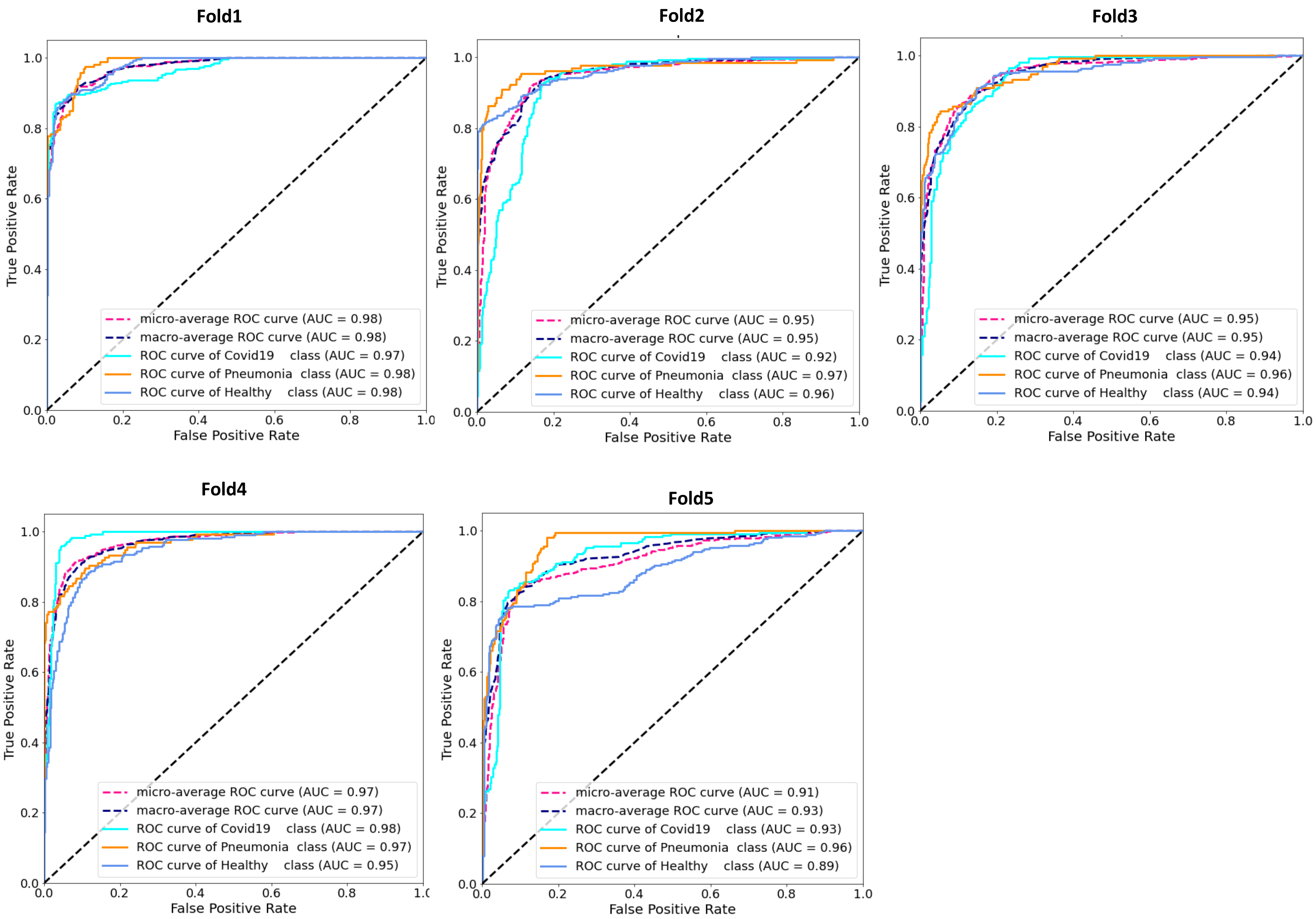
Fold1
True Positive Rate
False Positive Rate
0.0
0.2
0.4
0.6
0.8
1.0
micro-average ROC curve (AUC = 0.98)
macro-average ROC curve (AUC = 0.98)
ROC curve of Covid19 class (AUC = 0.97)
ROC curve of Pneumonia class (AUC = 0.98)
ROC curve of Healthy class (AUC = 0.98)
Fold2
True Positive Rate
False Positive Rate
micro-average ROC curve (AUC = 0.95)
macro-average ROC curve (AUC = 0.95)
ROC curve of Covid19 class (AUC = 0.92)
ROC curve of Pneumonia class (AUC = 0.97)
ROC curve of Healthy class (AUC = 0.96)
Fold3
True Positive Rate
False Positive Rate
micro-average ROC curve (AUC = 0.95)
macro-average ROC curve (AUC = 0.95)
ROC curve of Covid19 class (AUC = 0.94)
ROC curve of Pneumonia class (AUC = 0.96)
ROC curve of Healthy class (AUC = 0.94)
Fold4
True Positive Rate
False Positive Rate
micro-average ROC curve (AUC = 0.97)
macro-average ROC curve (AUC = 0.97)
ROC curve of Covid19 class (AUC = 0.98)
ROC curve of Pneumonia class (AUC = 0.97)
ROC curve of Healthy class (AUC = 0.95)
Fold5
True Positive Rate
False Positive Rate
micro-average ROC curve (AUC = 0.91)
macro-average ROC curve (AUC = 0.93)
ROC curve of Covid19 class (AUC = 0.93)
ROC curve of Pneumonia class (AUC = 0.96)
ROC curve of Healthy class (AUC = 0.89)
