# Supplementary material for: Deep-learning based detection of COVID-19 using lung ultrasound imagery
Source: PLoS One. 2021 Aug 13;16(8):e0255886. doi: 10.1371/journal.pone.0255886 (PMC8363024; doi:10.1371/journal.pone.0255886)
Supplement: S4 File — Detailed learning curves, confusion matrices, and AUC-ROC curves of each repeated 5 × 5-fold cross-validation experiments. (PDF) [file pone.0255886.s004.pdf]

InceptionV3-based model

1×5 –Fold Cross-Validation

Learning curves

Fold1

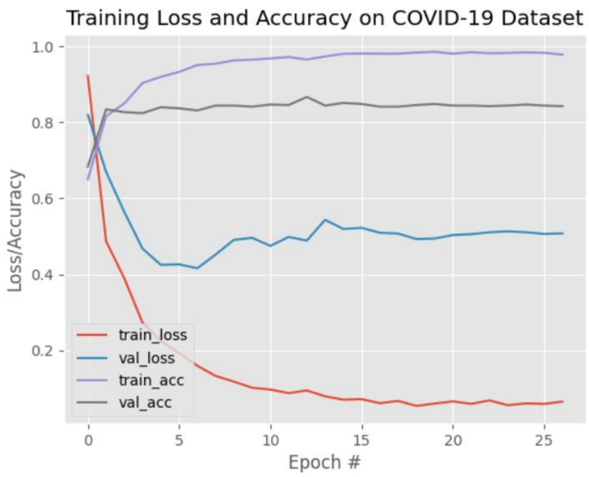

Fold2

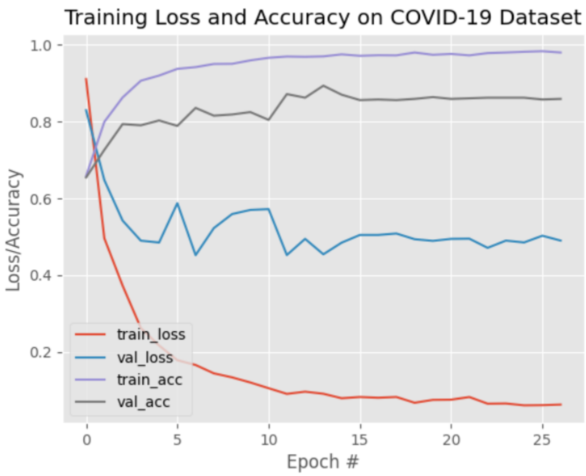

Fold3

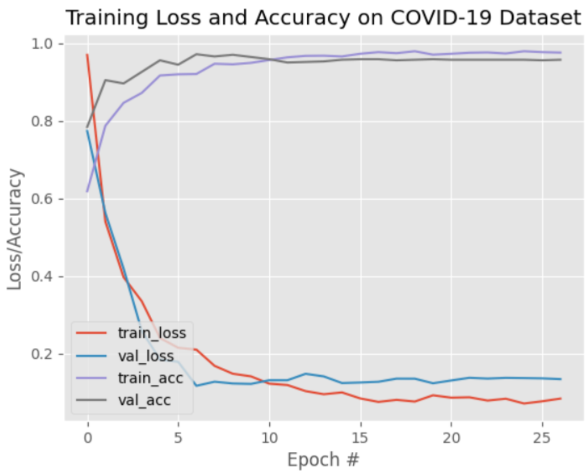

Fold4

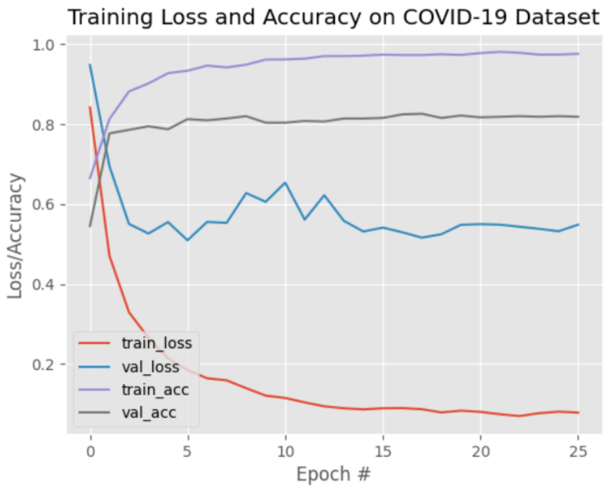

Fold5

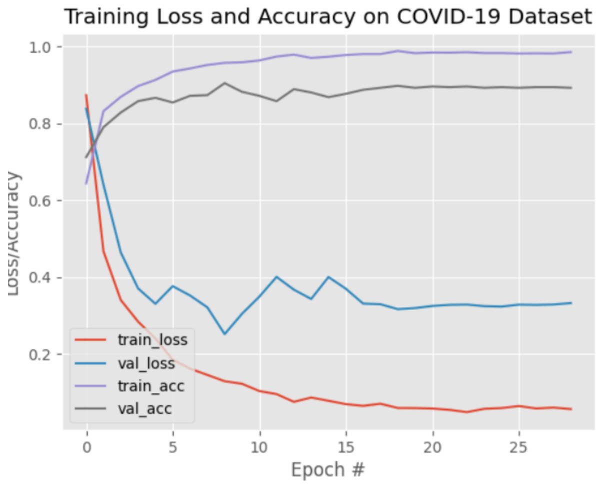

Confusion matrices

Fold1

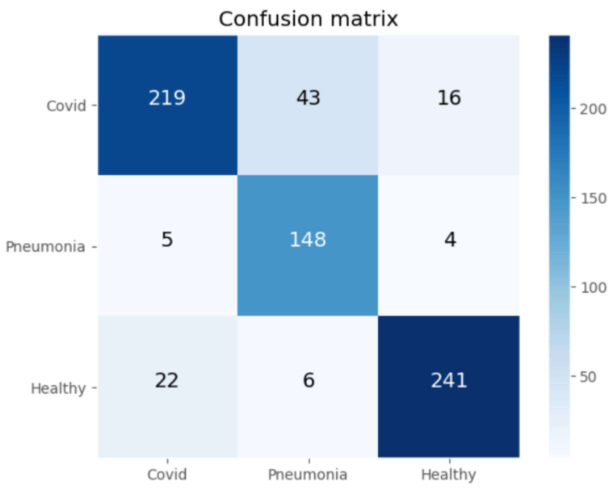

Fold2

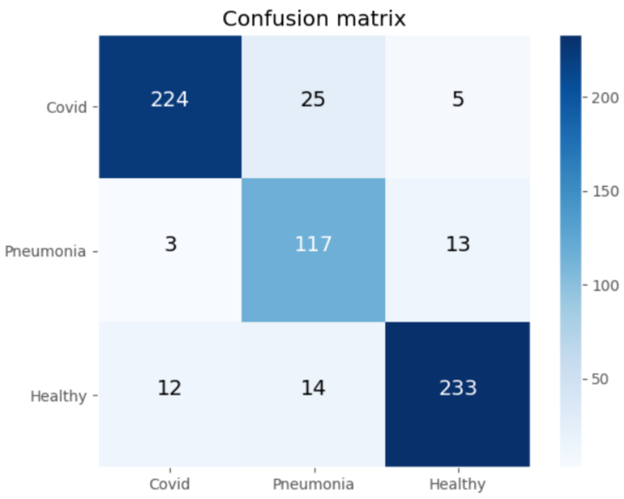

Fold3

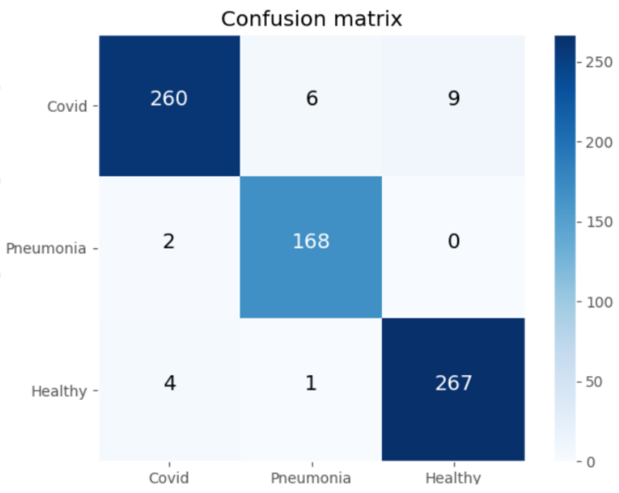

Fold4

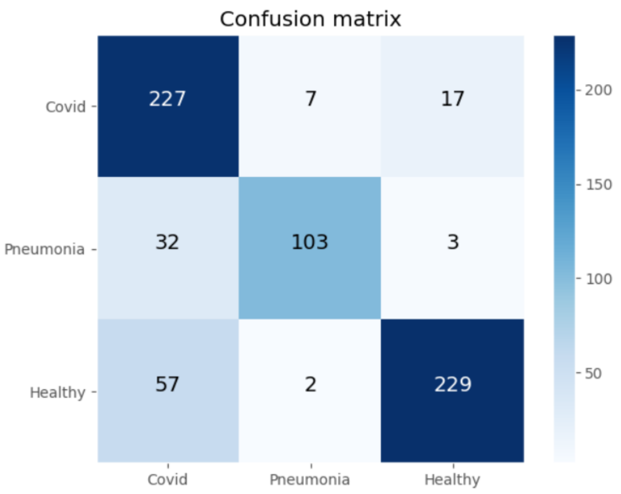

Fold5

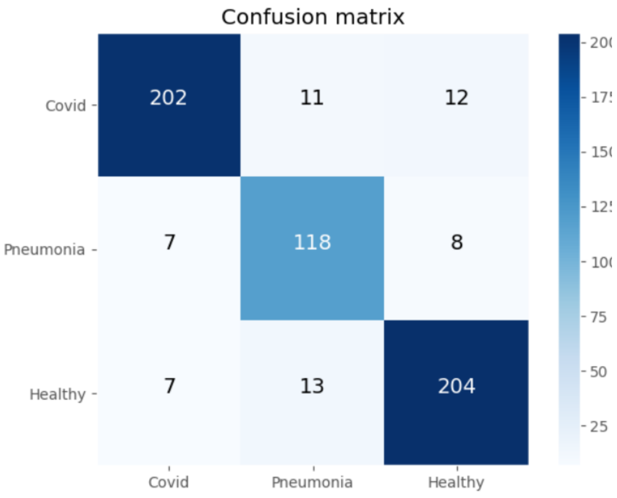

AUC-ROC curves

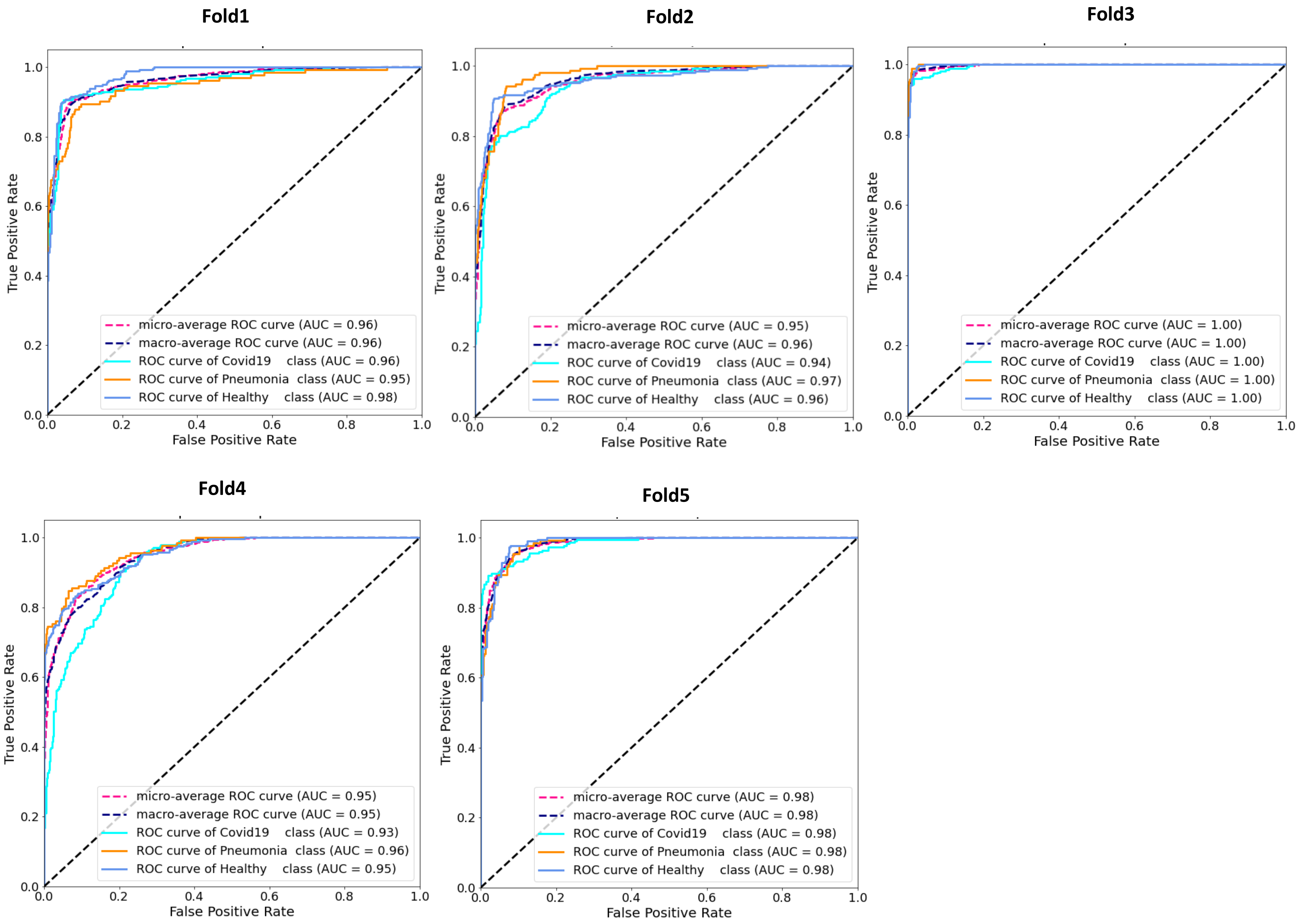

2 × 5 −Fold Cross-Validation

Learning curves

Fold1

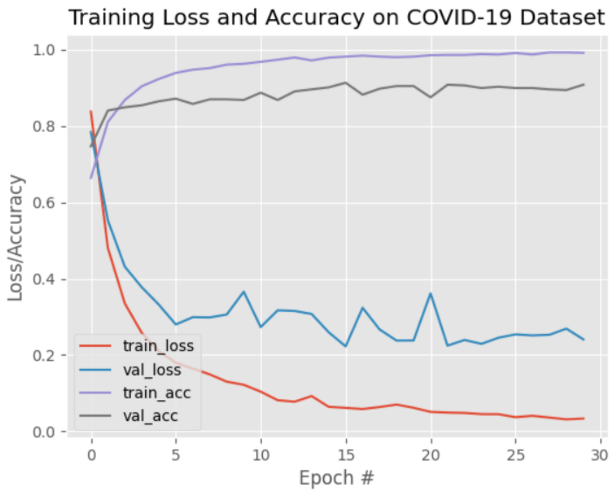

Fold2

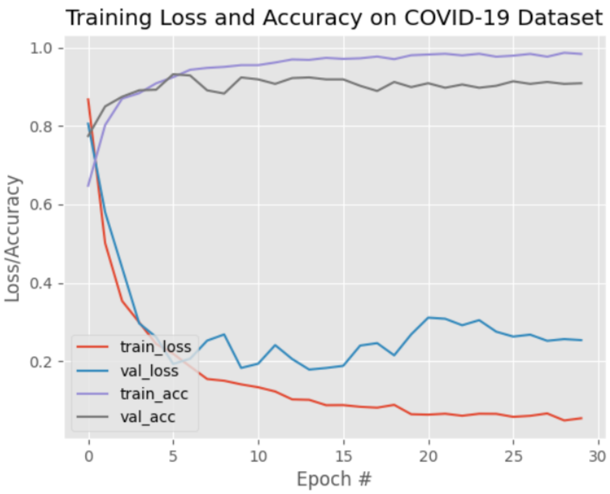

Fold3

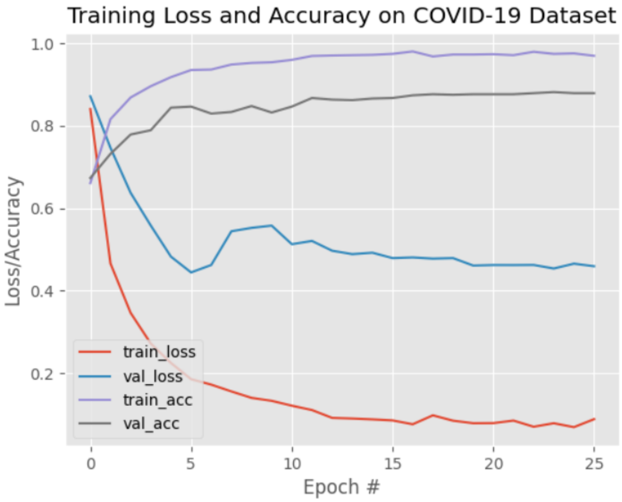

Fold4

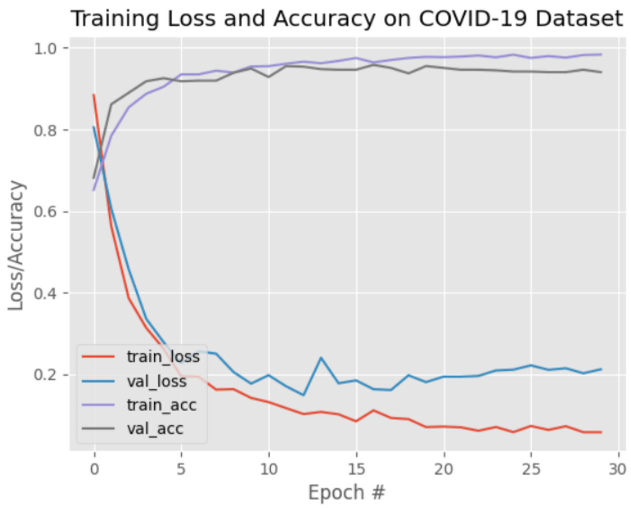

Fold5

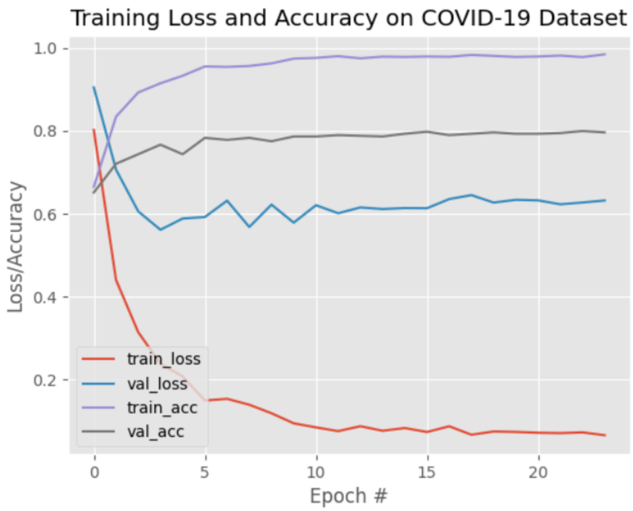

Confusion matrices

Fold1

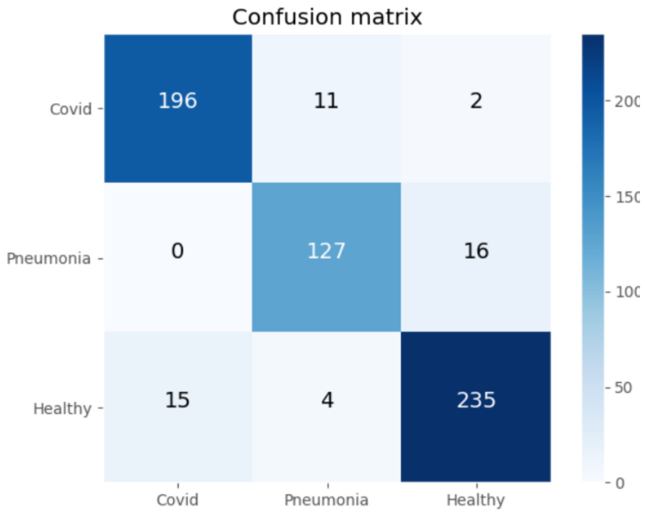

Fold2

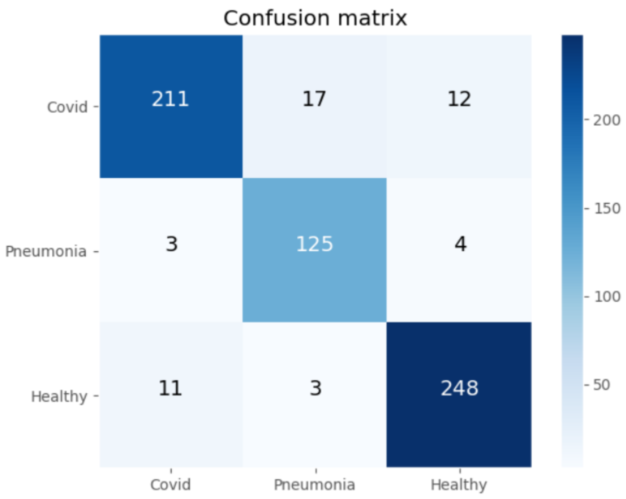

Fold3

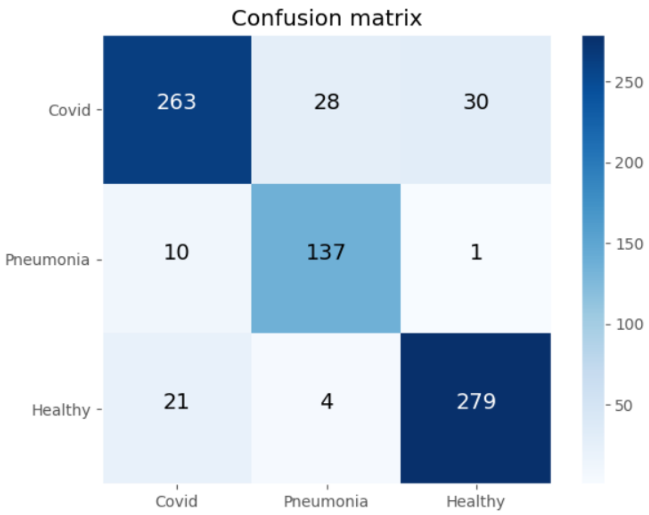

Fold4

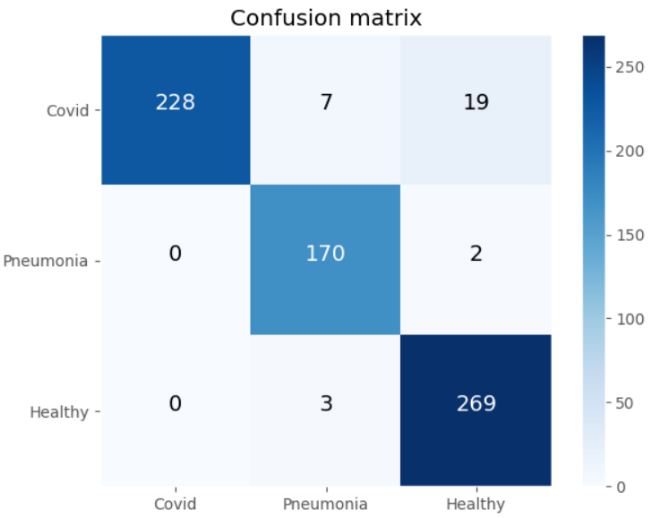

Fold5

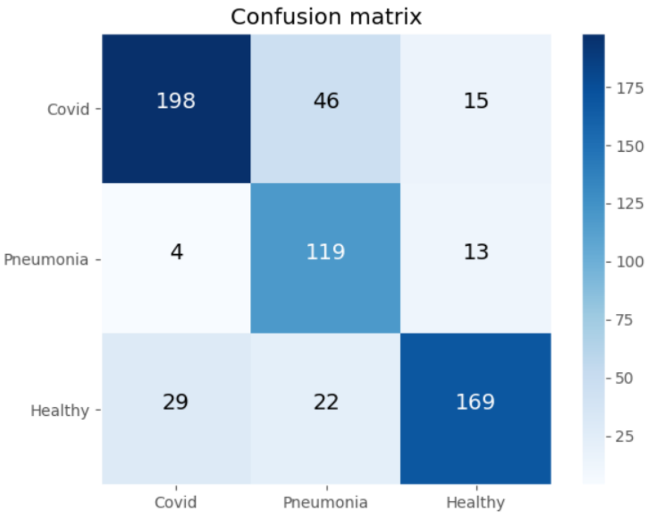

ROC curves

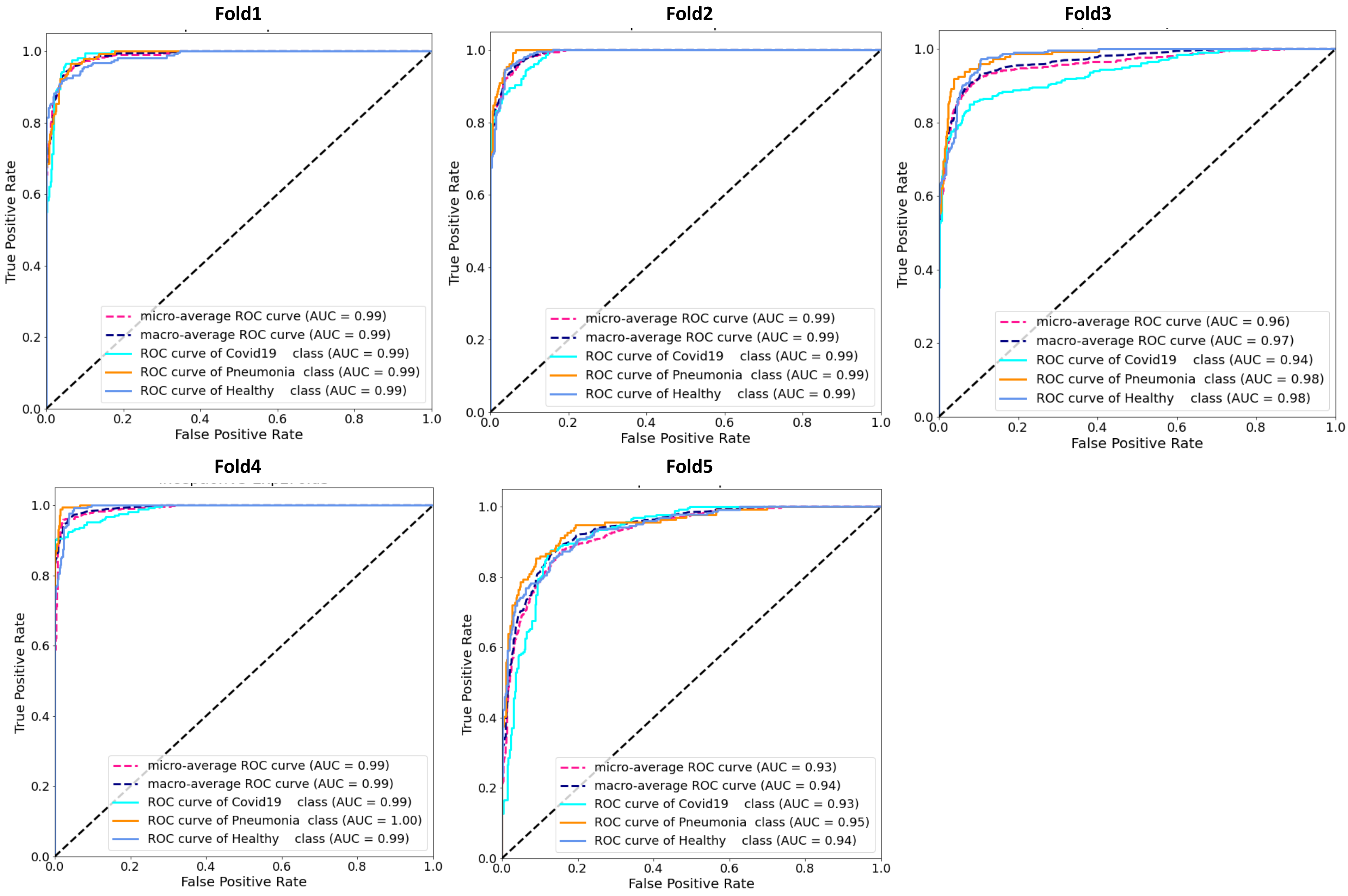
Fold1
True Positive Rate
False Positive Rate
micro-average ROC curve (AUC = 0.99)
macro-average ROC curve (AUC = 0.99)
ROC curve of Covid19 class (AUC = 0.99)
ROC curve of Pneumonia class (AUC = 0.99)
ROC curve of Healthy class (AUC = 0.99)
Fold2
True Positive Rate
False Positive Rate
micro-average ROC curve (AUC = 0.99)
macro-average ROC curve (AUC = 0.99)
ROC curve of Covid19 class (AUC = 0.99)
ROC curve of Pneumonia class (AUC = 0.99)
ROC curve of Healthy class (AUC = 0.99)
Fold3
True Positive Rate
False Positive Rate
micro-average ROC curve (AUC = 0.96)
macro-average ROC curve (AUC = 0.97)
ROC curve of Covid19 class (AUC = 0.94)
ROC curve of Pneumonia class (AUC = 0.98)
ROC curve of Healthy class (AUC = 0.98)
Fold4
True Positive Rate
False Positive Rate
micro-average ROC curve (AUC = 0.99)
macro-average ROC curve (AUC = 0.99)
ROC curve of Covid19 class (AUC = 0.99)
ROC curve of Pneumonia class (AUC = 1.00)
ROC curve of Healthy class (AUC = 0.99)
Fold5
True Positive Rate
False Positive Rate
micro-average ROC curve (AUC = 0.93)
macro-average ROC curve (AUC = 0.94)
ROC curve of Covid19 class (AUC = 0.93)
ROC curve of Pneumonia class (AUC = 0.95)
ROC curve of Healthy class (AUC = 0.94)

3×5 −Fold Cross-Validation

Learning curves

Fold1

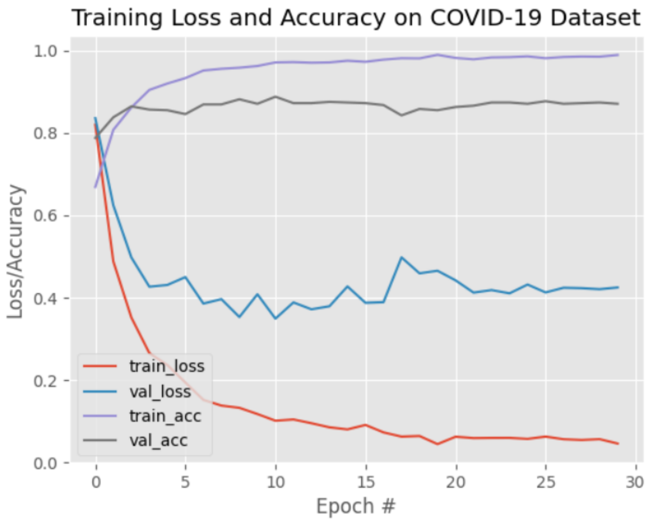

Fold2

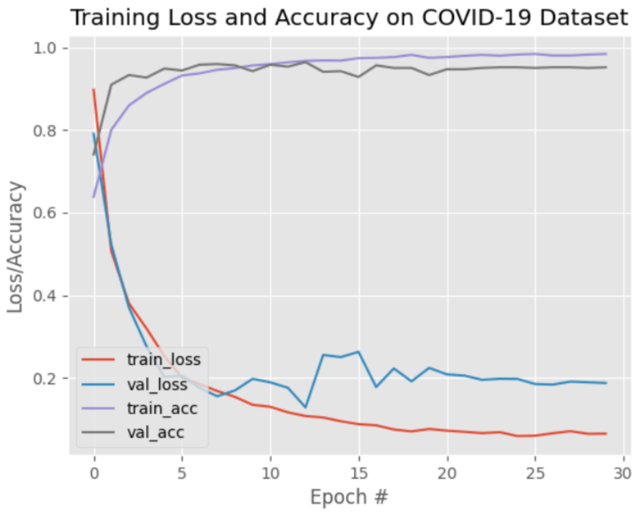

Fold3

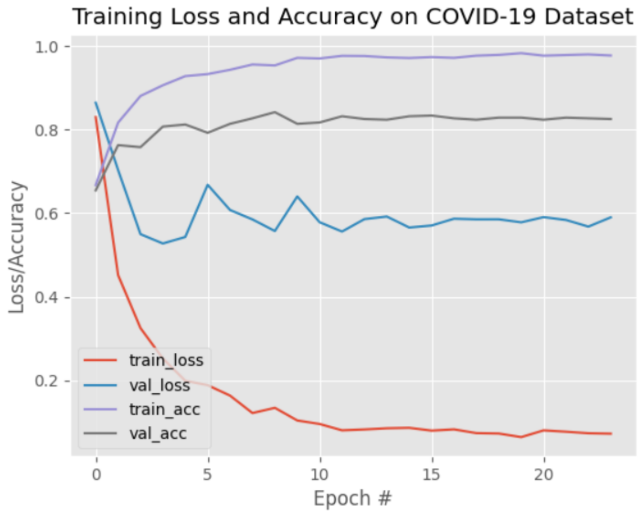

Fold4

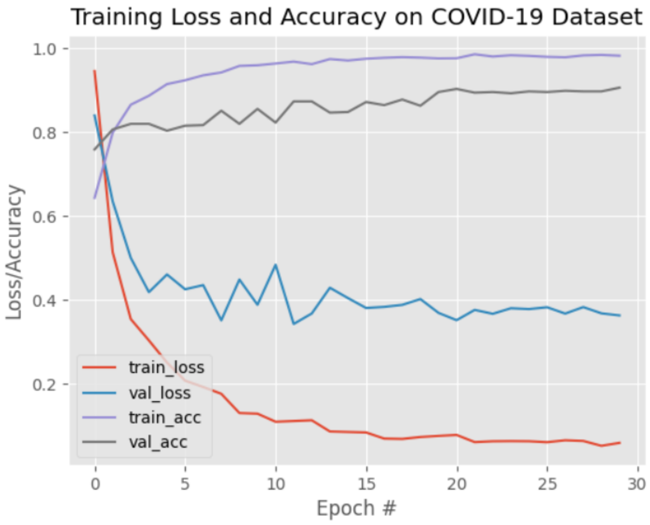

Fold5

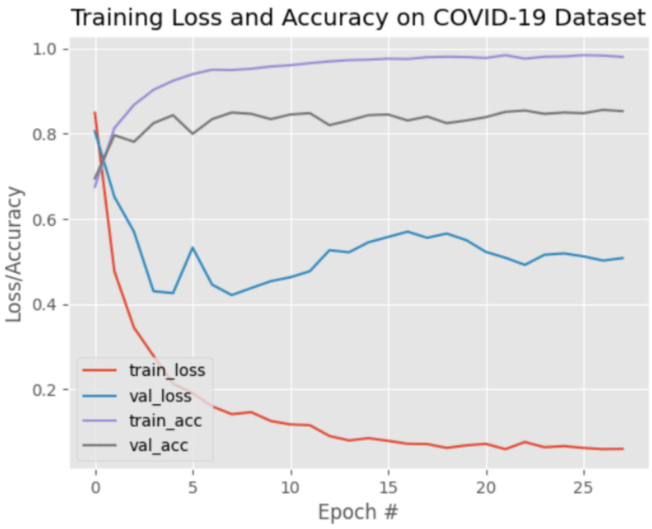

Confusion matrices

Fold1

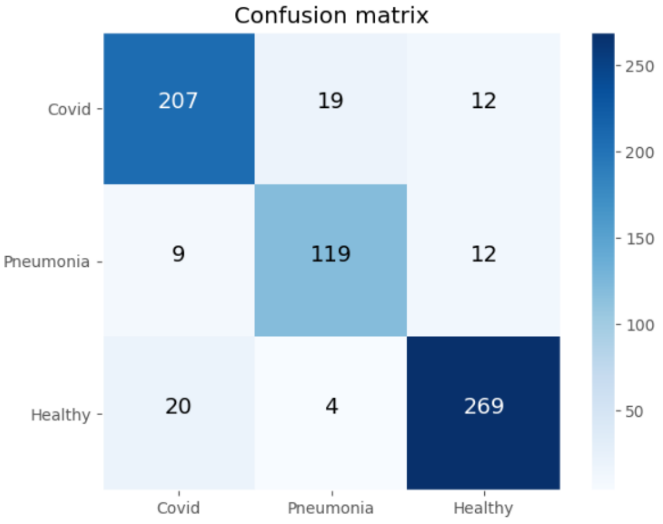

Fold2

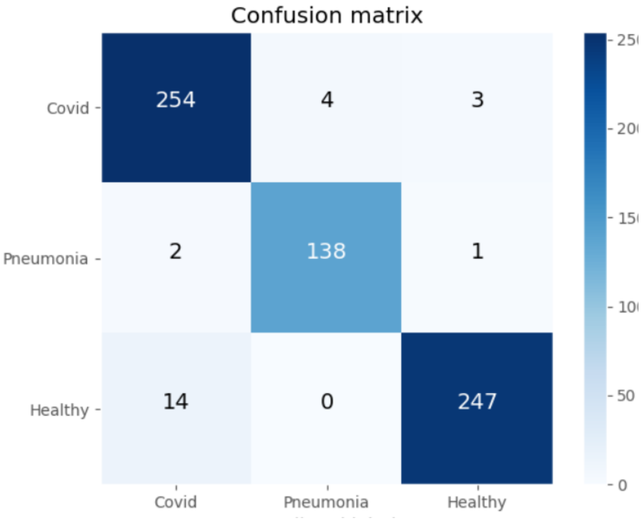

Fold3

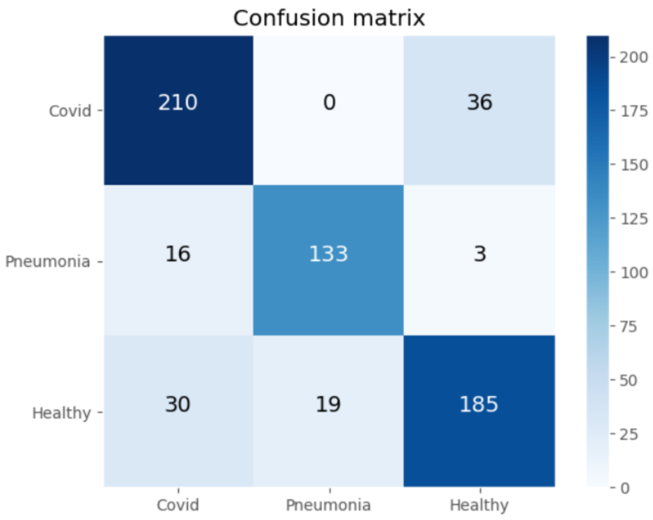

Fold4

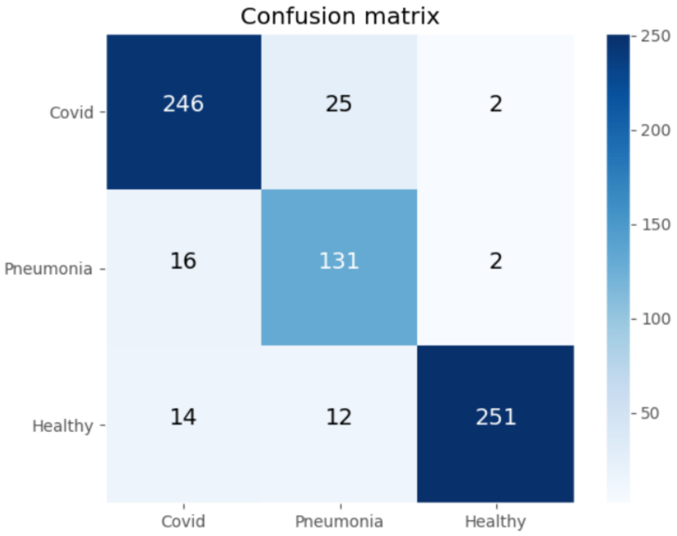

Fold5

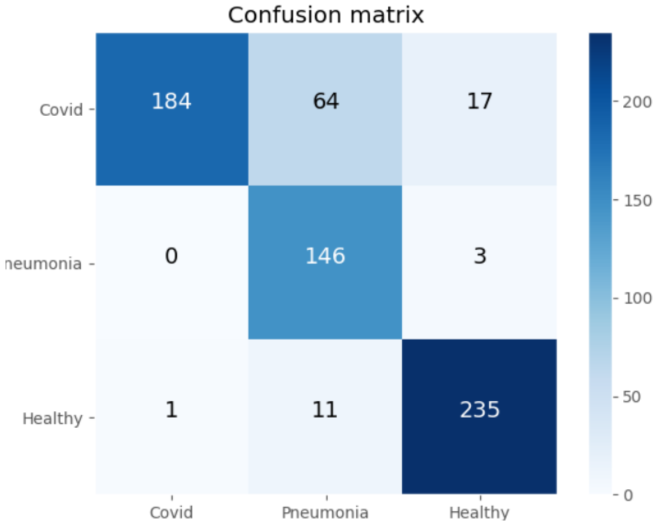

ROC curves

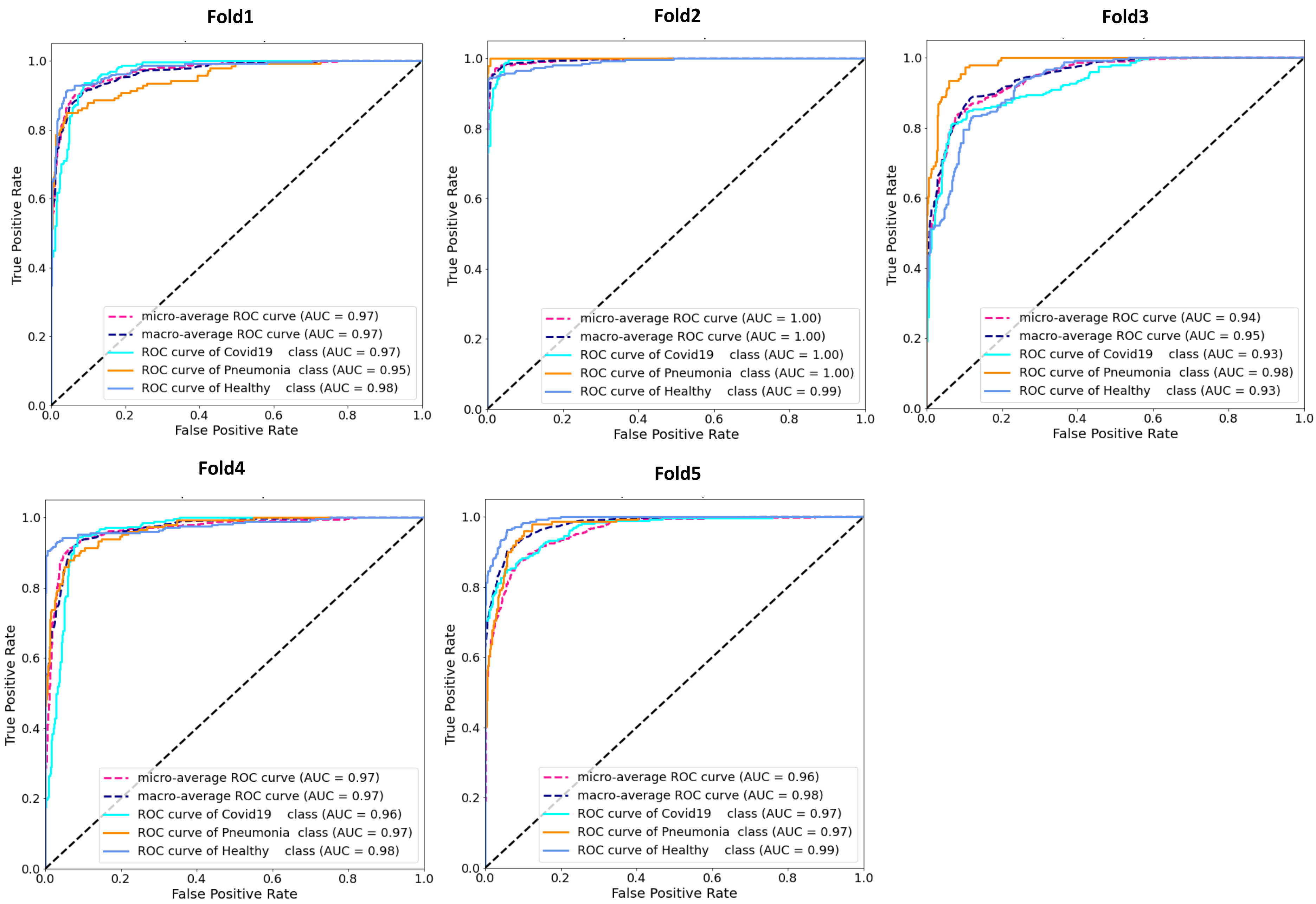
Fold1
True Positive Rate
False Positive Rate
micro-average ROC curve (AUC = 0.97)
macro-average ROC curve (AUC = 0.97)
ROC curve of Covid19 class (AUC = 0.97)
ROC curve of Pneumonia class (AUC = 0.95)
ROC curve of Healthy class (AUC = 0.98)
Fold2
True Positive Rate
False Positive Rate
micro-average ROC curve (AUC = 1.00)
macro-average ROC curve (AUC = 1.00)
ROC curve of Covid19 class (AUC = 1.00)
ROC curve of Pneumonia class (AUC = 1.00)
ROC curve of Healthy class (AUC = 0.99)
Fold3
True Positive Rate
False Positive Rate
micro-average ROC curve (AUC = 0.94)
macro-average ROC curve (AUC = 0.95)
ROC curve of Covid19 class (AUC = 0.93)
ROC curve of Pneumonia class (AUC = 0.98)
ROC curve of Healthy class (AUC = 0.93)
Fold4
True Positive Rate
False Positive Rate
micro-average ROC curve (AUC = 0.97)
macro-average ROC curve (AUC = 0.97)
ROC curve of Covid19 class (AUC = 0.96)
ROC curve of Pneumonia class (AUC = 0.97)
ROC curve of Healthy class (AUC = 0.98)
Fold5
True Positive Rate
False Positive Rate
micro-average ROC curve (AUC = 0.96)
macro-average ROC curve (AUC = 0.98)
ROC curve of Covid19 class (AUC = 0.97)
ROC curve of Pneumonia class (AUC = 0.97)
ROC curve of Healthy class (AUC = 0.99)

4×5 −Fold Cross-Validation

Learning curves

Fold1

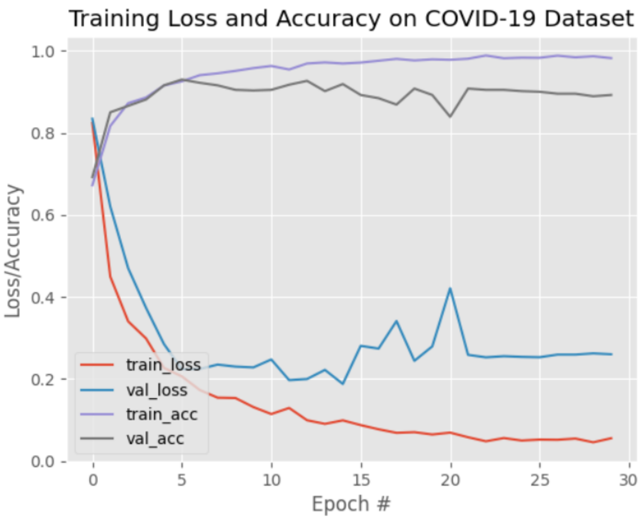

Fold2

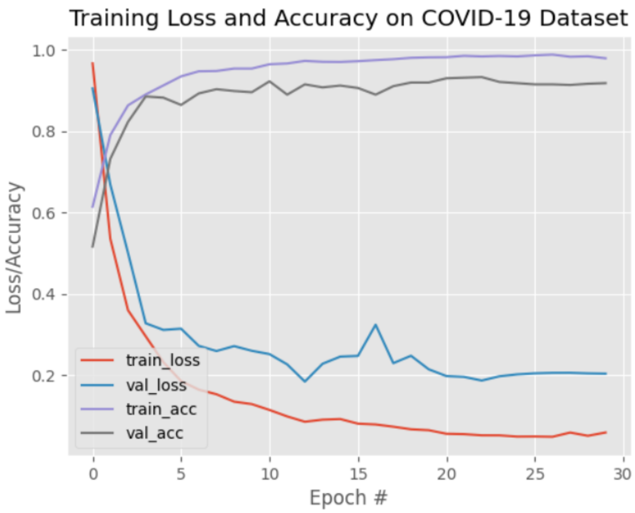

Fold3

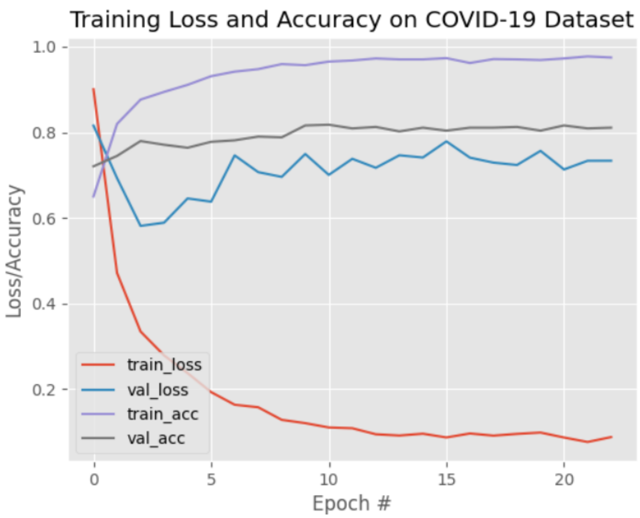

Fold4

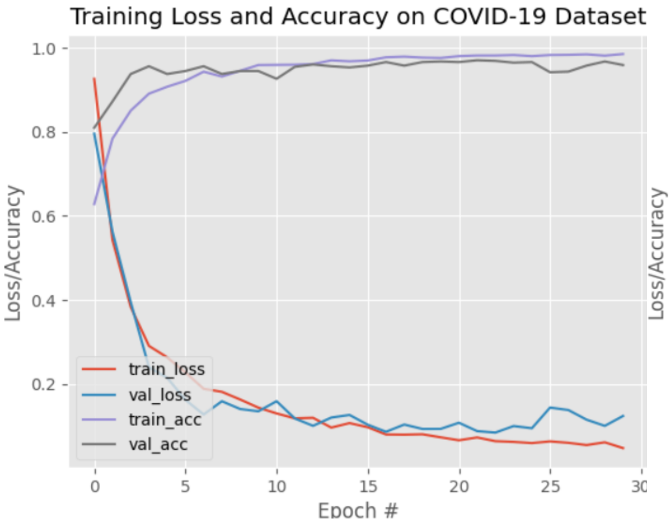

Fold5

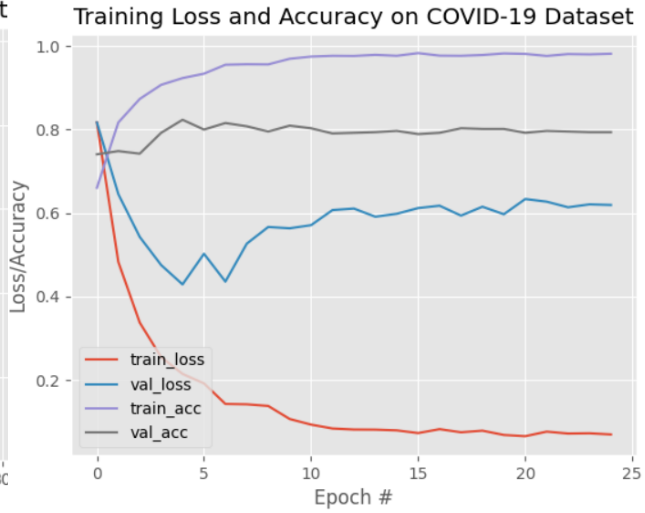

Confusion matrices

Fold1

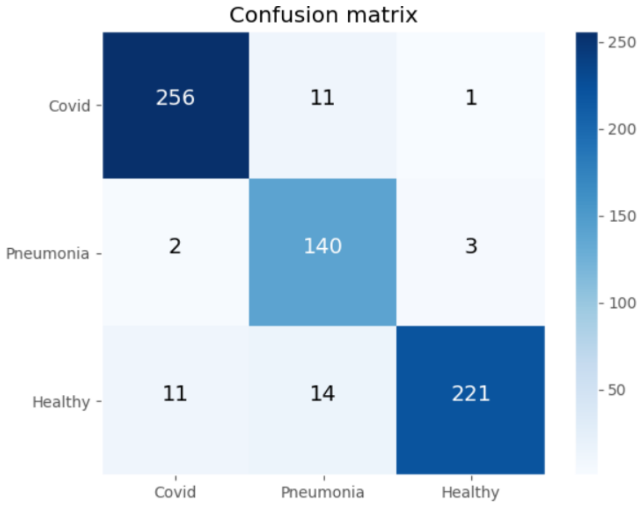

Fold2

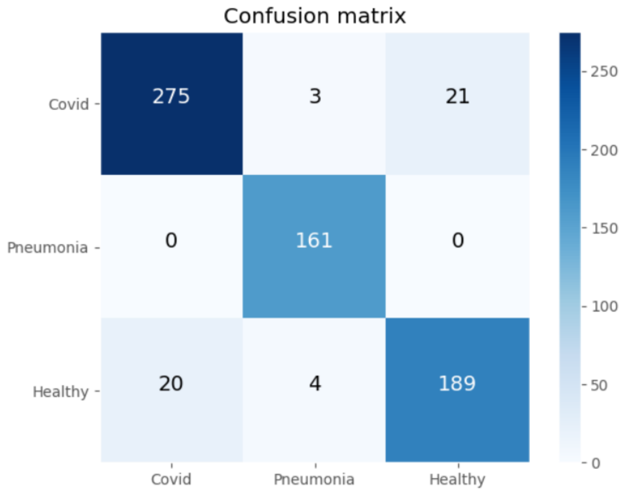

Fold3

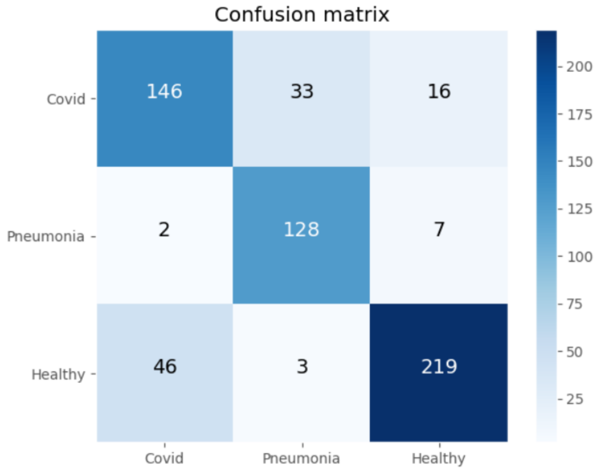

Fold4

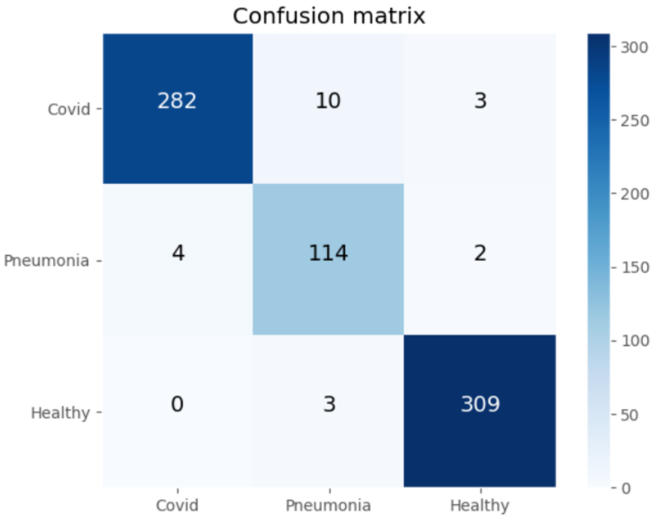

Fold5

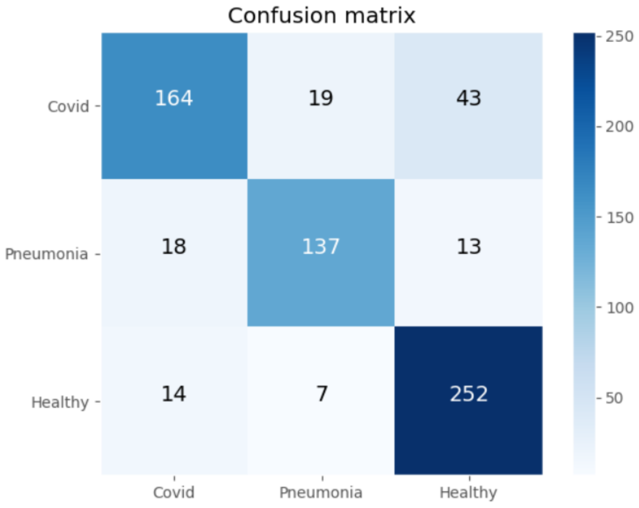

ROC curves

Fold1

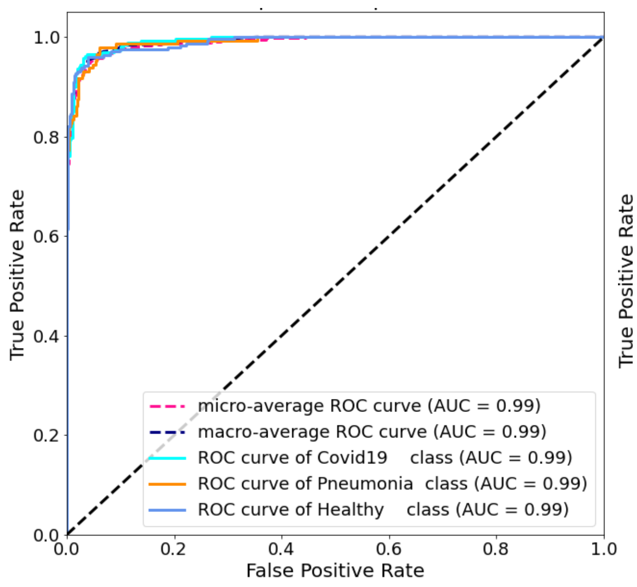

Fold2

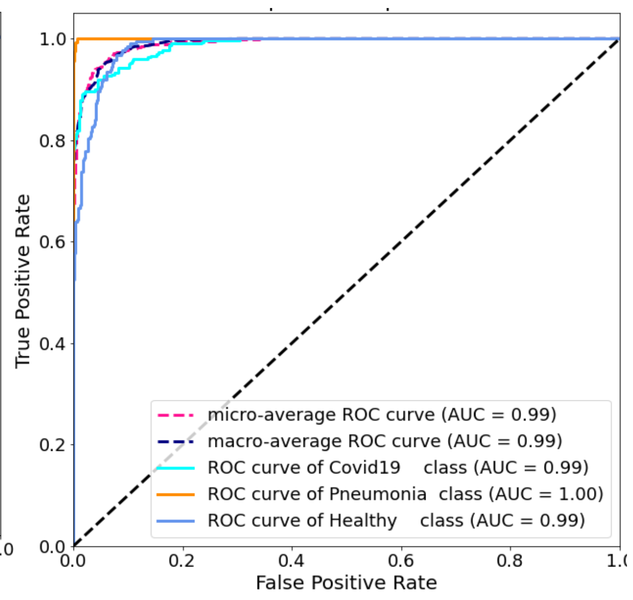

Fold3

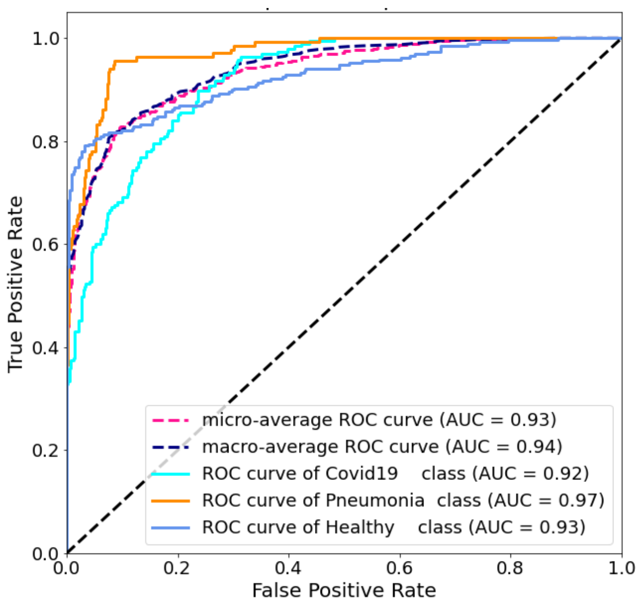

Fold4

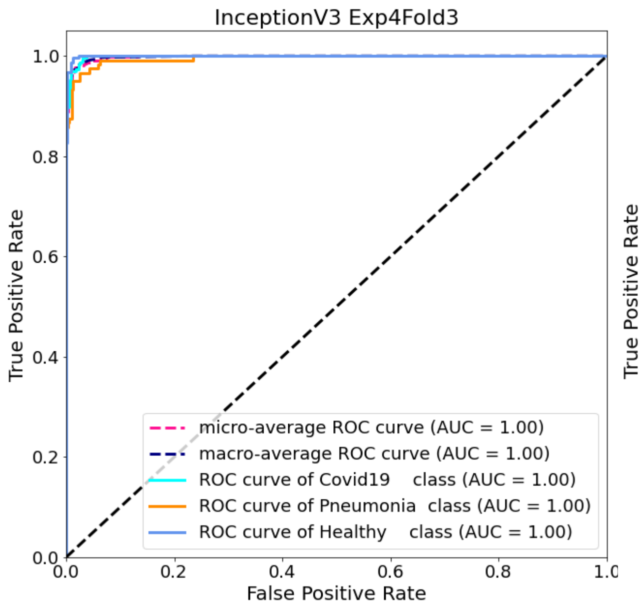

Fold5

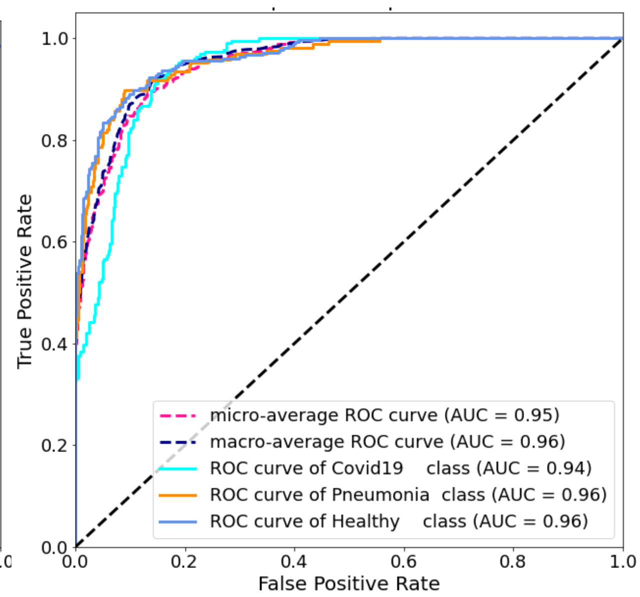

5 × 5 −Fold Cross-Validation

Learning curves

Fold1

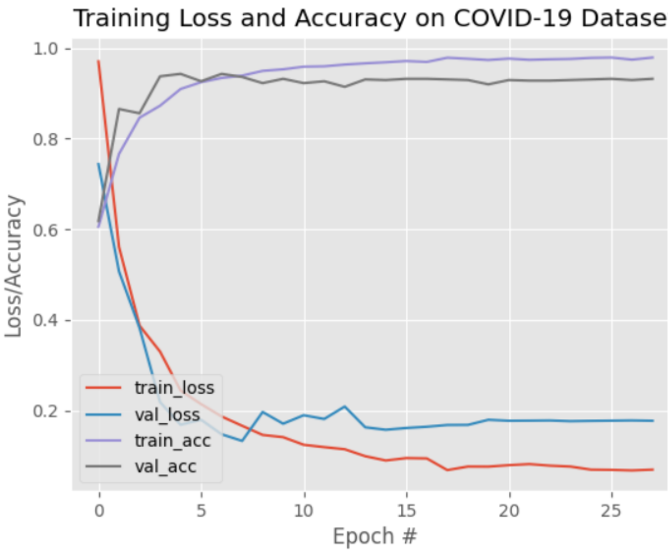

Fold2

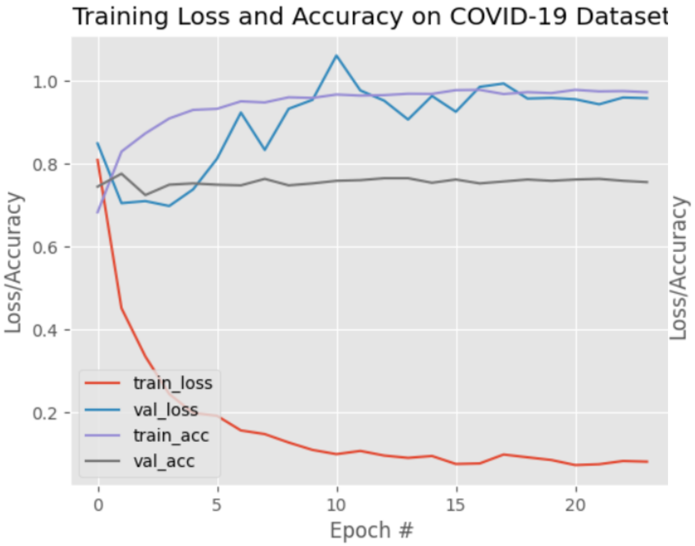

Fold3

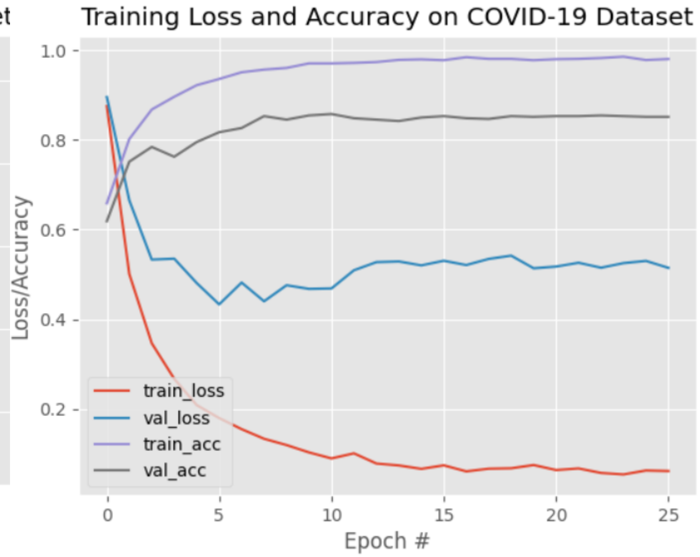

Fold4

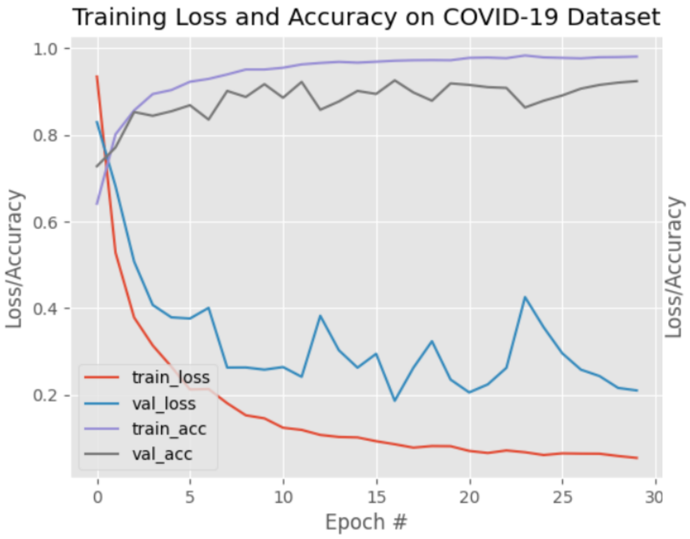

Fold5

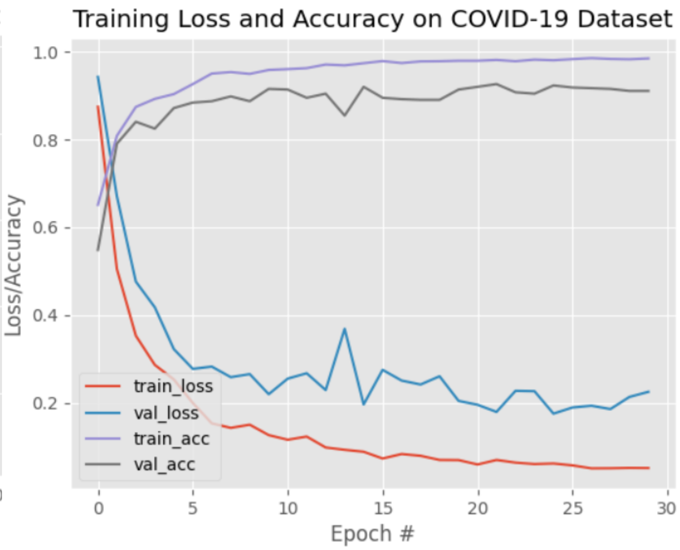

Confusion matrices

Fold1

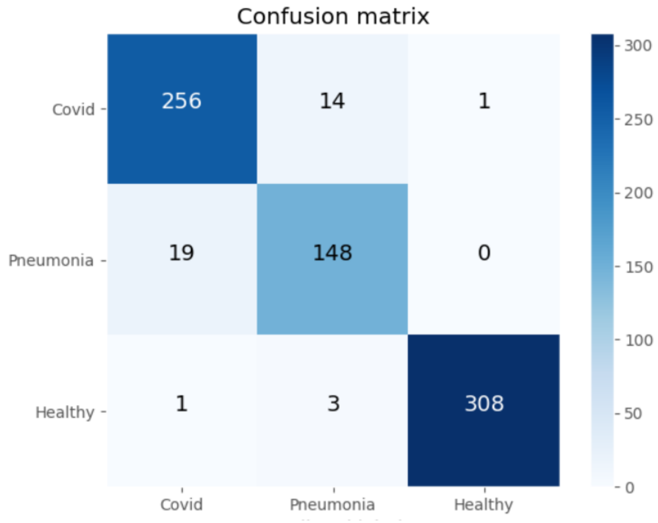

Fold2

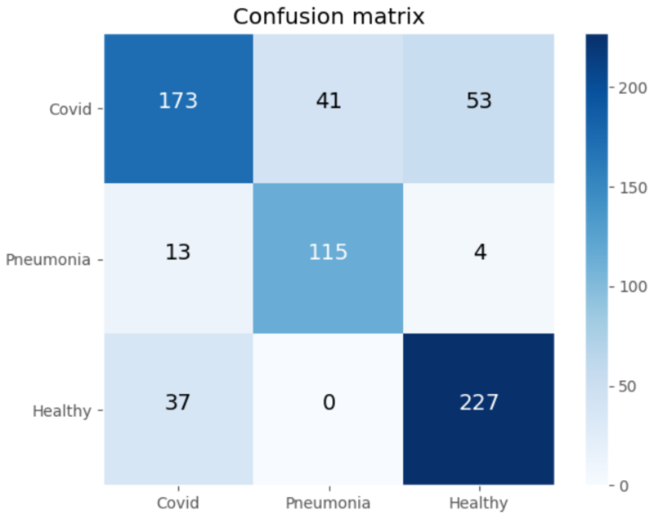

Fold3

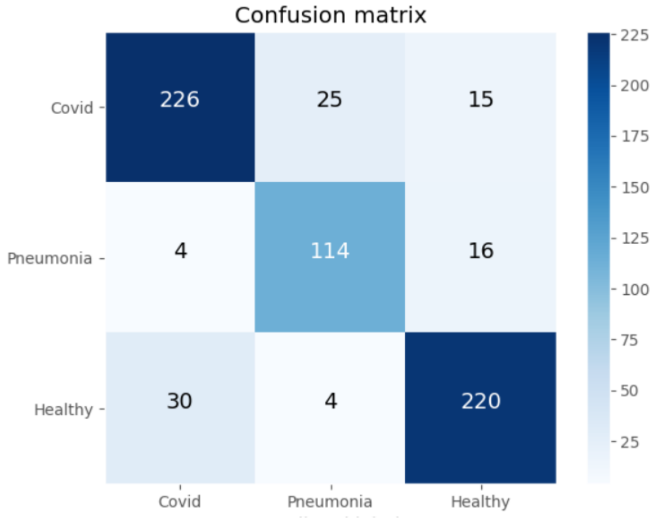

Fold4

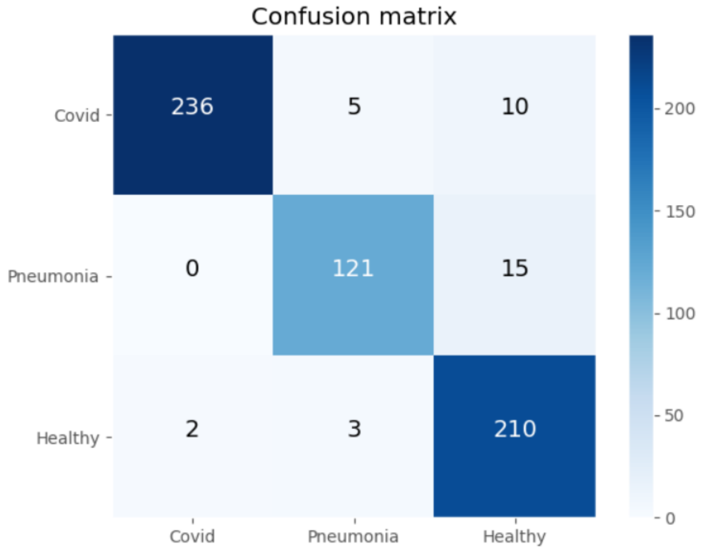

Fold5

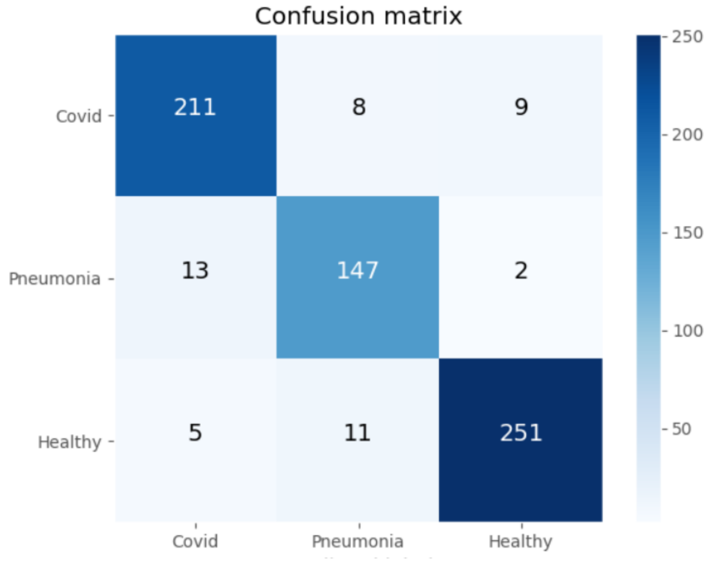

ROC curves

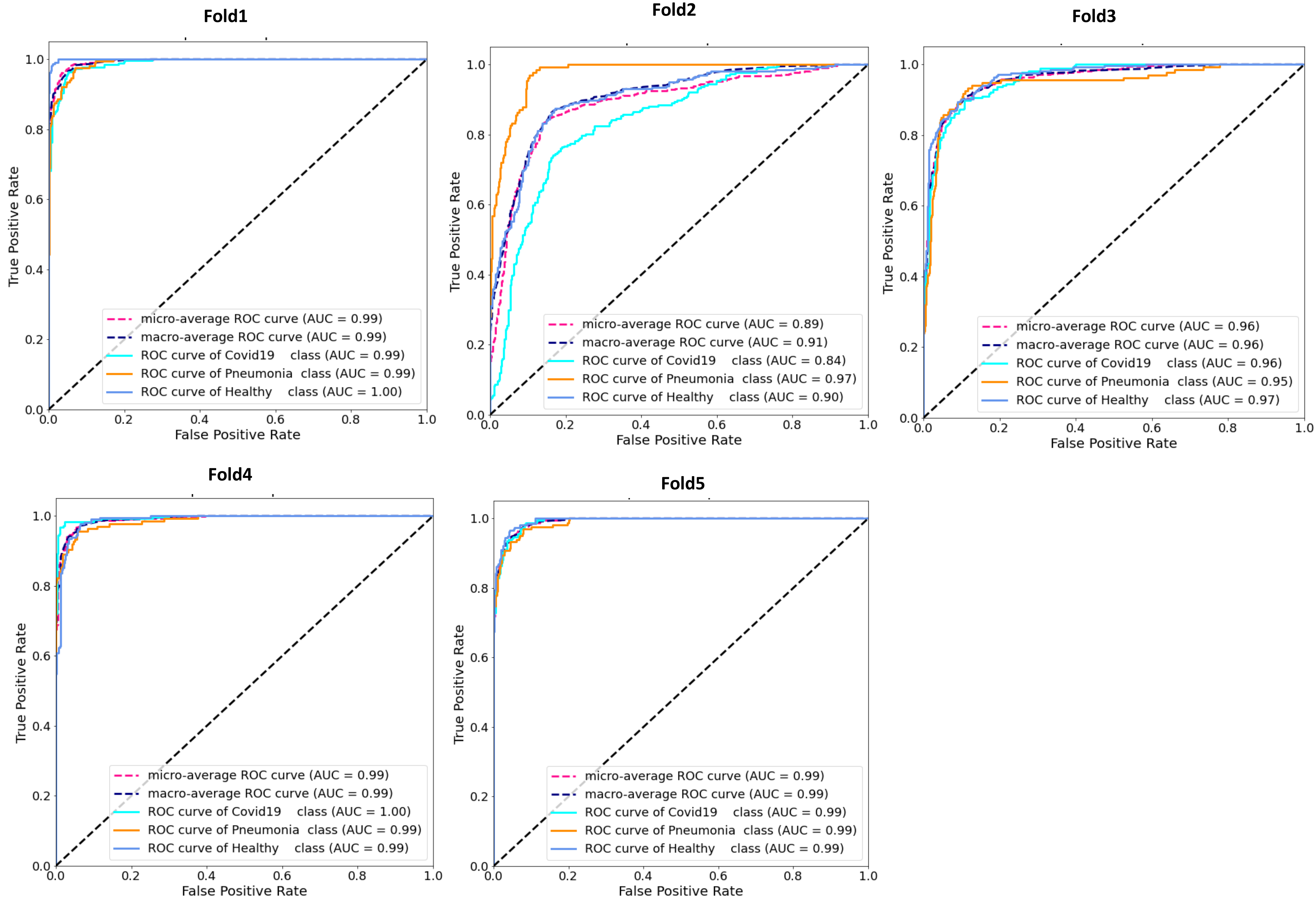

Fold1
True Positive Rate
False Positive Rate
micro-average ROC curve (AUC = 0.99)
macro-average ROC curve (AUC = 0.99)
ROC curve of Covid19 class (AUC = 0.99)
ROC curve of Pneumonia class (AUC = 0.99)
ROC curve of Healthy class (AUC = 1.00)
Fold2
True Positive Rate
False Positive Rate
micro-average ROC curve (AUC = 0.89)
macro-average ROC curve (AUC = 0.91)
ROC curve of Covid19 class (AUC = 0.84)
ROC curve of Pneumonia class (AUC = 0.97)
ROC curve of Healthy class (AUC = 0.90)
Fold3
True Positive Rate
False Positive Rate
micro-average ROC curve (AUC = 0.96)
macro-average ROC curve (AUC = 0.96)
ROC curve of Covid19 class (AUC = 0.96)
ROC curve of Pneumonia class (AUC = 0.95)
ROC curve of Healthy class (AUC = 0.97)
Fold4
True Positive Rate
False Positive Rate
micro-average ROC curve (AUC = 0.99)
macro-average ROC curve (AUC = 0.99)
ROC curve of Covid19 class (AUC = 1.00)
ROC curve of Pneumonia class (AUC = 0.99)
ROC curve of Healthy class (AUC = 0.99)
Fold5
True Positive Rate
False Positive Rate
micro-average ROC curve (AUC = 0.99)
macro-average ROC curve (AUC = 0.99)
ROC curve of Covid19 class (AUC = 0.99)
ROC curve of Pneumonia class (AUC = 0.99)
ROC curve of Healthy class (AUC = 0.99)
